# Molecular insights into pangenome localization and constructs design for *Hemophilus influenza* vaccine

**DOI:** 10.1038/s41598-025-03536-0

**Published:** 2025-07-01

**Authors:** Naila Zaman, Kainat Gul, Kinza Khurram, Syed Sikander Azam

**Affiliations:** Computational Biology Lab, National Center for Bioinformatics, Quaid-i- Azam University, Islamabad, 45320 Pakistan

**Keywords:** Haemophilus influenza, Multi-epitope vaccine, Immunoinformatics, Immune simulations, Molecular dynamics simulations, Computational biology and bioinformatics, Drug discovery

## Abstract

*Haemophilus influenza*, a major contributor to respiratory infections such as pneumonia, meningitis, sinusitis, chronic bronchitis, and acute otitis, poses a significant public health challenge, driven by rising antibiotic resistance particularly among the non-typeable *H. influenza* (NTHi) strains given their ability to evade immune surveillance. To address this, we employed a comprehensive immunoinformatics pipeline integrated with extensive pan-genome analysis of 59 strains of *H. influenzae* to design a novel multiepitope vaccine (MEV) candidate targeting most virulent and clinically significant proteins. Key surface exposed and virulence associated proteins, including Protein E, PilA, Protein D, P4, TolC, YadA, and HifC were prioritized based on their roles in bacterial adhesion, immune evasion, biofilm formation, and nutrient acquisition. Advanced in silico epitope prediction and verification strategies were utilized to map highly immunogenic regions across these proteins, followed by codon optimization to enhance expression efficiency in human systems. To further stabilize the vaccine construct, we performed disulfide engineering to enhance structural integrity and resilience. Comprehensive validation through in silico immune simulations, molecular dynamics (MD) simulations and binding free energy calculations confirmed the structural stability, immunogenic potential, and strong receptor affinity of the MEV candidate. Phylogenetic and virulence factor analysis further corroborated the broad coverage of the pathogenic relevance of the selected proteins. Together, our integrative approach presents a robust pipeline for rational vaccine design, offering a promising avenue toward combating multidrug resistant and immune evasive *H. influenza* strains.

**Supplementary Information:**

The online version contains supplementary material available at 10.1038/s41598-025-03536-0.

## Introduction

*Haemophilus influenzae is* a Gram negative, globally emerging pathogen that exclusively colonizes and infects the human respiratory tract and is a prevailing source of lung infections, septicemia, and meningitis^1^. According to the World Health Organization (WHO), *H. influenzae* is responsible for approximately 386,000 deaths anually worldwide^2^. *H. influenzae* strains are categorized into encapsulated and non-typeable *H. influenzae (*NTHi) strains, with the latter playing a predominant role in global disease diversification^3–5^. The high genetic variability among NTHi isolates is largely attributed to the horizontal gene transfer events^6–9^, which drive the evolution of their virulence repertoire. Numerous studies have investigated the genomic diversification of *H. influenzae*, linking it to increased drug resistance, virulent repertoire, and diffusion of pathogenesis due to the colonization of NTHi strains^10–12^. On the other hand, the recurrent and pervasive use of beta-lactams and ampicillin to curb this bacterium has led to resistance to this class of drugs, leading to a high mortality rate^13^. Consequently, to tackle the limited efficacy of the current Hib vaccines against NTHi strains, there is an urgent need to identify conserved pathogenic genes and their interacting partners to better understand the immunogenic response of antigens lying on the surface^14,15^.

Antibiotic resistance not only leads to increased morbidity and mortality but also results in significant economic burden prompting large scale sequencing of diverse bacterial strains. Extensive sequencing of strains has revealed the identification of bacterial species harbouring immense genomic diversity amid strains^16^ and for that purpose, pan-genome methods are exploited to analyze multiple genomes of the similar species, for example, Streptococcus spp. group B^17^. Pan-genome analysis enables comprehensive exploration of specie-specific virulence factors and is instrumental in studying epidemic outbreaks, analyzing core genome variability, and guiding vaccine development against bacterial pathogens^18–21^.

Historically, the field of vaccinology, guided by Pasteur’s principles, has seen the development of several effective vaccines against diseases such as mumps, measles, rubella, and Bacillus Calmette-Guerin (BCG) for *Mycobacterium tuberculosis*^22^. At the same time, the absence of a suitable culturing environment, widespread antigenic variations, and molecular imitation has limited the broad application of conventional culture-based practices^23^. These shortcomings led to the investigation of novel high throughput techniques that grabbed the attention to rush the pre-vaccine development period^24^ and resulted in the design of subunit vaccine candidates that aimed at cellular components, which are frequently discovered as virulence factors^23^. Shotgun sequencing and bioinformatics techniques are currently employed to identify potential subcellular locations, ranging from cytoplasmic to extracellular compartments, which is critical for rational vaccine design^25,26^.

**Fig. 1:**
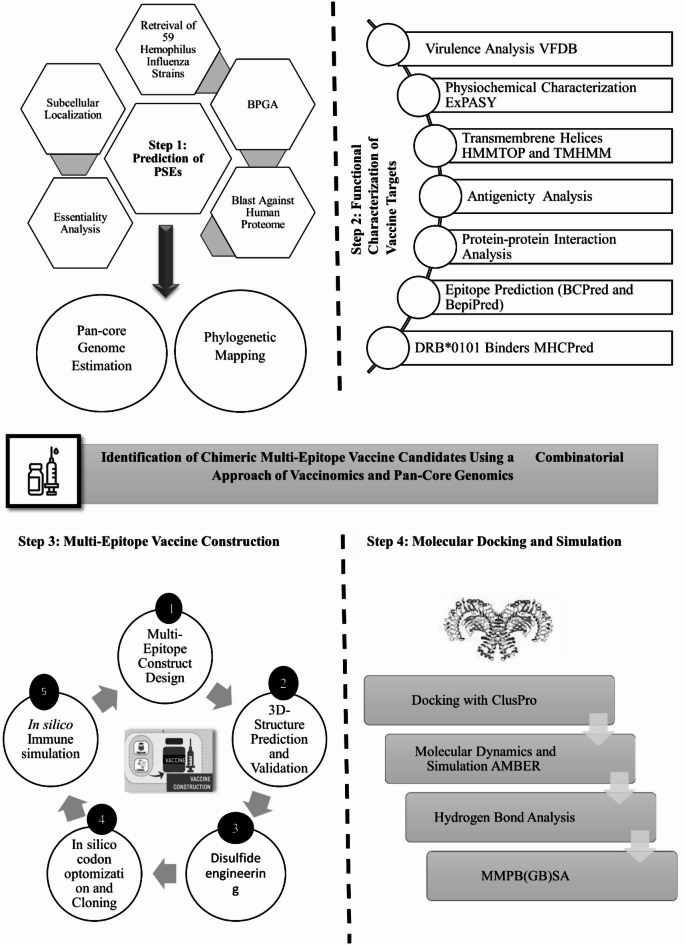
Complete flowchart of the study to identify the conserved vaccine candidates against the 59 H. influenzae strains.

Among these approaches, reverse vaccinology (RV) has proven to be particularly effective in identifying promising vaccine candidates across a broad spectrum of bacterial pathogens, including *Streptococcus pneumoniae*, *Streptococcus agalactiae*, *Staphylococcus aureus*, *Porphyromonas gingivalis*, *Chlamydia pneumoniae*, and *Leptospira interrogans*^17,27–33^. It has successfully categorized five appropriate serogroup B meningococcal vaccine candidates^34^. Initially, RV efforts were directed on a particular genome of a pathogenic strain or species, limiting the development of collective vaccine containing biologically cross-protecting antigens for multiple serovars or strains. However, to obtain more comprehensive outcomes, pan-genomic centered RV has been proven more powerful compared to the classical RV^31^. Furthermore, several studies have reported the counteract strategies to cater NTHi strains that vigorously regulate the immune response by controlling hemagglutinating pili and other surface adhesions, thus inciting the need to identify the conserved *H. influenzae* surface exposed proteins *(*PSEs)^12,35–38^. Thus, the work reported herein has expended in silico RV strategy combined with a pan-genome analysis of 59 strains of *H. influenzae* to screen the core PSEs as novel vaccine candidates. Furthermore, to get insights into the transfer of virulent elements that contribute to the drug resistance and the mechanisms controlling the colonization of this pathogen among different serovars, we mapped our results to the previously conducted studies to understand the pattern of pan-genome evolution^7,8,10,11^. Furthermore, the B and T cell epitopes were predicted along with their respective MHC alleles leading to the multi-epitope vaccine constructs^39,40^. We also analyzed the structural proteins of *H. influenzae* to identify potential antigenic and immunogenic epitopes capable of triggering both a humoral (B-cell) immune response and a cell-mediated (T-cell) immune response. We have proposed a multi-epitope vaccine candidate with the addition of appropriate adjuvant and linkers, taking potential epitopes from the screened proteins. The complete flowchart of the current study is presented in Fig. 1 that illustrates the combinatorial strategy for prediction of conserved vaccine candidates that will broaden the scope of *H. influenzae* vaccine design catering to both the encapsulated and non-encapsulated strains. We anticipate that our findings will contribute significantly to the advancement of next generation vaccine candidates; however, further experimental validation is essential to confirm the predicted outcomes.

## Materials and methods

### Pan and core-genome estimation

To initiate a comprehensive study on *H. influenzae*, fully sequenced genomes were prioritized and retrieved from the NCBI database^41^. Core and dispensable genome content, comprising the full pan genome were downloaded in GenBank format from the NCBI GenBank database (http://www.ncbi.nlm.nih.gov/genome). Pan-genome analysis was conducted to identify conserved putative PSEs and to quantify the number of core, accessory, unique, and exclusively absent genes; each of which can play a significant role in antigen prediction. For this analysis, the Bacterial Pan Genome Analysis (BPGA)^42^ tool was employed. The initial pre-processing involved clustering the genome sequences, followed by the exclusion of paralogous proteins using the USEARCH program of BPGA, which applies a default cut-off value of 50% sequence identity. The USEARCH algorithm is a fast, heuristic-based sequence clustering tool that groups sequences by pairwise identity using a greedy approach. It enables rapid identification of representative sequences with reduced computational load^43^. The clustered data generated through USEARCH was then used for pan-genome profile calculations^44^. Detailed statistics on accessory, unique, and exclusively absent genes predicted by BPGA are provided in Supplementary Table S1.

### Pathogenomics and phylogenetic mapping

In the current study, phylogenetic analysis was conducted through BPGA based on core gene alignments to construct a phylogenetic tree. The multiple sequence alignment was performed using MUSCLE, which implements a progressive alignment algorithm with k-mer counting and iterative refinement, followed by the construction of a phylogenetic tree using the Neighbor-Joining method based on the aligned sequences^45^. Supplementary Fig.S1 illustrates the distribution of selected strains and their mapping to the clonal lineages. However, to identify the essential virulent factors that are conserved across the species, the RV approach was used that is explained in the forthcoming section.

### Prediction of surface exposed proteins *(*PSEs)

To identify the essential, virulent, and antigenic PSEs among the core genome of *H. influenzae*, a reverse vaccinology (RV) approach was applied to the core gene dataset derived from BPGA analysis. The process initiated with the pre-screening of proteins based on four main criteria: (1) conservation across 59 strains of *H. influenzae* for broader therapeutic applications^46^ (2) non-homology to host (human) proteins^47^ (3) essentiality for bacterial survival and (4) surface exposure^48^. Redundant sequences were removed to get the non-redundant proteome to retrieve only the conserved targets across bacterial species [51]. These non-redundant proteins were then subjected to Blast-p^49^ analysis against the human proteome (Tax id: 9606) using stringent cut offs (E-value < 1e-5, bit score > 100, and sequence identity > 30%)^50^ to exclude homologous sequences and reduce the risk of host cross reactivity^47^. Following the extraction of non-homologous proteins, metabolic pathway analysis of essential proteins was carried out by KEGG Automatic Annotation Server (KAAS)^51^ to identify the unique proteins that are absent in host and are responsible for bacterial survival and pathogenicity^52^. Only those proteins were considered essential that fulfilled the criteria of having a 30% sequence identity, E-value < 1.0 E 4, and a bit score > 100^53^. To further refine the selection to surface exposed candidates, a comparative exoproteome analysis was carried out using PSORTb (https://www.psort.org/psortb/)^54^CELLO(https://cello.life.nctu.edu.tw/) and CELLO2GO (https://cello.life.nctu.edu.tw/cello2go/)^55^as proteins lying at the surface of the pathogen are crucial for vaccine development due to their ability to interact with the host and the subsequent cell cycle of the pathogen, which in turn generate the targeted immune responses^56^. PSORTb utilizes Support Vector Machine (SVM), Hidden Markov Models (HMM), and motif analysis for subcellular localization prediction^57^. In contrast, both CELLO and CELLO2GO employ SVM-based multi-class classifiers for protein subcellular localization and Gene Ontology (GO) annotation^58^. These screened proteins were subsequently analyzed in the second phase of RV to prioritize only those virulent proteins that meet the criteria of vaccinomics for vaccine development.

### Functional characterization of vaccine candidates

In the second phase of RV, the core Virulent factor database (VFDB) was used to identify the virulent proteins by running the Blastp against the database^59^.Those proteins were shortlisted that exhibited ≥ 30% sequence identity with a bit score of ≥ 100^60^. These virulent proteins were further subjected to the physiochemical characterization of the shortlisted proteins to calculate the important parameters like the in-stability index^61^ and molecular weight, which was performed by the ExPASY server(https://www.expasy.org/)^62^. The stability of the protein is calculated by measuring the instability index of a protein in a test tube revealing the presence of only those dipeptides that are absent in vivo^61^. The second property was to determine the molecular weight of the filtered proteins, which should lie < 110 kDa^63^ to effortlessly purify the vaccine candidates. Furthermore, the Topology data bank of transmembrane proteins, RRID: SCR_007964 HMMTOP and TMHMM(https://services.healthtech.dtu.dk/services/TMHMM-2.0/)^64^ were exploited to characterize proteins that contain less than or equal to 1 transmembrane helix as they can be easily purified and cloned^65^. Both tools utilize Hidden Markov Models (HMMs) to predict transmembrane helices and the topology of membrane proteins with high accuracy^65^ The selected proteins were next checked for their antigenic nature to particularly select only those targets that exhibit a high probability of binding to the antibodies including T-cell receptors using VaxiJen 2.0(https://www.ddg-pharmfac.net/vaxijen/VaxiJen/VaxiJen.html)^39^^,^^66^. Last but not the least, the protein interactions were evaluated to study relations with the host and to determine the crucial functional associations among all known proteins. It was carried out by a protein interaction database STRING (https://string-db.org/*).* Threshold values of interaction with a score of 0.7 were selected for the interaction analysis of the selected protein. Certainly, the protein interaction information has a significant role in understanding the inhibition impact of vaccine proteins on the pathogen^67^. However, the next step of this phase constitutes the prediction of B-cells and T-cells epitopes of the chosen proteins that is explained in the forthcoming analysis^68^.

### Epitope mapping

The immune-dominant B and T-cell epitopes induce immune responses giving rise to the multi-epitope peptide vaccine construct^69^, which overcomes the weak immunogenicity of peptide vaccines. The filtered proteins were therefore directed to the epitope mapping using BCPred(http://ailab.ist.psu.edu/bcpred/predict.html) and BepiPred(https://services.healthtech.dtu.dk/services/BepiPred-2.0/) to retrieve B-cell epitopes of 20-mer having the threshold value ≥ 0.5^70^. BCPred utilizes a Support Vector Machine (SVM) classifier based on subsequence kernel methods to predict linear B-cell epitopes^70^. In contrast, BepiPred combines Hidden Markov Models (HMMs) with a random forest algorithm trained on epitopes annotated from antibody-antigen crystal structures for improved accuracy^71^.Following this, MHCPred(https://www.ddg-pharmfac.net/mhcpred/MHCPred/) and IEDB server(https://www.iedb.org/) were used to direct B cell epitopes to T cell epitopes prediction^72^. MHCPred employs a QSAR-based approach using Partial Least Squares (PLS) regression to predict peptide-MHC binding affinities^73^. The IEDB server utilizes multiple algorithms, including SMM, ANN, and NetMHCpan, for robust T-cell epitope prediction. Binding of an antigen with both MHC I and MHC II class describes antigens’ affinity for T-cells^74^. T-cell epitopes having common MHC molecules with IC50 < 100 nm that have a high affinity for the DRB*1101 and DRB*0101 alleles were selected^73^. These antigenic epitopes were further evaluated for their antigenicity using the VaxiJen 2.0 server. VaxiJen employs an alignment-independent approach based on the Auto Cross Covariance (ACC) transformation of protein sequences, which evaluates the physicochemical properties of peptides to predict their antigenic potential^39^.Moreover, after the removal of allergic and Toxic sequences with AllerTOP 2.0(http://www.pharmfac.net/allertop/)^75^ ToxinPred(https://webs.iiitd.edu.in/raghava/toxinpred2/), the selected epitopes were used to design multi-epitope vaccine constructs. AllerTOP 2.0 utilizes an alignment-free method that transforms protein sequences into uniform vectors based on their physicochemical properties, and classifies them using the k-nearest neighbor (kNN) algorithm (k = 3). The model, trained on a balanced dataset of 2210 allergens and 2210 non-allergens from various species, demonstrates a high sensitivity of approximately 94%^76^. Similarly, ToxinPred employs a Support Vector Machine (SVM)-based classification approach, integrating features such as amino acid composition, dipeptide frequency, and various physicochemical descriptors to accurately predict peptide toxicity. Only epitopes classified as non-allergenic and non-toxic were selected for inclusion in the final multi-epitope vaccine construct^77^.

### Composition of a multi-epitope vaccine construct

Due to weak immunogenicity of peptide vaccines, it is suggested to design the multiple-epitope vaccine by the selection and combination of different epitopes of selected proteins and an appropriate adjuvant^69^. Therefore, a multi-epitope vaccine construct was designed to enhance the antigenicity of a single epitope by adding cholera toxin B subunit as an adjuvant because of its high affinity for receptors of macrophages and dendritic cells, EAAAK and GPGPG linkers^78^. EAAAK, which is a linker was used to join the cholera B toxin subunit (UniProtKB - E9RIX3 (E9RIX3_VIBCL) to the N terminal of epitope whereas, the epitopes were joined to each other using GPGPG linkers. Phyre2^79^ and RaptorX^80^ were used for the structure prediction of a construct. The constructed structure was then refined through ProSAweb^81^, PROCHECK, Verify-3D^82^, and ERRAT (https://servicesn.mbi.ucla.edu/ERRAT/) were further used for the evaluation of predicted structures.To improve the structural rigidity and stability of the designed vaccine constructs, disulfide engineering was performed using the Design 2.0 server^83^. Moreover, for **c**odon adaptation that provides a rate of foreign gene expression in the host especially when there is a difference in the usage of codon between host and the organism, the vaccine peptides constructs were subjected to the *Escherichia coli* K12 strain codon usage with the JCAT server^84^ where the nucleotide sequence was directed to snapgene (https://www.snapgene.com/)^85^ for its expression in *E.coli* pET28(+) vector.

### In silico immune simulations

Furthermore, the chimeric peptide underwent immune response profiling using in silico immune simulations via the C-ImmSim server (https://kraken.iac.rm.cnr.it/C-IMMSIM/) to characterize the immunogenicity^86^. For the prediction of immune epitopes, an agent-based model is employed that uses a position-specific scoring matrix (PSSM) and other machine learning methods to forecast molecular interactions. The simulations are run simultaneously for three compartments comprising bone marrow, thymus, and tertiary lymph nodes^87^. According to the TOVA^88^ that have been working in the vaccine development field for more than 25 years, 3 injections with the time interval of 4 weeks should be given. Thus, default simulation parameters were used with the time step fixed to 1, 84 and 168 and the number of antigen injections set to 1000 for each simulation. Three injections with 8 h apart (first injection given at time = 0) were given to simulate the actual humoral response to the repeated antigen exposure. Furthermore, the Simpson D was comprehended to infer the measure of diversity from the simulations plot^86^.

### Molecular docking of multi-epitope vaccine constructs

To interpret the binding affinity of an immune molecule to recognize virulent attributes of bacterial cell^89^, molecular docking of the vaccine construct was performed with TLR4 (PDB ID: 4G8 A), MHC-1 (PDB ID: 1I1Y) and MHC-2(PDB ID: 1 KG0)by using the ClusPro server(https://cluspro.org/home.php), which is used to dock two interacting molecules based on shape complementarity principle^90^. Docked complexes with the lowest energy scores were selected and subjected to 500 ns simulations using AMBER 16. To neutralize the system, 3 Na + ions were added, and the system was placed in a TIP3P water box with a 12 Å padding distance. Preprocessing consisted of three steps: first, the system was minimized for 500 cycles to relax the hydrogen atoms, followed by a 1000-cycle minimization of the water box. Second, the alpha-carbon atoms were minimized with a force constant of 5 kcal/mol·Å² for 1000 cycles, and third, non-heavy atoms were minimized with a force constant of 100 kcal/mol·Å² for 300 cycles. The system was then heated for 20 ps at 300 K, with a control of 5 kcal/mol·Å² on the alpha-carbon atoms. Langevin dynamics, with a gamma value of 1, was used to maintain the temperature, while the SHAKE algorithm was applied to constrain hydrogen bonding. The system was heated in an NVT ensemble. For pre-equilibration, the system was run for 100 ps with a 2 fs time step, followed by pressure equilibration using the isothermal-isobaric (NPT) ensemble, with restraints of 5 kcal/mol·Å² on the alpha-carbon atoms. The pressure phase was extended for an additional 50 ps, with a constraint of 1 kcal/mol·Å² per 10 ps. Finally, the system was equilibrated for 1 ns before performing the 100 ns production run with a cut-off value of 8.0 Å in the NVT ensemble. The resulting simulation trajectories were analyzed using AMBER16’s CPPTRAJ^91^ module was used and Xmgrace was used to produce a graphical representation of the results. To assess the conformational stability of the complexes, factors such as RMSD, RMSF, and hydrogen bond analysis between TLR4 and the vaccine constructs were conducted.

### Binding free energy

To determine the binding free energy values, change between the free energy of the complex and receptor was calculated using MMPBSA.py of AMBER. To study the strong interactions between the receptor and vaccine constructs, the free binding energy of each complex was carried out that has a significant role in complex stability. However, using the following equations solved the values of ΔG:

ΔG_bind,__solv_ = ΔG_bind,_ _vaccum_+ ΔG_solv,_ _complex_ – ΔG_solv, ligand_ – ΔG_solv, complex_ (1).

ΔG_solv_ = ΔG_electrostatic_ (ϵ80 − 1) + ΔG_hydrophobic_ (2).

ΔG_vaccum_ = ΔE_molecular mechanics_ – T.ΔG_normal mode analysis_ (3).

## Results and discussion

### Pan and core-genome estimation

To screen the core, virulent and essential proteins in 59 strains of *H. influenzae*, the complete dataset was subjected to the RV pipeline containing approximately 101,347 proteins. Due to the ability of a vast number of antibiotics to target significant cellular processes, the recognition of essential proteins hold a critical place in the selection of vaccine targets^24^. In the first phase, complete protein sequences were checked for homology to remove the redundant proteins with the BPGA. 88,365 protein sequences exhibiting sequence identity > 50% were therefore discarded from the dataset resulting in 12,982 non-paralogous proteins. Furthermore, these non-redundant protein sequences that are contemplated as critical targets in drug and vaccine discovery process were subjected to Blastp against the human proteome to shortlist non-human homologues. It is advisable to ignore human homologues that impact rather negatively the host cells and tissues leading to the intervention of autoimmunity^92^. Thus, the pipeline resulted in the screening of 3540 human homologous proteins and 9442 non-human homologous proteins, which were chosen for the essentiality check. The essentiality check revealed 2983 non-essential proteins that were discarded and 6459 essential proteins were therefore selected for the next step. Moreover, the essential proteins that lied at the surface of the pathogen were screened as they offer protection to the immune system against infections and are effective vaccine targets^93^. Consequently, after employing the subcellular localization and metabolic pathway analysis of selected proteins, data set came down to only 13 proteins that were extracellular as well as unique to the pathogen. Cytoplasmic proteins are disregarded when screening targets for vaccine design as they are placed intracellularly and are therefore difficult to access by the host immune system^94^. Moreover, the nature of these proteins is extremely enzymatic containing hydrophobic pockets for drug binding that aids in catalyzing cellular processes and controlling different biological functions^95^. Whereas, the extracellular proteins having exposed antigenic epitopes are easily distinguishable by the host immune cells resulting in the efficient and targeted response to immune reactions^96^.

### Virulence potential of *H. influenzae*

Several virulent factors of *H. influenzae* are known to date such as high molecular weight proteins HMW1 and HMW2, which are mostly found in 75–80% of all the strains. Hia adhesion proteins that are found in 20% of the strains^97–99^. Other important virulence factors are IgA1 proteases that disable human immunoglobin A1 and colonize the mucus are present in 95% of NTHi strains [96] [97].In the current study, the screened 13 PSEs were then checked for virulence and subjected to Blastp against the VFDB database as the virulent proteins can be the potential vaccine candidates due to their captivating nature in the formation of infections leading to pathological conditions^100^. It was revealed that only 7 proteins out of 13 outer membrane proteins hold the ability to initiate infectious pathways. However, among the 13 extracellular proteins, a few other significant virulent proteins were also found which came out as a result of vaccinomics and the pan-core genome pipeline. The presence/absence of virulence factors of *H. influenzae* in specific strains belonging to a particular clade are presented in Table 1. We further mapped the screened virulent genes with the phylogenetic tree to evaluate the potential of certain strains correlated with a specific clade.

**Table 1:** Important virulence factors of H. influenzae and their distribution in different strains of clade V and clade VI are displayed as a result of blastp against VFDB. The + and – signs denote the presence or absence of genes in a particular strain.

Virulence Factors	Clade V			Clade VI					
	PittEE (NTHi)	86-028 NP (NTHi)	Hi375	PittGG (NTHi)	R2866	R3021	Rdkw20	F3031	F3047
hif	**-**	**-**		**+**	**+**	**-**	**-**	**+**	**+**
Hia/hsf	**-**	**-**	**+**	**+**	**+**	**-**	**+**	**-**	**-**
HMW1	**+**	**+**	**-**	**-**	**-**	**-**	**-**	**-**	**-**
HMW2	**+**	**+**	**-**	**-**	**-**	**-**	**-**	**-**	**-**
P5	**+**	**+**	**-**	**-**	**-**	**-**	**+**	**-**	**-**
LOS	**+**	**+**		**+**	**+**	**-**	**+**	**-**	**-**
IgA	**+**	**+**	**+**	**+**	**+**	**+**	**+**	**+**	**+**

### Phylogenetic analysis

The phylogenetic analysis conducted in this study was mapped with the previously reported *H. influenzae* clades for the identification of conserved virulent genes along with the number of accessory and unique genes to determine the pattern of evolution of PSEs^7^. The study conducted by De Chiara et al.. illustrated the pattern of evolution among different strains of *H. inflluenzae*, which resulted in the formation of VI clades giving rise to the construction of a phylogenetic tree based on core-SNPs. However, in this study, we observed that the selected 59 strains mostly belonged to the Clade V and VI of *H. inflluenzae* phylogenetic tree as presented in Supplementary Fig S1. The results revealed that the clades V and VI hold the reference strain Rd KW20 along with R2846, PittGG, F3031, F3047 CGSHiCZ412602, Hi375, 86 028 NP and 10P129H1, which were evolutionary more conserved than the other clades and were also lying closely in the evolutionary tree. These results suggest that these two clades could be genetically less diverse signifying less chances of change during persistence and more chances of adaptability to the environment. However, it was also observed that the strains 5P28H1/5P54H1, 6P24H2/6P32H1, 48P106H1/48P153H1, and 67P38H1/67P56H1 belonged to the chronic obstructive pulmonary disease (COPD)^101^. The previous studies proposed 12 vaccine candidates for COPD that did not change during persistence and displayed stability under different environmental conditions^101^. P4 and OPM proteins that are also a part of current study are one of the vaccine candidates for COPD proposed previously with low allelic diversity. Thus, the virulent genes identified in this study holding some well-known vaccine targets verify the reliability of the RV pipeline and pan-core genome analysis.

### Pan-core genome estimation

**Fig. 2:**
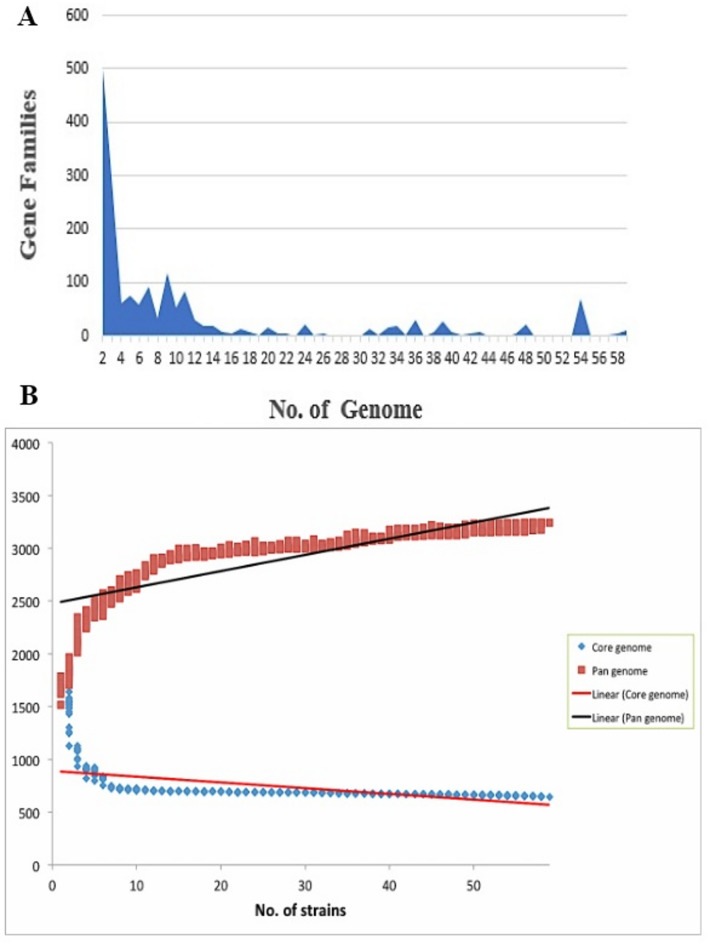
**(A)** The number of core gene families identified across 59 strains of H. influenzae is shown. The x-axis represents the genomes selected for the study, while the y-axis indicates the number of gene families present in each genome. **(B)** A graphical representation of the core and pan-genomes of 59 H. influenzae strains. The x-axis represents the number of strains, and the y-axis shows the number of genes in each strain. The black linear line illustrates pan-genome expansion with the addition of genomes, while the red linear line represents the stable and conserved core genome, which is critical for identifying vaccine targets as non-antigen and further subjected to the prediction of transmembrane helices by TMHMM^65^ and HMMTOP^109^. Proteins having only one transmembrane helices are preferably chosen for the vaccine design as they are easily cloned and facilitate the expressions analysis. Finally, the filtered proteins were submitted to protein-protein interaction analysis to investigate the potential metabolic functional relationship between other proteins.

To understand the functional variation at the genomic level, comprehensive pan-genome analysis can be greatly insightful. The pan-genome analysis of 59 strains of *H. influenzae* in the current study displayed an average comprising 1610 proteins with a GC content of 38% and 1,765 of the proteome. The pan-genome resulted in 3245 coding sequences in which 646 core proteins (19%) and 2123 accessory proteins (65%) were shared by all 59 strains. Whereas, the 476 unique proteins (14%) were found specifically in 9 strains. The addition of the dispensable genome from different strains makes the pan-genome large. It was observed that the dispensable genome was 79% of the total genome indicating a highly diverse nature of *H. influenzae* strains. The same results were identified in the previous studies displaying the genetic diversification of clinical expression in *H. influenzae* strains particularly NTHi strains [50]. Figure 2A suggested that the pan-genome size increases with an increase in genomes due to a larger contribution of accessory genes revealing an exponential decrease in the number of gene families. However, the gene enlargement curves illustrated in Fig. 2B revealed the exponential decay of curve that reached the equilibrium at 3000–3500 core proteins, suggesting the 59 strains chosen are adequate to estimate the core PSEs. Nevertheless, the presence of a large pool of dispensable genes in case of *H. influenzae* suggested a higher probability of frequent horizontal gene transfer events^102^. The strains belonging to the clade VI comprised the largest number of accessory genes whereas the maximum diversity was seen in the strain 10P129H1 of clade V with the highest number of unique proteins and exclusively absent proteins as presented in Supplementary Table S1. At the same time, the maximum numbers of genes families were observed in the strains 10P129H1, 84P36H1, and 86-028 NP. The strains 10P129H1 and 84P36H1 are the NTHi strains that were isolated from the COPD patients and are known for exhibiting the epigenetic regulators ModA15 and ModA18. These two strains carry a large number of virulent factors including HMW1 and HMW2 proteins and other adhesion proteins^103^. However, the NTHi strain 86-028 NP contains a large number of genes exhibiting a 4% larger genome as compared to the strain RD-KW20. It was also observed that the strains 10P129H1 and 84P36H1 lie next to each other in the phylogenetic tree whereas, all the three strains belong to clade V, indicating the utmost importance of clade V and VI in shaping the virulent repertoire of *H. influenzae.* Moreover, BPGA also predicted that the gene families identified in this study constituted 27% of total proteome suggesting a remarkable propensity of the pathogen to gain/lose genes, which could support a pathogen to survive in varied ecological niche^9,104^.

### Physicochemical characterization and evaluation

Furthermore, to select the most suitable candidates for vaccine design that can readily go into the experimental evaluation process, different physiochemical properties most importantly the antigenicity and number of transmembrane helices of 13 screened proteins were evaluated^63,68,105–107^. The calculation of molecular weight of the proteins is the most important factor as the smaller proteins are easier to purify^108^. Thus, all the 13 PSEs were filtered based on the fixed threshold value of < 110 kDa molecular weight. Out of 13, only 7 extracellular proteins fulfilled the criteria confirming the results from VFDB as displayed in Table 2, which were further subjected to allergenicity analysis. The allergenicity of the selected proteins was evaluated using AllerTOP to ensure that they do not contain peptides known to trigger allergic reactions^75^. Next, the antigenicity analysis was conducted to check the capability of virulent proteins that can bind the T cell receptors and antibodies. The value of antigenicity of the screened proteins was evaluated using VaxiJen server keeping the default parameters ‘bacteria’ and 0.5 as a threshold value^39^, which predicted all proteins.

**Table 2:** Shortlisted virulent proteins based on allergenicity, antigenicity and the number of transmembrane helices.

S.No	Protein ID	Protein Names	AllerTop	VaxiJen	TMHMM
1	> WP_010869219.1	TolC protein	Non-Allergen	Antigen	0
2	> BCB66386.1	Surface-adhesin protein E	Non-Allergen	Antigen	0
3	> OLV27437.1	YadA_anchor domain-containing protein	Non-Allergen	Antigen	0
4	> CAA04192.1	PilA	Non-Allergen	Antigen	0
5	> WP_005694367.1	Prepilin peptidase-dependent protein D	Non-Allergen	Antigen	1
6	> CAA83902.1	Outer membrane usher protein HifC	Non-Allergen	Antigen	0
7	> AAX87722.1	protein P4	Non-Allergen	Antigen	1

### Interactome analysis

**Fig. 3:**
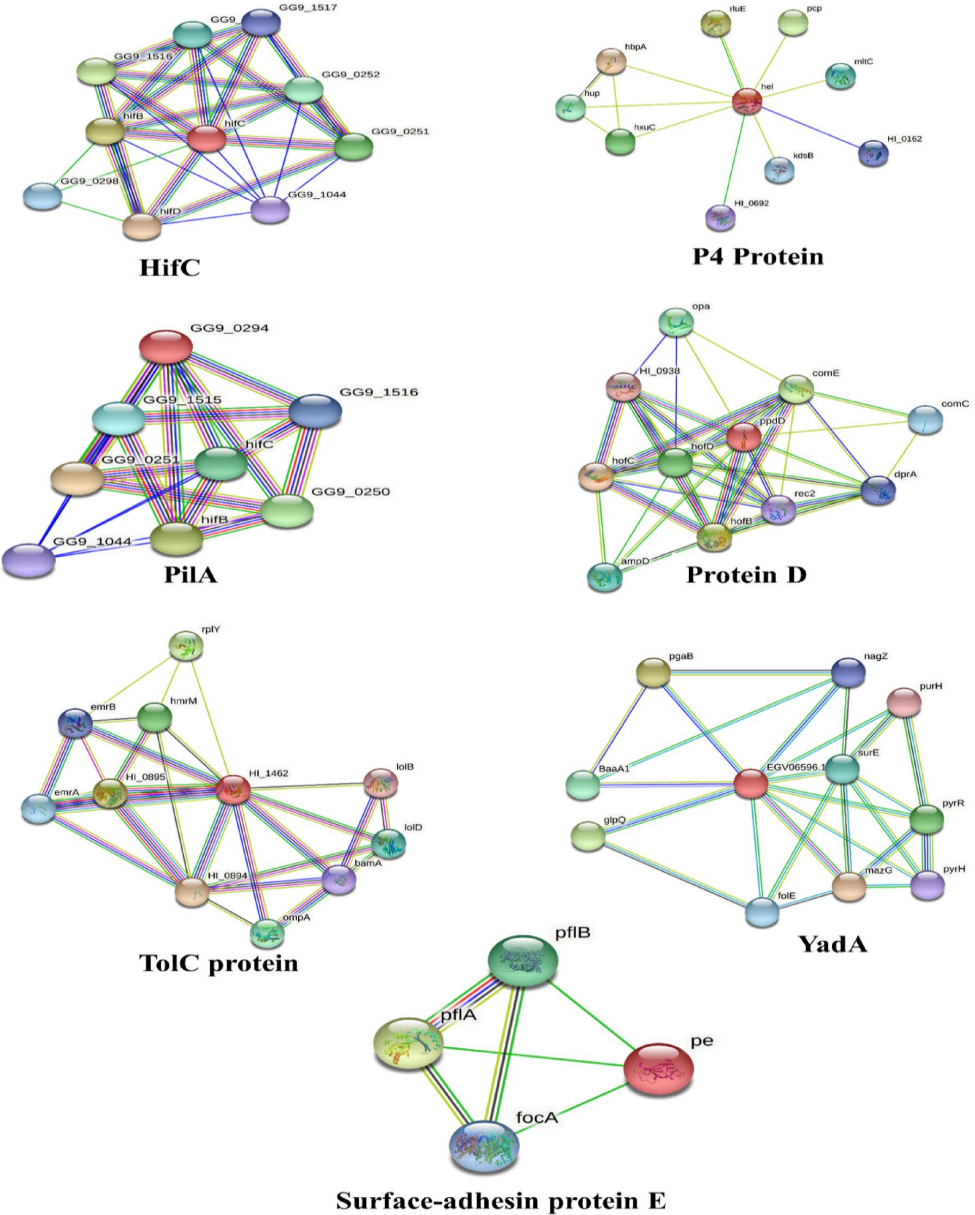
The interactions predicted between hub-bottleneck proteins (shown in red) and their neighboring proteins were retrieved from the STRING online database.

Protein interactions identify signal mediators and choke points as potential therapeutic targets by providing a wide range of details about metabolic and signaling pathways at the cellular level. The role of all filtered proteins was investigated based on their interaction with other proteins that are involved in different signaling pathways and cellular processes^110^. The protein association generated through STRING^111^ is displayed in Fig. 3. Based on central parameters of the network TolC protein, Surface-adhesin protein E, YadA_anchor domain-containing protein, PilA protein, Prepilin peptidase-dependent protein D, HifC and protein P4 are revealed as network hubs and bottlenecks (and also as hub-bottleneck elements). Evaluating protein-protein interactions offers valuable insights into the role of proteins within the broader protein network and their contribution to system-wide functions. Nevertheless, the interaction analysis underlines the role of proposed targets due to their involvement in pathogenesis as haemagglutinating pili, cell adhesion proteins and the transporter proteins. It is anticipated that the binding effect of predicted epitopes will affect the functional and biological impact of the interacting partners^112^, which compelled us to take all these proteins to vaccine designing and post processing phase to explore its potential as a vaccine candidate.

### Designing and post processing

#### B and T-cell epitope mapping

To stop the growth of a pathogen, the adaptive immune responses are extremely specialized and centered. The major players of the adaptive immunity that are responsible for generating the cell mediated responses in contradiction of foreign pathogens are the B and T cells lymphocytes^113^. Therefore, the prediction of B and T-cells epitopes is critical as the antibodies targeting these epitopes can in turn activate several mechanisms of cell mediated responses, which could be crucial for the growth and survival of a pathogen^114^. For that purpose, the critical sites for the binding of MHC I and MHC II were predicted for the B-cell and T-cell epitope mapping. The selection of MHC I and MHC II is based on the lowest percentile score from the predicted binding peptides. BCpred and BepiPred revealed 20-mer B-cell epitopes of all selected proteins as shown in Supplementary Table S2.

### Selection of B-cell epitopes

For both the B cell and T cell epitopes it is well known that antigen recognition and binding with MHC I and MHC II alleles describe antigen’s ability to generate an immune response^115^. For that purpose, MHCpred and IEDB analysis(MHC-II) were used for T-cell epitopes prediction that revealed 9-mer T-cell epitopes which were common to both classes of MHC and were selected based on IC_50_ value. Furthermore, the comparative analysis was carried out to select only common peptides from both the classes that were later checked for their binding affinities with the HLA-A*1101 and DRB1*0101 allele, an allele that is commonly residing in human population^63,68,105,116^. MHCpred was used to determine the binding strength of epitopes with DRB1*0101 on the basis of IC_50_ values. Robust immunological responses are expected from the epitopes displaying strong potential of binding^115^. Thus, to ensure the rigorous screening procedure, the epitopes having IC_50_ values < 100 nM were selected as they exhibit extraordinary results on competitive binding assay by displaying high affinity for T-cell alleles^93^. Lastly, the antigenicity, toxicity and allergenicity of the predicted DRB1*0101 binders from all selected protein was once again calculated to select only those that can effectively bind to the products of immune system^55^.The detailed results for the antigenicity, toxicity and allergenicity are displayed in Table 3.

**Table 3:** Antigenicity, toxicity and allergenicity predicted for shortlisted epitopes.

Protein	Selected Epitopes	Vaxigen (threshold = 0.5)	AllerTop v. 2.0	Toxin Pred
		Antigen value		
**TolC family protein**	HSYNQRITQ	1.2055(antigen)	non-allergen	Non-Toxin
	NWNTVKWNV	1.3326(antigen)	non-allergen	Non-Toxin
	SEADYETAR	1.2174(antigen)	non-allergen	Non-Toxin
	ASTVGTALH	1.0591(antigen)	non-allergen	Non-Toxin
	HKATAEDME	1.1008(antigen)	non-allergen	Non-Toxin
	KQYNVKENW	1.1967(antigen)	non-allergen	Non-Toxin
**Surface-adhesin protein E precursor**	KKQKKHTLS	0.8674 (antigen)	non-allergen	Non-Toxin
	GQGLRAAPK	1.1260(antigen)	non-allergen	Non-Toxin
	GKDGAKGET	3.1043(antigen)	non-allergen	Non-Toxin
	GKDGEKGEK	3.8102(antigen)	non-allergen	Non-Toxin
	DGKDGKDGK	3.9982(antigen)	non-allergen	Non-Toxin
	TVVNNNGIT	0.9099(antigen)	non-allergen	Non-Toxin
	GKDGKNAVA	1.3273(antigen)	non-allergen	Non-Toxin
	INITDGNGA	2.3089(antigen)	non-allergen	Non-Toxin
	TDGNGAVSS	2.3481(antigen)	non-allergen	Non-Toxin
	KNGKDGKNA	2.7932(antigen)	non-allergen	Non-Toxin
	ANITENNDG	1.2866(antigen)	non-allergen	Non-Toxin
	GKDGKNAVA	1.3273 (antigen)	non-allergen	Non-Toxin
	NGLNNGGNR	1.2794(antigen)	non-allergen	Non-Toxin
	VVNNNKLNS	0.8421(antigen)	non-allergen	Non-Toxin
	GLKFTGNNE	0.8390(antigen)	non-allergen	Non-Toxin
**Protein D**	TSCTGGKNG	3.2588(antigen)	non-allergen	Non-Toxin
**PilA**	GADPVATNK	0.9805 (antigen)	non-allergen	Non-Toxin
	DFTSENHNG	1.3933(antigen)	non-allergen	Non-Toxin
	CNVTTTNNK	1.7802(antigen)	non-allergen	Non-Toxin
	ENTGTNDSA	2.0610(antigen)	non-allergen	Non-Toxin
**Outer membrane usher protein**	ADSSYSRSG	1.4191(antigen)	non-allergen	Non-Toxin
	SSSGFTDKG	0.8171(antigen)	non-allergen	Non-Toxin
**Outer membrane protein P4**	NNKPFDGKD	1.1063(antigen)	non-allergen	Non-Toxin
	EEQANMQLQ	1.4953(antigen)	non-allergen	Non-Toxin
	KSEEQANMQ	1.4927(antigen)	non-allergen	Non-Toxin
	YFKKDTQGQ	1.0557(antigen)	non-allergen	Non-Toxin

### Designing of the multi-epitope chimeric vaccine constructs

In comparison to the vaccines based on large proteins, peptide-based vaccines have been proven an efficient alternative due to its small size, antigenicity, cost effectiveness and are therefore easy to use to in clinical practices. Furthermore, individual peptides have limitations in terms of antigenicity and provide poor coverage of T-cell alleles. To address this, the selected antigenic epitopes from all proteins were combined into a multi-epitope vaccine construct^114^. The five HIF-vaccine constructs that were constructed are shown in Table 4. The cholera toxin B subunit was used as an adjuvant, with an EAAAK linker connecting the adjuvant to the epitopes, while GPGPG linkers were employed to join individual epitopes together, creating a multi-epitope vaccine construct. CTB is widely recognized for its ability to enhance immune responses by facilitating antigen uptake and activation of immune cells^117^. The EAAAK linker provides structural rigidity and prevents steric hindrance between the adjuvant and epitope regions^118^, while the flexible GPGPG linkers help maintain proper epitope conformation and enhance antigen processing and presentation. Adjuvant is added to enhance the immunogenicity of the designed multiple-epitope vaccine construct, whereas the addition of linkers further facilitates the immune response by exposing the epitopes to the immune system thus preventing them from overlapping and folding^69,119,115^.

### Structure prediction and validation

Homology modeling is the in-silico method to predict the reliable 3D structural model of proteins sequences. Finalized five HIF vaccine constructs sequences were used to predict 3D structural models. This procedure was performed by utilizing the platforms: Phyre2 and RaptorX. The quality of the models was determined by generating a Ramachandran plot with PROCHECK. This server uses the Ramachandran plot to anticipate the chance of amino acids forming a secondary structure and to present the quality of models based on the fraction of amino acids in the allowed, favoured, and outlier regions. The validation was additionally checked using ERRAT and Verify 3D. The ProSA web server was utilized for additional assessment of the modeled structure’s quality. Models predicted from RaptroX server for all vaccine constructs were taken into consideration based on their overall quality factor and has better Ramachandran results as presented in Supplementary Table S3.

**Table 4:** Predicted multi-epitope-based vaccines for HIF.

S.NO	Vaccine Name	Multi-Epitope Chimeric Vaccine Construct
**1.**	*Haemophilus influenza* vaccine-1 (HIF-1)	**Protein E – PilA- protein D – P4** MIKLKFGVFFTVLLSSAYAHGTPQNITDLCAEYHNTQIYTLNDKIF SYTESLAGKREMAIITFKNGAIFQVEVPGSQHIDSQKKAIERMKDT LRIAYLTEAKVEKLCVWNNKTPHAIAAISMANEAAAKGQGLRAAPK GPGPGGADPVATNK GPGPGTSCTGGKNGGPGPGNNKPFDGKD
**2.**	*Haemophilus influenza* vaccine-2 (HIF-2)	**Protein E – PilA- protein D – P4 – TolC** MIKLKFGVFFTVLLSSAYAHGTPQNITDLCAEYHNTQIYTLNDKIF SYTESLAGKREMAIITFKNGAIFQVEVPGSQHIDSQKKAIERMKDT LRIAYLTEAKVEKLCVWNNKTPHAIAAISMANEAAAKGQGLRAAPK GPGPGGADPVATNK GPGPGTSCTGGKNGGPGPGNNKPFDGKDGPG PGHSYNQRITQ
**3.**	*Haemophilus influenza* vaccine-3 (HIF-3)	**Protein E – PilA- protein D – P4 – yaDA** MIKLKFGVFFTVLLSSAYAHGTPQNITDLCAEYHNTQIYTLNDKIF SYTESLAGKREMAIITFKNGAIFQVEVPGSQHIDSQKKAIERMKDT LRIAYLTEAKVEKLCVWNNKTPHAIAAISMANEAAAKGQGLRAAPK GPGPGGADPVATNKGPGPGTSCTGGKNGGPGPGNNKPFDGKDGPGP GDGKDGKDGK
**4.**	*Haemophilus influenza* vaccine-4 (HIF-4)	**Protein E – PilA- protein D – P4 – HifC** MIKLKFGVFFTVLLSSAYAHGTPQNITDLCAEYHNTQIYTLNDKIF SYTESLAGKREMAIITFKNGAIFQVEVPGSQHIDSQKKAIERMKDT LRIAYLTEAKVEKLCVWNNKTPHAIAAISMANEAAAKGQGLRAAPK GPGPGGADPVATNKGPGPGTSCTGGKNGGPGPGNNKPFDGKDGPGP GADSSYSRSG
**5.**	*Haemophilus influenza* vaccine-5 (HIF-5)	**Protein E – PilA- protein D – P4 – TolC – YaDA – HifC** MIKLKFGVFFTVLLSSAYAHGTPQNITDLCAEYHNTQIYTLNDKIF SYTESLAGKREMAIITFKNGAIFQVEVPGSQHIDSQKKAIERMKDT LRIAYLTEAKVEKLCVWNNKTPHAIAAISMANEAAAKGQGLRAAPK GPGPGGADPVATNKGPGPGTSCTGGKNGGPGPGNNKPFDGKDGPGP GHSYNQRITQGPGPGDGKDGKDGKGPGPGADSSYSRSG

### Structural stabilization via disulfide bond engineering

**Fig. 4:**
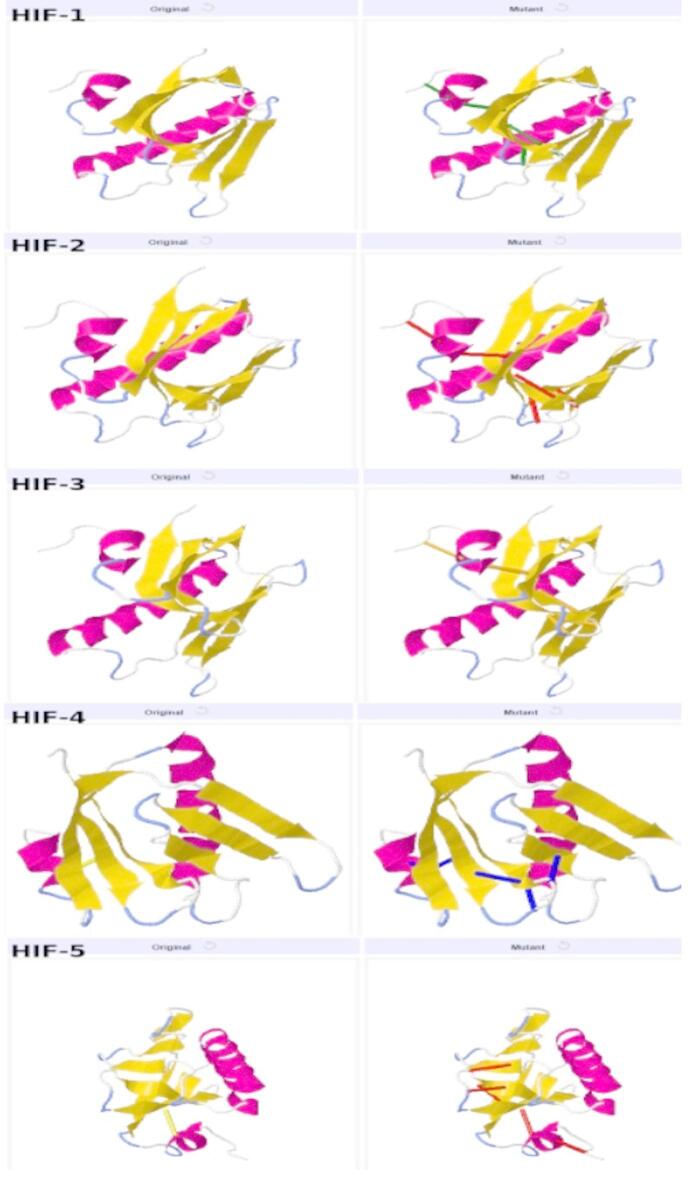
Disulfide Bond Engineering of HIF Vaccine Constructs (HIF-1, HIF-2, HIF-3, HIF-4, and HIF-5). The images display the original (left) and mutant (right) structures of each construct, highlighting the introduction of cysteine residues for disulfide bond formation.

To improve the structural rigidity and stability of the designed vaccine constructs^120^, disulfide engineering was performed using the Design 2.0 server (http://cptweb.cpt.wayne.edu/DbD2/index.php). Disulfide bond engineering was performed for all five HIF vaccine constructs (HIF-1, HIF-2, HIF-3, HIF-4, and HIF-5) to optimize molecular interactions and enhance stability by achieving precise geometric conformation. Six pairs of residues were selected for replacement with cysteine amino acids in each construct, based on favorable χ₃ angles and energy values that contribute to the stability of the protein. These residue pairs include Pro23–Leu29 (χ₃ angle: +100.71°, energy: 0.42 kcal/mol), Cys30–Cys107 (χ₃ angle: +80.84°, energy: 2.14 kcal/mol), Gln70–Pro114 (χ₃ angle: +119.88°, energy: 7.42 kcal/mol), Cys107–Ala119 (χ₃ angle: +105.86°, energy: 4.96 kcal/mol), Val108–His115 (χ₃ angle: +98.95°, energy: 4.80 kcal/mol), and Lys112–Ala116 (χ₃ angle: +97.20°, energy: 3.84 kcal/mol). The selection of these residues was intentional, including those with slightly higher energy levels (> 2 kcal/mol) or χ₃ angles outside the ideal range (< − 87 and > + 97), to enhance the stability of the constructs. As all five constructs were modeled using a conserved backbone structure with similar spatial arrangements, the predicted disulfide bond pairs and their associated geometrical parameters remained identical across the variants. This uniformity reflects the consistent structural framework employed during the design phase. These engineered disulfide bonds are expected to improve the conformational stability of the HIF constructs, ensuring their suitability for further immunological evaluation. The original and engineered disulfide-mutant HIF structures are shown in Fig. 4.

### Identification of discontinuous B-Cell epitopes on HIF vaccine constructs

**Fig. 5:**
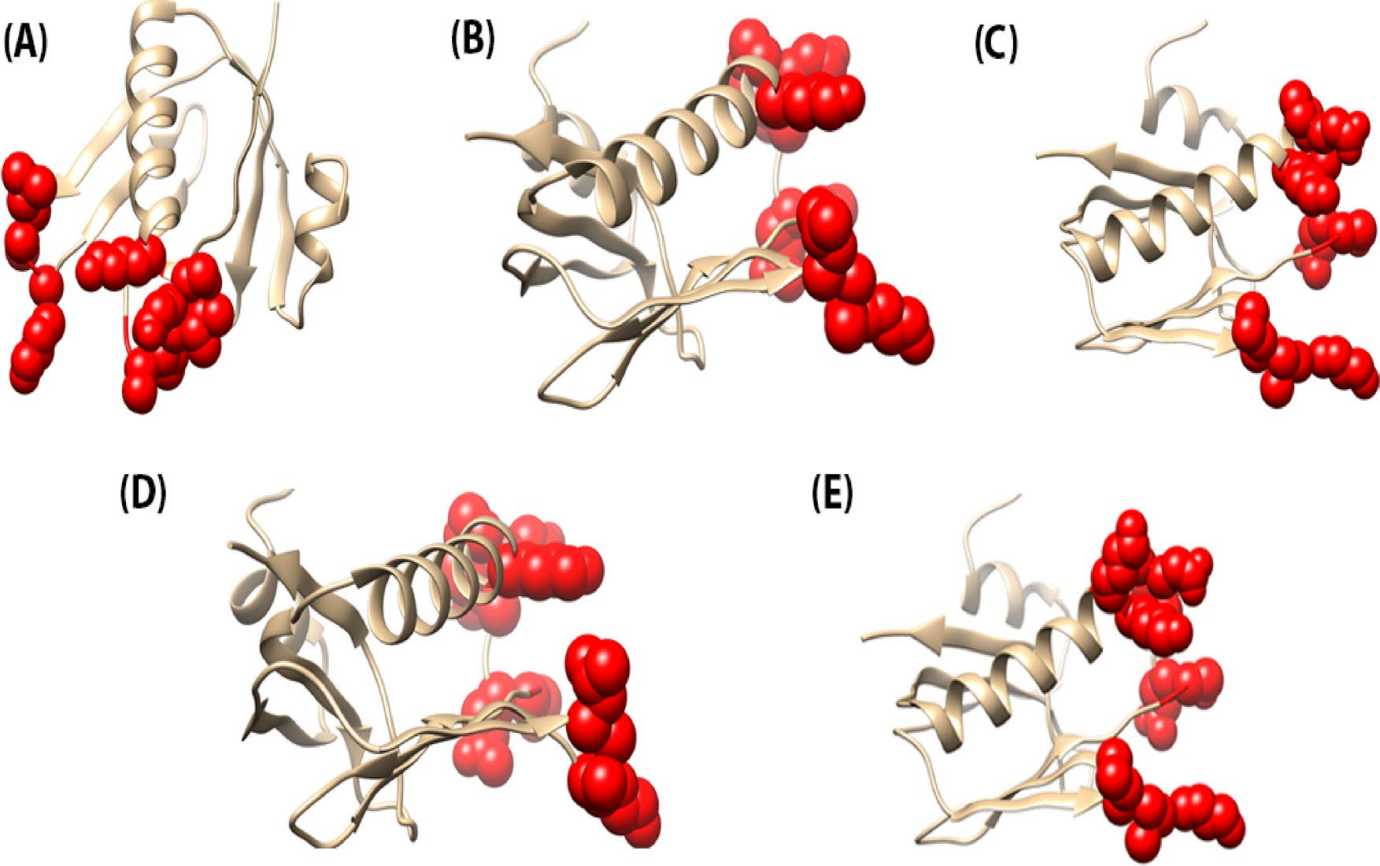
Predicted discontinuous B-cell epitopes mapped onto the 3D structures of the HIF vaccine constructs. (**A**) HIF-1, (**B**) HIF-2, (**C**) HIF-3, (**D**) HIF-4, and (**E**) HIF-5.

Discontinuous B-cell epitopes for all five HIF vaccine constructs were predicted using the DiscoTope 2.0 server, which integrates surface accessibility, residue spatial proximity, and amino acid propensity scores derived from the 3D structure. The analysis consistently identified 11 conformational B-cell epitope residues across all vaccine constructs. These residues were predominantly located in highly accessible regions on the protein surfaces, suggesting strong potential for effective B-cell recognition and antibody binding. The uniformity of the predicted epitopes across all constructs underscores the structural conservation of immunologically relevant regions within the vaccine designs. The identified epitopes were mapped onto the respective three-dimensional models, confirming their surface exposure and accessibility. The identified epitopes have been visually mapped onto the 3D structures of the vaccine constructs and are presented in Fig. 5 to illustrate their spatial distribution and surface exposure.

### Optimization and in silico cloning

**Fig. 6:**
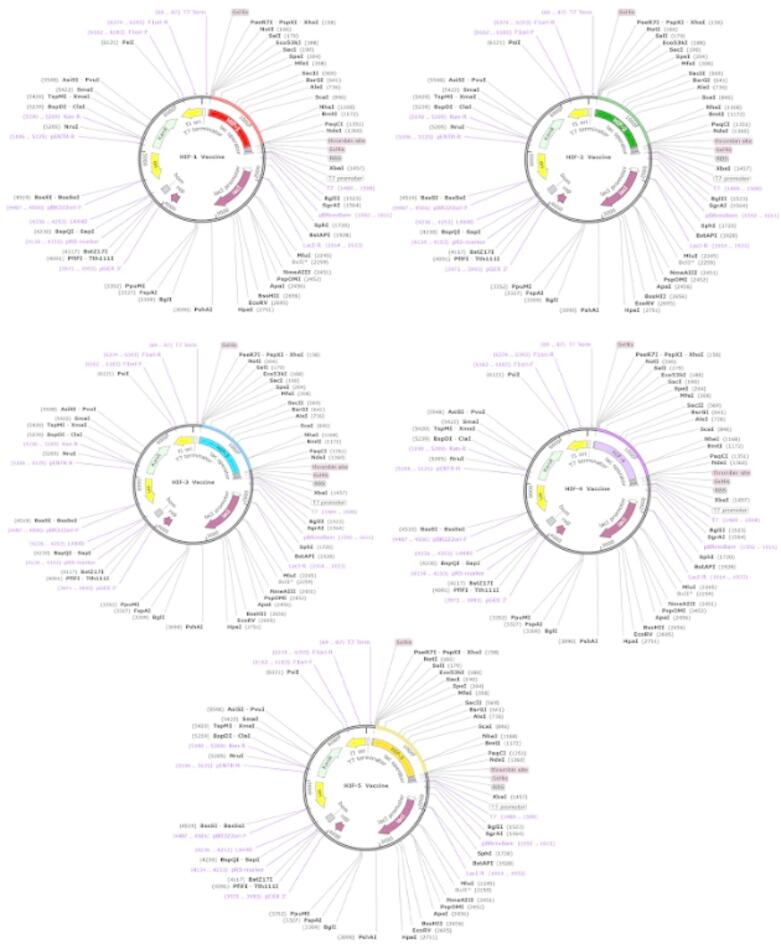
In silico restriction cloning of the codon-optimized final vaccine constructs into the pET-28a (+) vector was carried out between the XhoI (158) and NdeI (1360) restriction enzyme sites using SnapGene software. The final constructs are designed for efficient production in E. coli (strain K12).

A critical step in in-silico cloning is ensuring the efficient expression of vaccines within the *E. coli* expression system. The prioritized B-cell and T-cell predicted epitopes were combined with suitable adjuvants and linkers to create five vaccine constructs (HIF-1–5). These vaccine sequences were individually submitted to the Java Codon Adaptation Tool (JCAT) to optimize the codon usage for prokaryotic species. The analysis revealed that the DNA sequences for HIF-1–5 were 500, 550, 550, 590, and 650 nucleotides, respectively. The calculated Codon Adaptation Index (CAI) values (HIF-1 = 1.0, HIF-2 = 1.0, HIF-3 = 1.0, HIF-4 = 1.0, HIF-5 = 0.98) indicated that the adapted sequences contained codons compatible with the target organism’s cellular machinery. Additionally, the GC content of the optimized sequences ranged from 50.7 to 53%. These findings suggest that the designed vaccines will be efficiently expressed in *E. coli*. Restriction sites for XhoI and NdeI were incorporated at the N- and C-termini of the reverse-translated nucleotides of HIF-1–5, respectively. The final optimized sequences were then inserted into the pET28a (+) vector for cloning using SnapGene software (Fig. 6).

### In silico immune simulations

**Fig. 7:**
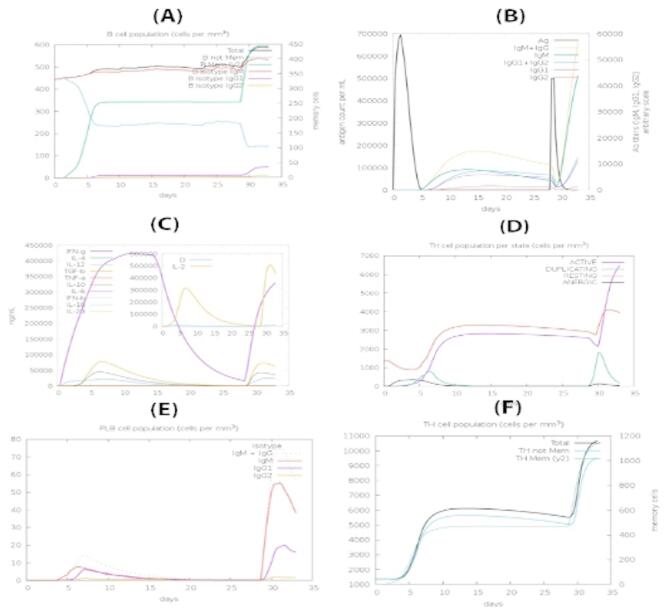
Prediction of immune responses against the HIF-1 construct: (**A**) Total B-cell responses, (**B**) Total antibody responses, (**C**) Interleukin responses, (**D**) Total T-cell responses, (**E**) Macrophage activity, (**F**) Dendritic cell dynamics.

Using a position specific score matrix (PSSM) and other machine learning techniques, the C-ImmSim server was used to do a computational immune simulation to determine whether a particular vaccine and immune interactions would have been immunogenic. The production of secondary and tertiary responses in comparison to the original response was significantly amplified by the antigen’s repeated exposures. To evaluate whether the response is sufficient for disease protection, the server produced a collection of graphic representations of each type of cell. After injecting the vaccine construct, a high degree of B-cell population was seen (Fig. 7A). Following the primary injection, a moderate rise in B-cell levels is observed, indicating initial immune activation. However, with secondary and tertiary exposures, there is a marked amplification in B-cell populations, particularly plasma and memory B cells, highlighting the development of immunological memory. This expansion signifies an enhanced and quicker antibody-mediated response upon re-exposure to the antigen. The consistent elevation in memory B cells suggests the vaccine’s potential for long-term protection, confirming its strong immunogenic profile and ability to elicit a robust and sustained humoral immune response.The secondary and tertiary immune responses illustrated by IgM was relatively high followed by the primary immune response, which also yielded escalations in B-cell populations and in levels of IgG1 + IgG2, IgM, and IgG + IgM antibodies with a marked decrease in the concentration of an antigen as presented in Fig. 7B. After the initial dose, a modest level of IgM is produced, representing the early-phase, primary response. With subsequent exposures, there is a significant surge in IgG1 + IgG2, IgM, and combined IgG + IgM levels, reflecting a potent and well-established secondary and tertiary response. This escalation indicates effective class switching and maturation of the humoral response. Notably, the rise in antibody titers is accompanied by a progressive decline in antigen concentration, demonstrating successful antigen recognition and clearance. These findings emphasize the vaccine’s ability to induce both an immediate and memory-driven antibody response, which is crucial for long-term immune protection Moreover, the results of in silico immune simulation yielded the production of immune memory, which upon successive exposures lead to the clearance of an antigen along with the increase in TH (helper) and TC (cytotoxic) cell populations Fig. 7C. Similarly, an expansion in IFN-g (> 400,000 ng/ml) was observed after the successive injections that remained at peak levels throughout the simulation time.

These findings imply an enhancement in immunity and memory, which will lead to a better antigen clearance upon re-exposure (Fig. 7C). In addition to this, the dynamic progression of immune responses revealed a notable enhancement in antigen clearance, which was faster with each subsequent exposure, suggesting the development of immunological memory and a more efficient immune system. The increased concentration of IFN-g and its sustained levels also point to a more effective activation of TH1 cells, which are critical in combating intracellular pathogens. The dynamic changes in the TH cell populations across different states (active, duplicating, resting, and anergic), with a significant increase in the active state following antigen exposure, indicating a robust immune response as shown in Fig. 7D. The transition of TH cells through various functional states, particularly the shift toward an active state post-antigen encounter, further reinforces the adaptive nature of the immune response and highlights the role of TH cells in mediating antigen clearance. Figure 7E illustrates macrophage activity, with a focus on the balance between resting and active macrophage populations over time. Post-vaccine administration, there is a distinct increase in active macrophages, particularly after booster doses. This indicates robust activation of the innate immune system, which is essential for early antigen recognition and presentation. Active macrophages play a vital role in phagocytosis and in signaling to T cells, facilitating the transition to adaptive immunity. The sustained elevation of active macrophages across the simulation timeline reflects the vaccine’s ability to continuously engage innate defenses and support antigen processing throughout the immune response. Figure 7F shows the response of dendritic cells, highlighting their transition from the immature to the mature state after vaccination. Immature dendritic cells initially dominate but quickly differentiate into mature antigen-presenting cells (APCs) following vaccine exposure. These mature dendritic cells are essential for presenting processed antigens to T cells, thereby initiating a targeted adaptive immune response. The sustained presence of mature dendritic cells, especially after secondary and tertiary exposures, suggests ongoing immune surveillance and readiness to trigger memory responses. This maturation process confirms the vaccine’s effectiveness in mobilizing key components of both the innate and adaptive immune systems for long-term protection. The immune simulation results for HIF-2, HIF-3, HIF-4, and HIF-5 are present as supplementary results (Figs. S2, S3, S4 and S5).

### Molecular docking analysis

**Fig. 8:**
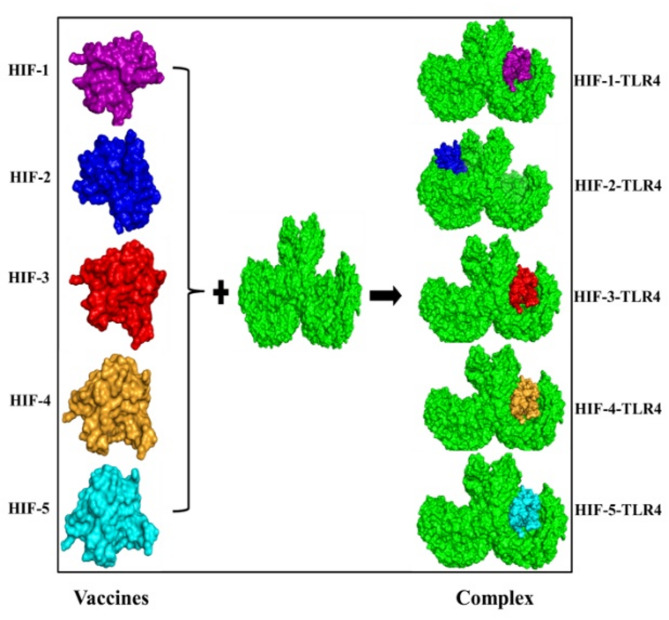
The solid molecular surface representation of the predicted vaccines docked to the TLR-4 receptor.

**Fig. 9:**
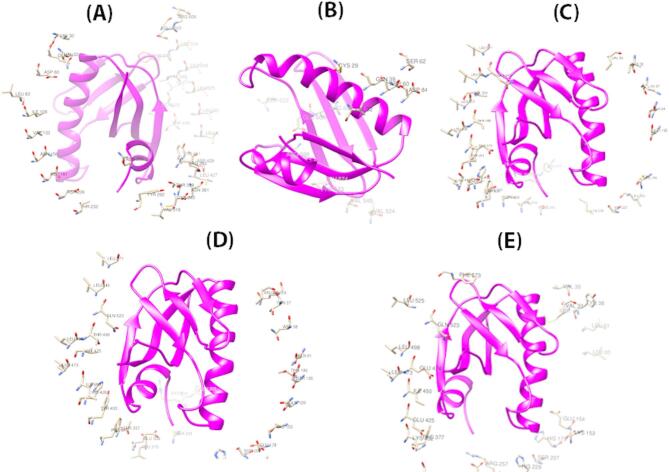
Visualization of the molecular interactions between the HIF-designed vaccine constructs (HIF-1, HIF-2, HIF-3, HIF-4, and HIF-5) and the TLR4 receptor.

To decipher the protein-protein binding pattern and to investigate the role of hydrophobic interactions, the molecular docking between the HIF designed vaccine constructs and TLR4 receptor was carried out using the ClusPro server. The 3D structures of vaccine constructs HIF-1, HIF-2, HIF-3, HIF-4, and HIF-5 and TLR4 are used in molecular docking to highlight the immunological response. Each construct was separately generated 30 3D clusters after being docked with TLR4 one at a time. Only the model that appropriately occupied the receptor and had the lowest energy score was chosen among them, and it was revealed that model number 0.00 as represented in Table 5 met the required criteria and has the best binding position view and surface exposed for all HIF vaccine constructs and was therefore chosen as the best-docked complexes (Fig. 8). The interaction maps of the docked complexes are illustrated in Fig. 9. Additionally, to ensure broader immunological relevance, molecular docking was performed with MHC Class I and Class II molecules, as presented in Supplementary Table S4. The three-dimensional depictions of the docked complexes are provided in the Supplementary Results (Figures S6 and S7). To more precisely corroborate the docking results and gain understanding of the structural stability and dynamics of the HIF vaccine constructs with TLR4 receptor, the top docked complexes were next subjected to 100-ns MD simulations for each vaccine complex.

**Table 5:** Docking results with weigh score (center and lowest energy) and members of top docked complexes for each construct are listed here.

		HIF-1_TLR-4		HIF-2_TLR-4		HIF-3_TLR-4		HIF-4_TLR-4		HIF-5_TLR-4	
Cluster	Representative	Members	W.Score	Members	W.Score	Members	W.Score	Members	W.Score	Members	W.Score
0	Center	82	−689.7	63	−691.5	82	−689.7	82	−689.7	82	−689.7
	L.energy		−840.5		−766.9		−840.5		−840.5		−840.5
1	Center	82	−695.9	43	−628.3	82	−695.9	82	−695.9	82	−695.9
	L.energy		−779.5		−730		−779.5		−779.5		−779.5
2	Center	72	−708.5	43	−703.4	72	−708.5	72	−708.5	72	−708.5
	L.energy		−718.8		−780.3		−718.8		−718.8		−718.8
3	Center	65	−724.3	41	−630.2	65	−724.3	65	−724.3	65	−724.3
	L.energy		−789.3		−711.2		−789.3		−789.3		−789.3
4	Center	61	−719.4	41	−741.2	61	−719.4	61	−719.4	61	−719.4
	L.energy		−789.2		−761.2		−789.2		−789.2		−789.2
5	Center	58	−695.1	34	−614.7	58	−655.1	58	−655.1	58	−655.1
	L.energy		−768.2		−731.9		−768.2		−768.2		−768.2
6	Center	52	−822.1	29	−632.2	52	−822.1	52	−822.1	52	−822.1
	L.energy		−822.1		−701.1		−822.1		−822.1		−822.1
7	Center	46	−654.1	27	−607.3	46	−654.1	46	−654.1	46	−654.1
	L.energy		−795.6		−702.7		−795.6		−795.6		−795.6
8	Center	36	−659.1	27	−686.7	36	−659.1	36	−659.1	36	−659.1
	L.energy		−724.9		−733.2		−724.9		−724.9		−724.9

*W.Score: Weight Score, *L.Energy: Lowest Energy.

### Molecular dynamic simulations

**Fig. 10:**
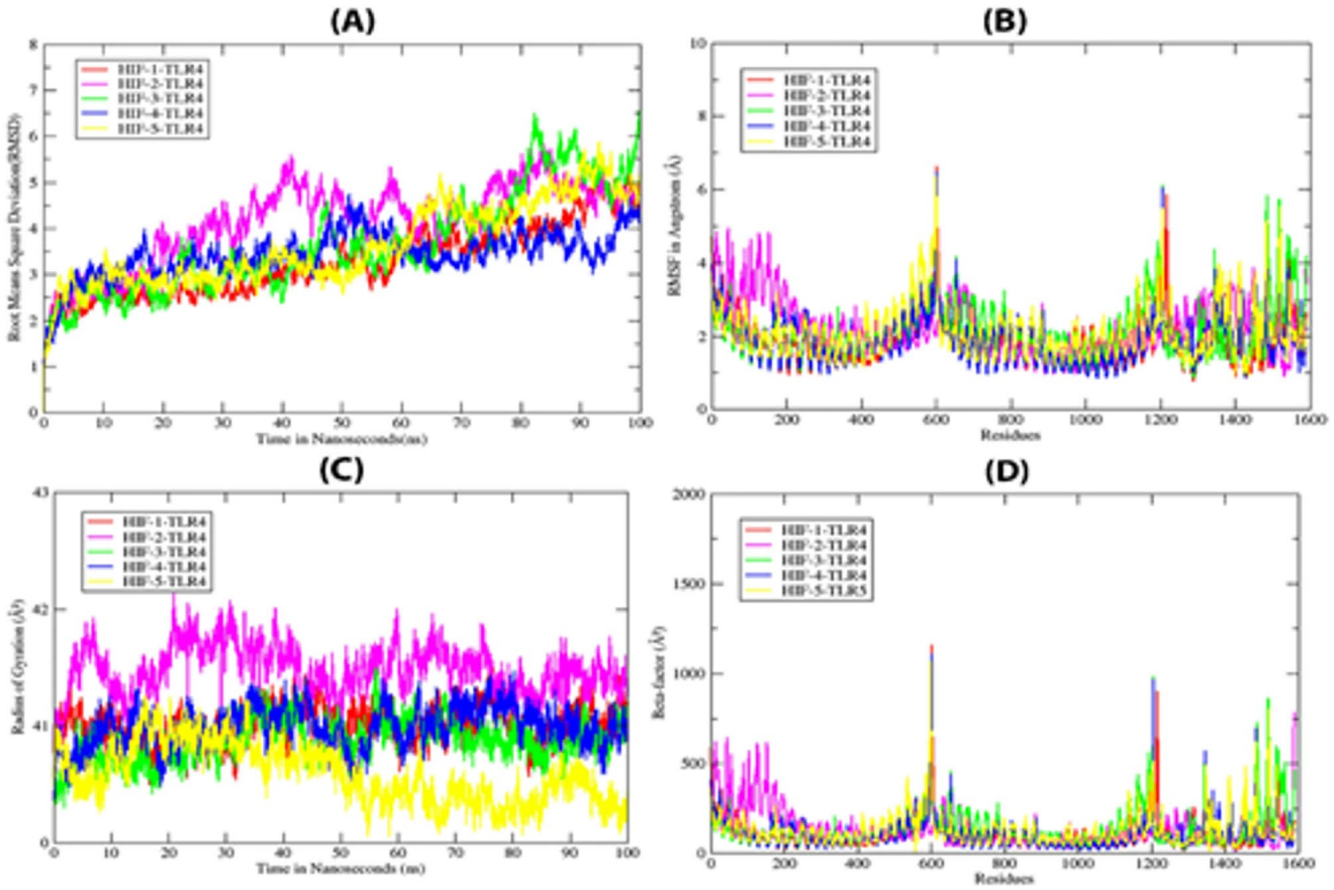
The graphs of Trajectory analysis that are representing the dynamic behaviour of HIF-1_TLR-4, HIF-2_TLR-4, HIF-3_TLR-4, HIF-4_TLR-4 and HIF-5_TLR-4 complexes throughout 500ns simulation. (**A**)RMSD graph, (**B**) RMSF graph, (**C**) Radius of gyration (Rg) graph and Beta-factor graph.

MD simulation demonstrates the mechanism of molecule binding interaction with respect to time. A total 500 ns MD simulation was performed to evaluate the structural stability of the docked HIF-1_TLR-4, HIF-2_TLR-4, HIF-3_TLR-4, HIF-4_TLR-4 and HIF-5_TLR-4 complexes. The observed fluctuation in RMSD graphs for all complexes at different nanoseconds is highlighted in the given graph (Fig. 10). Figure 10A reveals the abrupt dynamic behavior and more fluctuation for the HIF-2_TLR-4 and HIF-3_TLR-4 complexes among all the 5 complexes while HIF-1_TLR-4 and HIF-5_TLR-4 complexes show less fluctuation as compared to HIF-2_TLR-4 and HIF-3_TLR-4. RMSD graph having a less value and less fluctuation reflect a solid interaction of HIF-4 and TLR4.This whole scenario is highlighting the phenomena that HIF-4-TLR4 complex is more stable vaccine construct among all the complexes. The RMSD plot (Fig. 10A) illustrates the maximum and average RMSD values 5.3Å and 3.4Å respectively for HIF-1_TLR-4 docked complex. While the HIF-2_TLR-4, HIF-3_TLR-4, HIF-4_TLR-4 and HIF-5_TLR-4 complexes have a 5.8Å, 6.5Å, 4.7Å, 5.8Å maximum and 4.1Å,3.6Å,3.2Å,3.6Å average values, respectively. Overall, all the variations observed corresponded to a increase in RMSD values due to the orientation of the vaccine-constructs in the TLR4 pocket. RMSF plots showing a function of residue number were plotted to compare structural mobility of Vaccine complex. The RMSF mean values for HIF-1_TLR-4, HIF-2_TLR-4, HIF-3_TLR-4, HIF-4_TLR-4 and HIF-5_TLR-4 are 1.8Å, 2.1Å, 2.1Å, 1.8Å and 2.1Å, respectively (Fig. 10B). The high peaks are observed in some regions as they contain highly fluctuating residues. The fluctuations in RMSF graphs are highlighted the slight structural changes at the relevant residue position, thus the whole phenomena is helping to bind HIF vaccine constructs with TLR4 in best optimal conformation. Radius of gyration is a strategy to reveal the importance of structural compactness and expanding while packing the secondary structure into 3D structure. Rg pattern observation reveals the changes that occur in compactness of protein complex structure. The average Rg values for HIF-1_TLR-4, HIF-2_TLR-4, HIF-3_TLR-4, HIF-4_TLR-4 and HIF-5_TLR-4 complexes are 41.0 Å, 41.8 Å,40.8 Å, 40.5Å and 40.6 Å respectively in given graphs. Greater Rg values suggest loose system packing, while low values indicate compactness of structure. Through Rg plot, it is observed that HIF-4_TLR-4 complex has less Rg value among all complexes (Fig. 10C).

This comparison highlights and suggesting HIF-4_TLR-4 has less tightness and loose packing in 3D structure. So loose packing of 3D structure has more ability of flexibility and accommodation to adopt another molecule. In relation with RMSF, B-factor measures the changes in each system with respect to the temperature dependent vibrant. The B-factor graph is plotted to report the disorder in Ca atoms as a result of vibrations. Average B-factor value for HIF-1_TLR-4, HIF-2_TLR-4, HIF-3_TLR-4, HIF-4_TLR-4 and HIF-5_TLR-4 complexes are 108.0Å, 120.8 Å,133.1 Å, 102.7Å and 123.7 Å respectively (Fig. 10D). B-factor having less value reflects the overall stability of the system.

### Hydrogen bond analysis

**Fig. 11:**
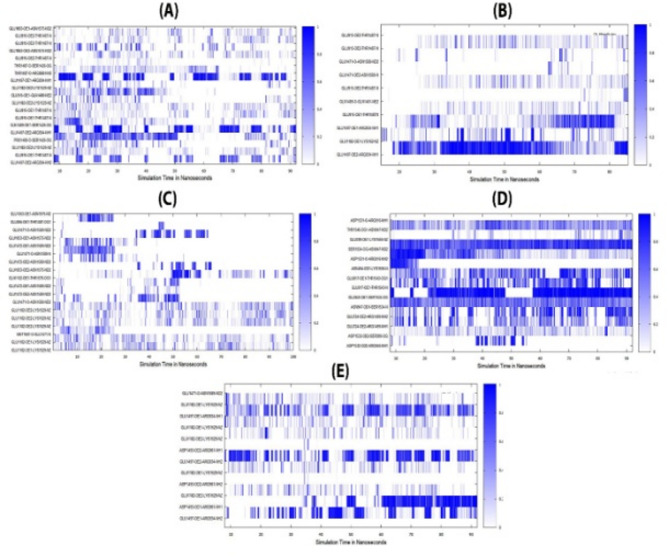
Hydrogen bond Analysis of HIF vaccine constructs and the TLR-4 receptor. (**A**) HIF-1_TLR-4 complex, (**B**) HIF-2_TLR-4 complex, (**C**) HIF-3_TLR-4 complex, (**D**) HIF-4_TLR-4 complex, (**E**) HIF-5_TLR-4 complex.

H-bonds are essential for the molecular recognition and stability of the protein complex structure. The binding and hydrogen bond pattern of the HIF vaccine constructs with the TLR4 receptor is examined and assessed via simulation trajectories. The hydrogen bonding pattern appears more frequently during the simulation, revealing the strong binding of HIF vaccine constructs with TLR4 (Fig. 11). TLR4 residue Glu1497 atoms OE1 and OE2 were spotted breaking hydrogen bonds with NH1 atoms of HIF-1 residue Arg934 on a frequent basis throughout the simulation (Fig. 11A). TLR4 residue Glu1497 atoms OE2 form strong.

hydrogen bonds with NH1 atoms in HIF-2, revealing the complex stable binding (Fig. 11B).

The hydrogen bonds pattern in HIF-3 is not consistent throughout the simulation time because atoms OE1, OE2 of Glu1182 are creating bonds with lys1529 atoms and discovered a gap of missing hydrogen bonds pattern (Fig. 11C). Throughout the trajectory frames, the HIF-4 was found to have the most enriched hydrogen bond network. The results are consistent with the MD predictions of compound stable binding, resulting in strong intermolecular affinity and stable complex formation (Figure D). Lastly, the HIF-5 construct hydrogen bonds pattern is also emphasized, which depicts a consistent hydrogen bond pattern following distinct atom interactions at different simulation time intervals (Fig. 8E).

### Binding free energy landscape

The MM(PB/GB) technique was used to calculate the binding free energy of the TLR-4 vaccine construct complex in order to assess interaction between the constructs. Because of their higher accuracy and computational efficiency compared to molecular docking’s scoring function, these methods are frequently utilized to evaluate binding free energies. The systems under investigation demonstrated very strong and stable binding energies, with net binding energies of HIF-1_TLR-4, HIF-2_TLR-4, HIF-3_TLR-4, HIF-4_TLR-4 and HIF-5_TLR-4 are − 2.1329, −1.5874, 1.0977, −2.8200, 0.8030 kcal/mol in MM-GBSA and − 0.8033, 2.1121, 1.6509, −1.5497, 2.9452 kcal/mol in MMPBSA respectively, as presented in Table 6.

**Table 6:** Binding free energy calculated for all HIF-TLR4 complexes.

	HIF-1_TLR-4		HIF-2_TLR-4		HIF-3_TLR-4		HIF-4_TLR-4		HIF-5_TLR-4	
Energy Components	MM/GBSA (kcalmol)	MM/PBSA (kcalmol)	MM/GBSA (kcalmol)	MM/PBSA (kcalmol)	MM/GBSA (kcalmol)	MM/PBSA (kcalmol)	MM/GBSA (kcalmol)	MM/PBSA (kcalmol)	MM/GBSA (kcalmol)	MM/PBSA (kcalmol)
**VDWAALS**	−4.4407	−4.4407	−3.6027	−3.6027	−7.5363	−7.5363	−7.7445	−7.7445	−9.6016	−9.6016
**EEL**	58.5706	58.5706	46.8012	46.8012	74.2717	74.2717	97.4013	97.4013	118.4779	118.4779
**EGB**	−114.1894	N/A	−89.3558	N/A	−123.0885	N/A	−151.8438	N/A	−167.4512	N/A
**ESURF**	−2.1768	N/A	−0.9265	N/A	−1.8405	N/A	−2.0589	N/A	−1.9922	N/A
**ΔG gas**	114.2333	114.2333	91.8696	91.8696	126.0267	126.0267	151.0827	151.0827	170.2464	170.2464
**ΔG solv**	−116.3661	−113.4299	−90.2822	−89.7575	−124.9290	−124.3758	−153.9027	−152.6324	−169.4434	−167.3012
**EPB**	N/A	−111.9978	N/A	−89.0023	N/A	−122.7923	N/A	−151.1916	N/A	−165.8090
**ENPOLAR**	N/A	−1.4322	N/A	−0.7552	N/A	−1.5834	N/A	−1.4408	N/A	−1.4922
**ΔTotal**	−2.1329	0.8033	−1.5874	2.1121	1.0977	1.6509	−2.8200	−1.5497	0.8030	2.9452

The total binding free energy for HIF-4-TLR4 demonstrating the molecules’ strong interaction affinity. The fact that MMGBSA’s total is higher than MMPBSA’s suggests that it is more able to differentiate between the appropriate binding structurers and the decoys produced by protein-protein docking. HIF-4-TLR4 can be a preferred vaccine construct for immunogenic response in humans because it has been studied and evaluated as the best construct in these analyses: molecular dynamic simulation, immunological simulation/binding free energy calculation, and hydrogen bond analysis. These characteristics of HIF-4 can boost the immune system’s ability to fight off infections and increase immunogenic responses.

## Discussion

*Haemophilus influenza* often adjusts its proteome depending on the environmental conditions to adapt to host immune defense system that persists within various host tissues. Given the proteomic diversity that *H. influenza* exhibits, this adaptability is particularly significant both in expression and its function across its structural proteins like Protein E, PilA, Protein D, P4, TolC, YadA, and HifC. This diversity not only facilitates its survival under multiple environmental stresses but also presents unique challenges in developing broad-spectrum, effective vaccines, particularly for NTHi strains, which exhibit considerable resistance.

Efforts to develop vaccines targeting *H. influenza* antigens began decades ago, focusing on various outer membrane proteins along with virulence factors which contribute to the establishment of infection such as lipopolysaccharide (LPS) antigens, OMP P6, and Protein D^121,122^.These proteins were studied for their role in stimulating immune responses which revealed potential for these antigens to induce protective immunity. However, given the adaptive capacity of *H. influenza*, effective vaccines require a multifaceted approach targeting proteins that are integral to colonization, immune evasion, and persistent within the host.

Our pipeline prioritized outer membrane and secreted proteins as potential vaccine candidates based on their role in pathogenicity, immune system interaction, and conserved expression. This approach leveraged computational analyses to identify surface-exposed and essential proteins with high antigenic potential. For instance, Protein E binds to host components like vitronectin, aiding in immune evasion and attachment to epithelial cells. The role of this protein has been validated by *Murphy et al.*, who found it critical for NTHi colonization and immune escape. Similarly, another protein involved in immune evasion and modulation is YadA, which interacts with extracellular matrix via proteins like collagen and interferes with phagocytosis, thus modulating host immune responses and making it a valuable target in preventing both bacterial attachment and immune modulation^123–126^.

The presence of outer membrane protein P4 which is essential for iron acquisition, binding heme for survival under iron-limiting conditions within the host was identified in the study, which is generally present in about six to eight in number lying at the surface. The commonly utilized outer membrane proteins in previous vaccine studies are P2, P5, and P6^127^, which in this study were identified in R2866 and Rd KW20 strains along with the other NTHi strains. The previous studies emphasized the critical role of OMPs in bacterial virulence and survival, reinforcing its relevance in vaccine formulations aimed at interrupting iron metabolism in pathogens^127,]^. Furthermore, the recently identified vaccine candidate in literature (OMP P6), was also screened in the current analysis, which displays low levels of allelic variation among other outer membrane proteins^129,130^, suggesting OMPs to be a potential vaccine candidate holding low levels of allelic diversity that should be tested in vitro.

Other important virulent factors identified in the current study are hif proteins that are found in both encapsulated and non-encapsulated NTHi strains [96], [97]. HifC, a fimbrial assembly protein, is instrumental in enhancing the ability of *H. influenza* to form fimbriae, which improves adherence to host tissues and biofilm stability. It has also been reported in the previous studies that hif genes colonize more rapidly and therefore have fewer chances of allelic diversity. Similarly, PilA proteins with the potential of participating in quorum sensing leading to biofilm formation, a feature that enables NTHi to evade both immune responses and antibiotics^131^ was independently present in different NTHi strains^132^. *Erwin et al.*, demonstrated that targeting PilA disrupts biofilm formation, thus reducing bacterial survival within the host^133^. Furthermore, *Carine et al.* have proposed a fusion vaccine construct of Protein E and PilA against NTHi influenza strains which successfully reduced signs of Otitis media in mice and chinchillas when given the anti Protein E antibodies^134^. Given its significance, inclusion of PilA in multiepitope vaccine hold the potential to mitigate biofilm associated chronic infections.

Protein D, a glycerophosphodiester phosphodiesterase, which facilitates nutrient acquisition, allowing *H. influenza* to thrive in nutrient scarce environments within the host was observed in strains 5P28H1/5P54H1, 6P24H2/6P32H1, 48P106H1/48P153H1, and 67P38H1/67P56H1 in this study, which belong to the chronic obstructive pulmonary disease (COPD), causing secondary immunodeficiency^101^. Antibody against *H. influenza* Protein D has been previously reported by *Hawdon et al.*, particularly in patients with COPD highlighting its significance as vaccine target^135^.

Last but not the least, TolC, a part of the tripartite efflux system that enables the expulsion of toxic compounds including antibiotics, contributes greatly to multidrug resistance (MDR) has also been a part of the multiepitope vaccine construct predicted in the study. TolC has been predicted and evaluated in previous vaccine design studies for example, the researchers demonstrated the potential of this recombinant protein against *Shigella flexneri* leveraging its conserved and immunogenic properties when tested in mice^136^.

The overlap of predicted vaccine candidates in the study validated by those discussed in the previous vaccine design studies highlights the robustness of the pipeline proposed. The convergence not only reinforces the relevance of the selected targets for H. influenza but also underscores the reliability of the pipeline in identifying these candidates. The pipeline can therefore serve as a versatile framework for the systematic discovery of immunogenic targets in other pathogens, paving the way for development of broadly applicable vaccine strategies. Furthermore, in-silico methods that provide a rational framework for vaccine candidate identification shall undergo experimental validation to bridge the gap between computational predictions and biological systems. H. Influenza can dynamically alter the expression and surface accessibility of proteins like Protein E, PilA, and P4 depending on host conditions, which may affect immune recognition. Discrepancies between predicted and actual immune responses can also arise due to factors including post translational modifications, epitope masking, allelic variations, and immune evasion strategies. Therefore, experimental assays and animal models are critical to ensure the predicted outcome.

## Conclusion

This study provides a comprehensive way to in silico identify and validate vaccine targets against *Haemophilus influenza* based on genomic, phylogenetic and immunological analyses, which has prioritized six proteins – Protein F, YadA, PilA, Protein D, OMP P4 and TolC for multiepitope vaccine design. This analysis will aid in combating the menace of antibiotic resistance while maintaining all the merits and immunological strategies required for the design of a multi-epitope chimeric vaccine construct. The pan-genome and phylogenetic analysis disclosed that 79% of the core genome constituted accessory and unique genes shaping the virulent repertoire of the pathogen while core virulent genes belonged to the clade V-VI of the previously proposed phylogenetic study. Furthermore, the KEGG analysis confirmed the results by displaying the highest number of accessory genes lying in the cell wall biogenesis, cell motility, immune evasion, biofilm formation and antibiotic resistance pathways. Moreover, the results of immune simulations displayed compatibility with actual immune responses giving rise to the memory formation in TH and TC cell populations along with an elevated secondary and tertiary immunoglobins activity. A marked increase in the concentration of TH cell was noticed that resulted in the production of Ig complementing a satisfactory humoral response. Last but not the least, disulfide bond engineering and MD simulations of over 500 ns validated the structural stability and epitope accessibility of these proteins, supporting their robustness as vaccine antigens. These findings provide a solid foundation for advancing these six proteins into experimental validation and can be particularly effective for patients susceptible to *H. influenza* infection.

## Electronic supplementary material

Below is the link to the electronic supplementary material.

Supplementary Material 1

## Data Availability

Data is provided within the manuscript or supplementary information files.

## Declarations

### Author information

These authors contributed equally: Naila Zaman and Kainat Gul.
