# Supplementary material for: Molecular insights into pangenome localization and constructs design for Hemophilus influenza vaccine
Source: Sci Rep. 2025 Jul 1;15:22316. doi: 10.1038/s41598-025-03536-0 (PMC12217137; doi:10.1038/s41598-025-03536-0)
Supplement: Supplementary file 1 — Supplementary Material 1 [file 41598_2025_3536_MOESM1_ESM.docx]

**Supplementary Material**

**Table S1: Complete genomic information of *H. influenzae* strains predicted by BPGA**

| **Genome_no.** | **Organism_name** | **Filename** | **Number_of_proteins** | **Genomic_G+C_%** | **No. of core genes** | **No. of accessory genes** | **No. of unique genes** | **No. of exclusively absent genes** |
| --- | --- | --- | --- | --- | --- | --- | --- | --- |
| 1 | Haemophilus_influenzae | GCA_003184385.1_ASM318438v1_genomic.gbk | 1522 | 37.9 | 645 | 687 | 134 | 136 |
| 2 | Haemophilus_influenzae | GCA_003184405.1_ASM318440v1_genomic.gbk | 1595 | 38.7 | 645 | 842 | 16 | 107 |
| 3 | Haemophilus_influenzae_86_028NP | GCF_000012185.1_ASM1218v1_genomic.gbk | 1793 | 38.3 | 645 | 1104 | 3 | 1 |
| 4 | Haemophilus_influenzae_PittEE | GCF_000016465.1_ASM1646v1_genomic.gbk | 1509 | 38.2 | 645 | 828 | 7 | 41 |
| 5 | Haemophilus_influenzae_PittGG | GCF_000016485.1_ASM1648v1_genomic.gbk | 1546 | 38.2 | 645 | 838 | 32 | 65 |
| 6 | Haemophilus_influenzae_Rd_KW20 | GCF_000027305.1_ASM2730v1_genomic.gbk | 1610 | 38.5 | 645 |  | 4 | 10 |
| 7 | Haemophilus_influenzae_R2866 | GCF_000165525.1_ASM16552v1_genomic.gbk | 1803 | 38.2 | 645 | 935 | 16 | 0 |
| 8 | Haemophilus_influenzae_R2846 | GCF_000165575.1_ASM16557v1_genomic.gbk | 1656 | 38.2 | 645 | 1115 | 6 | 0 |
| 9 | Haemophilus_influenzae_F3031 | GCF_000197875.1_ASM19787v1_genomic.gbk | 1844 | 38.5 | 645 | 972 | 20 | 1 |
| 10 | Haemophilus_influenzae_F3047 | GCF_000200475.1_ASM20047v1_genomic.gbk | 1867 | 38.5 | 645 | 1115 | 40 | 3 |
| 11 | Haemophilus_influenzae_10810 | GCF_000210875.1_ASM21087v1_genomic.gbk | 1851 | 38.3 | 645 | 1102 | 13 | 1 |
| 12 | Haemophilus_influenzae_KR494 | GCF_000465255.1_ASM46525v1_genomic.gbk | 1635 | 38.3 | 645 | 1125 | 1 | 0 |
| 13 | Haemophilus_influenzae_CGSHiCZ412602 | GCF_000698365.1_ASM69836v1_genomic.gbk | 1655 | 38.2 | 645 | 957 | 11 | 0 |
| 14 | Haemophilus_influenzae | GCF_000767075.1_ASM76707v1_genomic.gbk | 1710 | 38.3 | 645 | 975 | 1 | 0 |
| 15 | Haemophilus_influenzae | GCF_000931575.1_ASM93157v1_genomic.gbk | 1702 | 38.3 | 645 | 1019 | 3 | 0 |
| 16 | Haemophilus_influenzae | GCF_000931605.1_ASM93160v1_genomic.gbk | 1681 | 38.3 | 645 | 1007 | 2 | 0 |
| 17 | Haemophilus_influenzae | GCF_000931625.1_ASM93162v1_genomic.gbk | 1758 | 38.4 | 645 | 1000 | 3 | 1 |
| 18 | Haemophilus_influenzae_2019 | GCF_000968335.1_ASM96833v1_genomic.gbk | 1863 | 38.6 | 645 | 1066 | 7 | 0 |
| 19 | Haemophilus_influenzae | GCF_001457655.1_NCTC8143_genomic.gbk | 1761 | 38.4 | 645 | 1129 | 0 | 0 |
| 20 | Haemophilus_influenzae | GCF_001856725.1_ASM185672v1_genomic.gbk | 1652 | 38.2 | 645 | 1061 | 0 | 2 |
| 21 | Haemophilus_influenzae | GCF_002073475.2_ASM207347v2_genomic.gbk | 1676 | 38.2 | 645 | 949 | 2 | 1 |
| 22 | Haemophilus_influenzae | GCF_002966575.1_ASM296657v1_genomic.gbk | 1688 | 38.2 | 645 | 1000 | 0 | 0 |
| 23 | Haemophilus_influenzae | GCF_002966595.1_ASM296659v1_genomic.gbk | 1690 | 38.3 | 645 | 1013 | 0 | 0 |
| 24 | Haemophilus_influenzae | GCF_002966615.1_ASM296661v1_genomic.gbk | 1747 | 38.3 | 645 | 1015 | 0 | 0 |
| 25 | Haemophilus_influenzae | GCF_002966635.1_ASM296663v1_genomic.gbk | 1745 | 38.3 | 645 | 1062 | 1 | 0 |
| 26 | Haemophilus_influenzae | GCF_002966655.1_ASM296665v1_genomic.gbk | 1657 | 38.3 | 645 | 1057 | 0 | 0 |
| 27 | Haemophilus_influenzae | GCF_002966675.1_ASM296667v1_genomic.gbk | 1655 | 38.2 | 645 | 988 | 0 | 0 |
| 28 | Haemophilus_influenzae | GCF_002966695.1_ASM296669v1_genomic.gbk | 1753 | 38.4 | 645 | 985 | 0 | 0 |
| 29 | Haemophilus_influenzae | GCF_002966715.1_ASM296671v1_genomic.gbk | 1753 | 34.2 | 645 | 1074 | 0 | 0 |
| 30 | Haemophilus_influenzae | GCF_002966735.1_ASM296673v1_genomic.gbk | 1643 | 38.3 | 645 | 1074 | 0 | 0 |
| 31 | Haemophilus_influenzae | GCF_003351425.1_ASM335142v1_genomic.gbk | 1600 | 38.3 | 645 | 972 | 9 | 2 |
| 32 | Haemophilus_influenzae | GCF_003351445.1_ASM335144v1_genomic.gbk | 1708 | 38.3 | 645 | 928 | 2 | 2 |
| 33 | Haemophilus_influenzae | GCF_003351465.1_ASM335146v1_genomic.gbk | 1650 | 38.3 | 645 | 1021 | 9 | 0 |
| 34 | Haemophilus_influenzae | GCF_003351585.1_ASM335158v1_genomic.gbk | 1701 | 38.3 | 645 | 980 | 9 | 0 |
| 35 | Haemophilus_influenzae | GCF_003351605.1_ASM335160v1_genomic.gbk | 1635 | 38.4 | 645 | 1019 | 0 | 0 |
| 36 | Haemophilus_influenzae | GCF_003352345.1_ASM335234v1_genomic.gbk | 1731 | 38.3 | 645 | 974 | 0 | 1 |
| 37 | Haemophilus_influenzae | GCF_003352365.1_ASM335236v1_genomic.gbk | 1696 | 38.3 | 645 | 1053 | 0 | 1 |
| 38 | Haemophilus_influenzae | GCF_003352405.1_ASM335240v1_genomic.gbk | 1745 | 38.3 | 645 | 1031 | 5 | 1 |
| 39 | Haemophilus_influenzae | GCF_900475535.1_44310_E01_genomic.gbk | 1916 | 38.3 | 645 | 1061 | 0 | 0 |
| 40 | Haemophilus_influenzae | GCF_900475755.1_45214_B02_genomic.gbk | 1633 | 38.3 | 645 | 1018 | 0 | 0 |
| 41 | Haemophilus_influenzae | GCF_900475995.1_47555_F02_genomic.gbk | 1690 | 38.4 | 645 | 1014 | 0 | 0 |
| 42 | Haemophilus_influenzae | GCF_900478275.1_34211_D02_genomic.gbk | 1743 | 38.3 | 645 | 967 | 3 | 1 |
| 43 | Haemophilus_influenzae | GCF_900478325.1_33962_G01_genomic.gbk | 1774 | 38.4 | 645 | 1162 | 0 | 0 |
| 44 | Haemophilus_influenzae | GCF_900478735.1_33763_D01_genomic.gbk | 1662 | 38.4 | 645 | 1066 | 0 | 0 |
| 45 | Haemophilus_influenzae | GCF_003425605.1_ASM342560v1_genomic.gbk | 1718 | 38.3 | 645 | 1033 | 0 | 0 |
| 46 | Haemophilus_influenzae | GCF_003425625.1_ASM342562v1_genomic.gbk | 1665 | 38.3 | 645 | 1009 | 0 | 0 |
| 47 | Haemophilus_influenzae | GCF_003425645.1_ASM342564v1_genomic.gbk | 1794 | 38.3 | 645 | 1029 | 5 | 1 |
| 48 | Haemophilus_influenzae | GCF_003425715.1_ASM342571v1_genomic.gbk | 1703 | 38.4 | 645 | 991 | 23 | 2 |
| 49 | Haemophilus_influenzae | GCF_003425765.1_ASM342576v1_genomic.gbk | 1686 | 38.4 | 645 | 1093 | 0 | 0 |
| 50 | Haemophilus_influenzae | GCF_003425815.1_ASM342581v1_genomic.gbk | 1693 | 38.4 | 645 | 1016 | 0 | 1 |
| 51 | Haemophilus_influenzae | GCF_003425935.1_ASM342593v1_genomic.gbk | 1692 | 38.4 | 645 | 1013 | 0 | 0 |
| 52 | Haemophilus_influenzae | GCF_003425955.1_ASM342595v1_genomic.gbk | 1756 | 38.3 | 645 | 1020 | 0 | 0 |
| 53 | Haemophilus_influenzae | GCF_900475535.1_44310_E01_genomic.gbk | 1916 | 38.3 | 645 | 1013 | 1 | 0 |
| 54 | Haemophilus_influenzae | GCF_900475755.1_45214_B02_genomic.gbk | 1633 | 38.3 | 645 | 1066 | 70 | 5 |
| 55 | Haemophilus_influenzae | GCF_900475995.1_47555_F02_genomic.gbk | 1690 | 38.3 | 645 | 1152 | 2 | 2 |
| 56 | Haemophilus_influenzae | GCF_900478275.1_34211_D02_genomic.gbk | 1743 | 38.3 | 645 | 967 | 0 | 1 |
| 57 | Haemophilus_influenzae | GCF_900478325.1_33962_G01_genomic.gbk | 1774 | 38.4 | 645 | 1010 | 1 | 1 |
| 58 | Haemophilus_influenzae | GCF_900478735.1_33763_D01_genomic.gbk | 1662 | 38.4 | 645 | 1079 | 9 | 1 |

**Table S2:** The predicted B-cell and T-Cell epitopes of the selected outer membrane proteins

| **TolC family protein** | | | | | | | | | | | | | | | | | | | | | | | | | | | | | | | |
| --- | --- | --- | --- | --- | --- | --- | --- | --- | --- | --- | --- | --- | --- | --- | --- | --- | --- | --- | --- | --- | --- | --- | --- | --- | --- | --- | --- | --- | --- | --- | --- |
| **BCPred** | | | **BepiPred (epitope thresh hold is 0.5)** | | | | | | | **MHC-pred (MHC-1)Allel used HLA-A*1101** | | | | | | | | | | | | **IEDB analysis(MHC-II)Allel used HDRB1*0101** | | | | | | | | | |
| **Position** | **Epitope** | | **Score** | | **Position** | | | | **Epitope** | Amino acid groups | | Predicted logIC50 (M) | | | Predicted IC50 Value (nM)  But pick only those having value less than 100 | | | Confidence of prediction (Max = 1) | | | | Peptide | | | | Percentile rank | | | | Adjusted rank | |
| 386 | LSNLQQTHSYNQRITQYYRN | | 0.971 | | 379-416 | | | | FTQAQSTLSNLQQTHSYNQRITQYYRNRYNAGVSELRE | [HSYNQRITQ](http://www.ddg-pharmfac.net/mhcpred/scripts/MHCPred_scripts/additive.pl) | | 7.463 | | | 34.43 | | | 0.67 | | | | LQQTHSYNQRITQYY | | | | 64.00 | | | | 64.00 | |
|  |  |  |  |  |  |  |  |  |  | [THSYNQRIT](http://www.ddg-pharmfac.net/mhcpred/scripts/MHCPred_scripts/additive.pl) | | 7.414 | | | 38.55 | | | 0.78 | | | | TLSNLQQTHSYNQRI | | | | 55.00 | | | | 55.00 | |
|  |  |  |  |  |  |  |  |  |  | [NLQQTHSYN](http://www.ddg-pharmfac.net/mhcpred/scripts/MHCPred_scripts/additive.pl) | | 7.306 | | | 49.43 | | | 0.78 | | | | LSNLQQTHSYNQRIT | | | | 56.00 | | | | 56.00 | |
|  |  |  |  |  |  |  |  |  |  | [QTHSYNQRI](http://www.ddg-pharmfac.net/mhcpred/scripts/MHCPred_scripts/additive.pl) | | 7.224 | | | 59.70 | | | 0.56 | | | | NLQQTHSYNQRITQY | | | | 64.00 | | | | 64.00 | |
|  |  |  |  |  |  |  |  |  |  | [SYNQRITQY](http://www.ddg-pharmfac.net/mhcpred/scripts/MHCPred_scripts/additive.pl) | | 7.204 | | | 62.52 | | | 0.89 | | | | LQQTHSYNQRITQYY | | | | 64.00 | | | | 64.00 | |
|  |  |  |  |  |  |  |  |  |  | [QRITQYYRN](http://www.ddg-pharmfac.net/mhcpred/scripts/MHCPred_scripts/additive.pl) | | 7.124 | | | 75.16 | | | 0.78 | | | | QRITQYYRNRYNAGV | | | | 35.00 | | | | 35.00 | |
|  |  |  |  |  |  |  |  |  |  | [YNQRITQYY](http://www.ddg-pharmfac.net/mhcpred/scripts/MHCPred_scripts/additive.pl) | | 7.109 | | | 77.80 | | | 0.89 | | | | RITQYYRNRYNAGV | | | | 32.00 | | | | 32.00 | |
|  |  |  |  |  |  |  |  |  |  | [SNLQQTHSY](http://www.ddg-pharmfac.net/mhcpred/scripts/MHCPred_scripts/additive.pl) | | 7.062 | | | 86.70 | | | 1.00 | | | | QSTLSNLQQTHSYNQ | | | | 52.00 | | | | 52.00 | |
|  |  |  |  |  |  |  |  |  |  | [NQRITQYYR](http://www.ddg-pharmfac.net/mhcpred/scripts/MHCPred_scripts/additive.pl) | | 7.3 | | | 50.12 | | | 0.67 | | | | QTHSYNQRITQYYRN | | | | 65.00 | | | | 65.00 | |
| 339 | NWNTVKWNVKISEADYETAR | | 0.878 | | 336-363 | | | | PFLNWNTVKWNVKISEADYETARNYEQ | [VKWNVKISE](http://www.ddg-pharmfac.net/mhcpred/scripts/MHCPred_scripts/additive.pl) | | 7.671 | | | 21.33 | | | 0.67 | | | | VKWNVKISEAD | | | | 54.00 | | | | 308.52 | |
|  |  |  |  |  |  |  |  |  |  | [NWNTVKWNV](http://www.ddg-pharmfac.net/mhcpred/scripts/MHCPred_scripts/additive.pl) | | 7.624 | | | 23.77 | | | 0.56 | | | | NWNTVKWNVKI | | | | 44.00 | | | | 251.39 | |
|  |  |  |  |  |  |  |  |  |  | [TVKWNVKIS](http://www.ddg-pharmfac.net/mhcpred/scripts/MHCPred_scripts/additive.pl) | | 7.594 | | | 25.47 | | | 0.67 | | | | NTVKWNVKISE | | | | 67.00 | | | | 382.80 | |
|  |  |  |  |  |  |  |  |  |  | [NTVKWNVKI](http://www.ddg-pharmfac.net/mhcpred/scripts/MHCPred_scripts/additive.pl) | | 7.481 | | | 33.04 | | | 0.56 | | | | NTVKWNVKISE | | | | 67.00 | | | | 382.80 | |
|  |  |  |  |  |  |  |  |  |  | [WNTVKWNVK](http://www.ddg-pharmfac.net/mhcpred/scripts/MHCPred_scripts/additive.pl) | | 7.309 | | | 49.09 | | | 0.78 | | | | LNWNTVKWNVK | | | | 46.00 | | | | 262.82 | |
|  |  |  |  |  |  |  |  |  |  | [KWNVKISEA](http://www.ddg-pharmfac.net/mhcpred/scripts/MHCPred_scripts/additive.pl) | | 7.308 | | | 49.20 | | | 0.89 | | | | KWNVKISEADY | | | | 61.00 | | | | 348.52 | |
|  |  |  |  |  |  |  |  |  |  | [KISEADYET](http://www.ddg-pharmfac.net/mhcpred/scripts/MHCPred_scripts/additive.pl) | | 7.303 | | | 49.77 | | | 0.78 | | | | KISEADYETAR | | | | 77.00 | | | | 439.93 | |
|  |  |  |  |  |  |  |  |  |  | [VKISEADYE](http://www.ddg-pharmfac.net/mhcpred/scripts/MHCPred_scripts/additive.pl) | | 7.273 | | | 53.33 | | | 0.67 | | | | VKISEADYETA | | | | 76.00 | | | | 434.22 | |
|  |  |  |  |  |  |  |  |  |  | [WNVKISEAD](http://www.ddg-pharmfac.net/mhcpred/scripts/MHCPred_scripts/additive.pl) | | 7.244 | | | 57.02 | | | 0.67 | | | | WNVKISEADYE | | | | 75.00 | | | | 428.50 | |
|  |  |  |  |  |  |  |  |  |  | [SEADYETAR](http://www.ddg-pharmfac.net/mhcpred/scripts/MHCPred_scripts/additive.pl) | | 7.166 | | | 68.23 | | | 0.78 | | | | SEADYETARNY | | | | 91.00 | | | | 519.92 | |
|  |  |  |  |  |  |  |  |  |  | [NVKISEADY](http://www.ddg-pharmfac.net/mhcpred/scripts/MHCPred_scripts/additive.pl) | | 7.124 | | | 75.16 | | | 0.56 | | | | NVKISEADYET | | | | 76.00 | | | | 434.22 | |
|  |  |  |  |  |  |  |  |  |  | [ISEADYETA](http://www.ddg-pharmfac.net/mhcpred/scripts/MHCPred_scripts/additive.pl) | | 7.124 | | | 75.16 | | | 1.00 | | | | ISEADYETARN | | | | 86.00 | | | | 491.35 | |
| 183 | NETIKYYTDIGNIMQTRLVQ | | 0.949 | | 184-192 | | | | ETIKYYTDI(less than 11 ) not possible | [ETIKYYTDI](http://www.ddg-pharmfac.net/mhcpred/scripts/MHCPred_scripts/additive.pl) | | 7.436 | | | 36.64 | | | 0.44 | | | |  | | | |  | | | |  | |
|  |  | |  | | 89-122 | | | | LVGANLVPAFNGSTSSAAQRRVDISTNSAISHKG |  | | | | | | | | | | | |  | | | | | | | | | |
|  |  | |  | | 288-304 | | | | SSAFKNAKATQKSWFPE |  | | | | | | | | | | | |  | | | | | | | | | |
| 312 | SSTASTVGTALHNPVAAGTV | | 0.819 | | 309-326 | | | | ASLSSTASTVGTALHNPV | [STASTVGTA](http://www.ddg-pharmfac.net/mhcpred/scripts/MHCPred_scripts/additive.pl) | | 7.826 | | | 14.93 | | | 0.89 | | | | STASTVGTALH | | | | 40.00 | | | | 228.53 | |
|  |  |  |  |  |  |  |  |  |  | [STVGTALHN](http://www.ddg-pharmfac.net/mhcpred/scripts/MHCPred_scripts/additive.pl) | | 7.489 | | | 32.43 | | | 0.78 | | | | STVGTALHNPV | | | | 41.00 | | | | 234.25 | |
|  |  |  |  |  |  |  |  |  |  | [TVGTALHNP](http://www.ddg-pharmfac.net/mhcpred/scripts/MHCPred_scripts/additive.pl) | | 7.425 | | | 37.58 | | | 0.89 | | | | STVGTALHNPV | | | | 41.00 | | | | 234.25 | |
|  |  |  |  |  |  |  |  |  |  | [SSTASTVGT](http://www.ddg-pharmfac.net/mhcpred/scripts/MHCPred_scripts/additive.pl) | | 7.403 | | | 39.54 | | | 0.89 | | | | SSTASTVGTAL | | | | 36.00 | | | | 205.68 | |
|  |  |  |  |  |  |  |  |  |  | [ASTVGTALH](http://www.ddg-pharmfac.net/mhcpred/scripts/MHCPred_scripts/additive.pl) | | 7.358 | | | 43.85 | | | 0.89 | | | | ASTVGTALHNP | | | | 77.00 | | | | 439.93 | |
|  |  |  |  |  |  |  |  |  |  | [TASTVGTAL](http://www.ddg-pharmfac.net/mhcpred/scripts/MHCPred_scripts/additive.pl) | | 7.244 | | | 57.02 | | | 0.89 | | | | TASTVGTALHN | | | | 54.00 | | | | 308.52 | |
|  |  |  |  |  |  |  |  |  |  | [VGTALHNPV](http://www.ddg-pharmfac.net/mhcpred/scripts/MHCPred_scripts/additive.pl) | | 7.202 | | | 62.81 | | | 0.89 | | | | SSTASTVGTALHNPV | | | | 52.00 | | | | 52.00 | |
| 141 | DAAEWSHKATAEDMESARLS | | 0.852 | | 131-155 | | | | DLWQRLANTVDAAEWSHKATAEDME | [AEWSHKATA](http://www.ddg-pharmfac.net/mhcpred/scripts/MHCPred_scripts/additive.pl) | | 7.479 | | | 33.19 | | | 0.67 | | | | AEWSHKATAED | | | | 70.00 | | | | 399.94 | |
|  |  |  |  |  |  |  |  |  |  | [AAEWSHKAT](http://www.ddg-pharmfac.net/mhcpred/scripts/MHCPred_scripts/additive.pl) | | 7.359 | | | 43.75 | | | 0.78 | | | | AAEWSHKATAE | | | | 83.00 | | | | 474.21 | |
|  |  |  |  |  |  |  |  |  |  | [SHKATAEDM](http://www.ddg-pharmfac.net/mhcpred/scripts/MHCPred_scripts/additive.pl) | | 7.333 | | | 46.45 | | | 0.56 | | | | EWSHKATAEDM | | | | 60.00 | | | | 342.80 | |
|  |  |  |  |  |  |  |  |  |  | [DAAEWSHKA](http://www.ddg-pharmfac.net/mhcpred/scripts/MHCPred_scripts/additive.pl) | | 7.326 | | | 47.21 | | | 0.78 | | | | DAAEWSHKATA | | | | 68.00 | | | | 388.51 | |
|  |  |  |  |  |  |  |  |  |  | [HKATAEDME](http://www.ddg-pharmfac.net/mhcpred/scripts/MHCPred_scripts/additive.pl) | | 7.092 | | | 80.91 | | | 0.56 | | | | WSHKATAEDME | | | | 73.00 | | | | 417.08 | |
|  |  |  |  |  |  |  |  |  |  | [WSHKATAED](http://www.ddg-pharmfac.net/mhcpred/scripts/MHCPred_scripts/additive.pl) | | 7.05 | | | 89.13 | | | 0.56 | | | | WSHKATAEDME | | | | 73.00 | | | | 417.08 | |
|  |  |  |  |  |  |  |  |  |  |  | |  | | |  | | |  | | | |  | | | |  | | | |  | |
|  |  | |  | | 198-236 | | | | TRLVQGVADAASVDQAQQAILTARNNKLNFETQRKTAEQ |  | | | | | | | | | | | |  | | | | | | | | | |
|  |  | |  | | 243-268 | | | | NLKPNEALNITFPHIMNVKTAGVNLN |  | | | | | | | | | | | |  | | | | | | | | | |
| 35 | KQYEEITKQYNVKENWWSLY | | 0.868 | | 22-58 | | | | NIGDSYQASLEDYKQYEEITKQYNVKENWWSLYDDAQ | [EITKQYNVK](http://www.ddg-pharmfac.net/mhcpred/scripts/MHCPred_scripts/additive.pl) | | 7.642 | | | 22.80 | | | 0.78 | | | | EITKQYNVKEN | | | | 64.00 | | | | 365.66 | |
|  |  |  |  |  |  |  |  |  |  | [NVKENWWSL](http://www.ddg-pharmfac.net/mhcpred/scripts/MHCPred_scripts/additive.pl) | | 7.604 | | | 24.89 | | | 0.44 | | | | YNVKENWWSLY | | | | 58.00 | | | | 331.38 | |
|  |  |  |  |  |  |  |  |  |  | [NWWSLYDDA](http://www.ddg-pharmfac.net/mhcpred/scripts/MHCPred_scripts/additive.pl) | | 7.502 | | | 31.48 | | | 0.44 | | | | KENWWSLYDDA | | | | 93.00 | | | | 531.34 | |
|  |  |  |  |  |  |  |  |  |  | [ITKQYNVKE](http://www.ddg-pharmfac.net/mhcpred/scripts/MHCPred_scripts/additive.pl) | | 7.484 | | | 32.81 | | | 0.67 | | | | ITKQYNVKENW | | | | 67.00 | | | | 382.80 | |
|  |  |  |  |  |  |  |  |  |  | [EEITKQYNV](http://www.ddg-pharmfac.net/mhcpred/scripts/MHCPred_scripts/additive.pl) | | 7.459 | | | 34.75 | | | 0.67 | | | | EEITKQYNVKE | | | | 52.00 | | | | 297.10 | |
|  |  |  |  |  |  |  |  |  |  | [KQYNVKENW](http://www.ddg-pharmfac.net/mhcpred/scripts/MHCPred_scripts/additive.pl) | | 7.427 | | | 37.41 | | | 0.78 | | | | KQYNVKENWWS | | | | 58.00 | | | | 331.38 | |
|  |  |  |  |  |  |  |  |  |  | [KQYEEITKQ](http://www.ddg-pharmfac.net/mhcpred/scripts/MHCPred_scripts/additive.pl) | | 7.298 | | | 50.35 | | | 0.78 | | | | KQYEEITKQYN | | | | 76.00 | | | | 434.22 | |
|  |  |  |  |  |  |  |  |  |  | [ENWWSLYDD](http://www.ddg-pharmfac.net/mhcpred/scripts/MHCPred_scripts/additive.pl) | | 7.297 | | | 50.47 | | | 0.44 | | | | ENWWSLYDDAQ | | | | 58.00 | | | | 331.38 | |
|  |  |  |  |  |  |  |  |  |  | [YEEITKQYN](http://www.ddg-pharmfac.net/mhcpred/scripts/MHCPred_scripts/additive.pl) | | 7.294 | | | 50.82 | | | 0.56 | | | | YEEITKQYNVK | | | | 64.00 | | | | 365.66 | |
|  |  |  |  |  |  |  |  |  |  | [TKQYNVKEN](http://www.ddg-pharmfac.net/mhcpred/scripts/MHCPred_scripts/additive.pl) | | 7.283 | | | 52.12 | | | 0.78 | | | | TKQYNVKENWW | | | | 89.00 | | | | 508.49 | |
|  |  |  |  |  |  |  |  |  |  | [VKENWWSLY](http://www.ddg-pharmfac.net/mhcpred/scripts/MHCPred_scripts/additive.pl) | | 7.283 | | | 52.12 | | | 0.67 | | | | VKENWWSLYDD | | | | 93.00 | | | | 531.34 | |
|  |  |  |  |  |  |  |  |  |  | [YNVKENWWS](http://www.ddg-pharmfac.net/mhcpred/scripts/MHCPred_scripts/additive.pl) | | 7.256 | | | 55.46 | | | 0.44 | | | | YNVKENWWSLY | | | | 58.00 | | | | 331.38 | |
|  |  |  |  |  |  |  |  |  |  | [WWSLYDDAQ](http://www.ddg-pharmfac.net/mhcpred/scripts/MHCPred_scripts/additive.pl) | | 7.183 | | | 65.61 | | | 0.44 | | | | ENWWSLYDDAQ | | | | 58.00 | | | | 331.38 | |
|  |  |  |  |  |  |  |  |  |  | [QYEEITKQY](http://www.ddg-pharmfac.net/mhcpred/scripts/MHCPred_scripts/additive.pl) | | 7.05 | | | 89.13 | | | 0.89 | | | | QYEEITKQYNV | | | | 72.00 | | | | 411.36 | |
|  |  |  |  |  |  |  |  |  |  | [KENWWSLYD](http://www.ddg-pharmfac.net/mhcpred/scripts/MHCPred_scripts/additive.pl) | | 7.272 | | | 53.46 | | | 0.56 | | | | KENWWSLYDDA | | | | 93.00 | | | | 531.34 | |
|  |  |  |  |  |  |  |  |  |  |  |  |  |  |  |  |  |  |  |  |  |  |  | | | |  | | | |  | |
| 364 | RITTALNNVDTNYFAFTQAQ | | 0.855 | |  | | | |  |  | | | | | | | | | | | |  | | | | | | | | | |
| **Surface-adhesin protein E precursor** | | | | | | | | | | | | | | | | | | | | | | | | | | | | | | | |
| **BCPred** | | | | | **BepiPred (epitope thresh hold is 0.5)** | | | | | **MHC-pred (MHC-1)Allel used HLA-A*1101** | | | | | | | | | | | **IEDB analysis(MHC-II)Allel used HDRB1*0101** | | | | | | | | | | |
| **Position** | **Epitope** | | **Score** | | **Position** | | **Epitope** | | | Amino acid groups | | Predicted logIC50 (M) | | Predicted IC50 Value (nM) | | | | Confidence of prediction (Max = 1) | | | Peptide | | | | | Percentile rank | | | | Adjusted rank | |
| 114 | EFWGQGLRAAPKKQKKHTLS | | 0.988 | | 96-141 | | ILNCANYHLTQVRTDFYDEFWGQGLRAAPKKQKKHTLSLTPDTTLY | | | [AAPKKQKKH](http://www.ddg-pharmfac.net/mhcpred/scripts/MHCPred_scripts/additive.pl) | | 7.494 | | 32.06 | | | | 0.67 | | | AAPKKQKKHTL | | | | | 76.00 | | | | 434.22 | |
|  |  |  |  |  |  | |  | | | [APKKQKKHT](http://www.ddg-pharmfac.net/mhcpred/scripts/MHCPred_scripts/additive.pl) | | 7.487 | | 32.58 | | | | 0.44 | | | APKKQKKHTLS | | | | | 76.00 | | | | 434.22 | |
|  |  |  |  |  |  |  |  |  |  | [KKQKKHTLS](http://www.ddg-pharmfac.net/mhcpred/scripts/MHCPred_scripts/additive.pl) | | 7.473 | | 33.65 | | | | 0.67 | | | APKKQKKHTLS | | | | | 76.00 | | | | 434.22 | |
|  |  |  |  |  |  |  |  |  |  | [PKKQKKHTL](http://www.ddg-pharmfac.net/mhcpred/scripts/MHCPred_scripts/additive.pl) | | 7.347 | | 44.98 | | | | 0.56 | | | **RAAPKKQKKHT** | | | | |  | | | |  | |
|  |  |  |  |  |  |  |  |  |  | [LRAAPKKQK](http://www.ddg-pharmfac.net/mhcpred/scripts/MHCPred_scripts/additive.pl) | | 7.249 | | 56.36 | | | | 0.89 | | | LRAAPKKQKKH | | | | | 76.00 | | | | 434.22 | |
|  |  |  |  |  |  |  |  |  |  | [RAAPKKQKK](http://www.ddg-pharmfac.net/mhcpred/scripts/MHCPred_scripts/additive.pl) | | 7.249 | | 56.36 | | | | 1.00 | | | RAAPKKQKKHT | | | | | 93.00 | | | | 531.34 | |
|  |  |  |  |  |  |  |  |  |  | [FWGQGLRAA](http://www.ddg-pharmfac.net/mhcpred/scripts/MHCPred_scripts/additive.pl) | | 7.136 | | 73.11 | | | | 0.89 | | | FWGQGLRAAPK | | | | | 15.00 | | | | 85.70 | |
|  |  |  |  |  |  |  |  |  |  | [GQGLRAAPK](http://www.ddg-pharmfac.net/mhcpred/scripts/MHCPred_scripts/additive.pl) | | 7.126 | | 74.82 | | | | 1.00 | | | GQGLRAAPKKQ | | | | | 42.00 | | | | 239.96 | |
|  |  |  |  |  |  |  |  |  |  | [GLRAAPKKQ](http://www.ddg-pharmfac.net/mhcpred/scripts/MHCPred_scripts/additive.pl) | | 7.123 | | 75.34 | | | | 0.78 | | | GLRAAPKKQKK | | | | | 58.00 | | | | 331.38 | |
|  |  |  |  |  |  |  |  |  |  |  | |  | |  | | | |  | | |  | | | | |  | | | |  | |
| 31 | APPTDVRSGYIRLVKNVNYY | | 0.975 | | 18-41 | | AQIQKAEQNDMKLAPPTDVRSGYI | | | [APPTDVRSG](http://www.ddg-pharmfac.net/mhcpred/scripts/MHCPred_scripts/additive.pl) | | 7.527 | | 29.72 | | | | 0.67 | | | APPTDVRSGYI | | | | | 80.00 | | | | 457.07 | |
|  |  |  |  |  |  |  |  |  |  | [PTDVRSGYI](http://www.ddg-pharmfac.net/mhcpred/scripts/MHCPred_scripts/additive.pl) | | 7.322 | | 47.64 | | | | 0.67 | | | **LAPPTDVRSGY** | | | | | 78.00 | | | | 445.64 | |
| 52 | DSESIWVDNQEPQIVHFDAV | | 0.953 | | 56-65 | | IWVDNQEPQI | | | [WVDNQEPQI](http://www.ddg-pharmfac.net/mhcpred/scripts/MHCPred_scripts/additive.pl) | | 7.598 | | 25.23 | | | | 0.67 | | | Not possible (input must be 11 for iedb) | | | | | | | | | | |
|  |  |  |  |  |  |  |  |  |  | [IWVDNQEPQ](http://www.ddg-pharmfac.net/mhcpred/scripts/MHCPred_scripts/additive.pl) | | 7.342 | | 45.50 | | | | 0.78 | | |  |  |  |  |  |  |  |  |  |  |  |
|  |  |  |  |  |  |  |  |  |  | [EPKRYARSV](http://www.ddg-pharmfac.net/mhcpred/scripts/MHCPred_scripts/additive.pl) | | 7.314 | | 48.53 | | | | 0.44 | | |  | | | | |  | | | |  | |
|  |  |  |  |  |  |  |  |  |  | [GLYVYPEPK](http://www.ddg-pharmfac.net/mhcpred/scripts/MHCPred_scripts/additive.pl) | | 7.284 | | 52.00 | | | | 0.78 | | |  | | | | |  | | | |  | |
|  |  |  |  |  |  |  |  |  |  | [YVYPEPKRY](http://www.ddg-pharmfac.net/mhcpred/scripts/MHCPred_scripts/additive.pl) | | 7.017 | | 96.16 | | | | 0.78 | | |  | | | | |  | | | |  | |
|  |  |  |  |  |  |  |  |  |  |  | |  | |  | | | |  | | |  | | | | |  | | | |  | |
| **YadA_anchor domain-containing protein** | | | | | | | | | | | | | | | | | | | | | | | | | | | | | | | |
| **BCPred** | | | | | **BepiPred (epitope thresh hold is 0.5)** | | | | | **MHC-pred (MHC-1)Allel used HLA-A*1101** | | | | | | | | | | | **IEDB analysis(MHC-II)Allel used HDRB1*0101** | | | | | | | | | | |
| **Position** | **Epitope** | | **Score** | | **Position** | | **Epitope** | | | Amino acid groups | | Predicted logIC50 (M) | | Predicted IC50 Value (nM) | | | Confidence of prediction (Max = 1) | | | | Peptide | | | Percentile rank | | | | Adjusted rank | | | |
| 354 | KDGKDGKDGKNGTGGSGSNS | | **1** | | 311-321 | | KDGKDGKDGKN | | | [DGKDGKDGK](http://www.ddg-pharmfac.net/mhcpred/scripts/MHCPred_scripts/additive.pl) | | 7.516 | | 30.48 | | | 0.78 | | | |  | | |  | | | |  | | | |
|  |  |  |  |  |  |  |  |  |  | [KDGKDGKDG](http://www.ddg-pharmfac.net/mhcpred/scripts/MHCPred_scripts/additive.pl) | | 7.085 | | 41.69 | | | 0.56 | | | | KDGKDGKDGKN | | | 93.00 | | | | 531.34 | | | |
|  |  |  |  |  |  |  |  |  |  | [GKDGKDGKN](http://www.ddg-pharmfac.net/mhcpred/scripts/MHCPred_scripts/additive.pl) | | 7.38 | | 41.69 | | | 0.56 | | | |  | | |  | | | |  | | | |
| 184 | GAAGKDGAKGETGAAGKDGK | | 1 | | 5-22 | | AGKDGAKGETGAAGKDGK | | | [GAKGETGAA](http://www.ddg-pharmfac.net/mhcpred/scripts/MHCPred_scripts/additive.pl) | | 7.403 | | 39.54 | | | 0.89 | | | | GAKGETGAAGK | | | 81.00 | | | | 462.78 | | | |
|  |  |  |  |  |  |  |  |  |  | [TGAAGKDGK](http://www.ddg-pharmfac.net/mhcpred/scripts/MHCPred_scripts/additive.pl) | | 7.309 | | 49.09 | | | 1.00 | | | | GETGAAGKDGK | | | 93.00 | | | | 531.34 | | | |
|  |  |  |  |  |  |  |  |  |  | [KGETGAAGK](http://www.ddg-pharmfac.net/mhcpred/scripts/MHCPred_scripts/additive.pl) | | 7.296 | | 50.58 | | | 1.00 | | | | KGETGAAGKDG | | | 86.00 | | | | 491.35 | | | |
|  |  |  |  |  |  |  |  |  |  | [GETGAAGKD](http://www.ddg-pharmfac.net/mhcpred/scripts/MHCPred_scripts/additive.pl) | | 7.245 | | 56.89 | | | 0.78 | | | | GETGAAGKDGK | | | 93.00 | | | | 531.34 | | | |
|  |  |  |  |  |  |  |  |  |  | [GKDGAKGET](http://www.ddg-pharmfac.net/mhcpred/scripts/MHCPred_scripts/additive.pl) | | 7.244 | | 57.02 | | | 0.67 | | | | GKDGAKGETGA | | | 93.00 | | | | 531.34 | | | |
|  |  |  |  |  |  |  |  |  |  | [AGKDGAKGE](http://www.ddg-pharmfac.net/mhcpred/scripts/MHCPred_scripts/additive.pl) | | 7.226 | | 59.43 | | | 0.78 | | | | AGKDGAKGETG | | | 86.00 | | | | 491.35 | | | |
|  |  |  |  |  |  |  |  |  |  | [AKGETGAAG](http://www.ddg-pharmfac.net/mhcpred/scripts/MHCPred_scripts/additive.pl) | | 7.216 | | 60.81 | | | 0.78 | | | | AKGETGAAGKD | | | 83.00 | | | | 474.21 | | | |
|  |  |  |  |  |  |  |  |  |  | [ETGAAGKDG](http://www.ddg-pharmfac.net/mhcpred/scripts/MHCPred_scripts/additive.pl) | | 7.17 | | 67.61 | | | 0.67 | | | | GETGAAGKDGK | | | 93.00 | | | | 531.34 | | | |
|  |  |  |  |  |  |  |  |  |  | [KDGAKGETG](http://www.ddg-pharmfac.net/mhcpred/scripts/MHCPred_scripts/additive.pl) | | 7.029 | | 93.54 | | | 0.78 | | | | KDGAKGETGAA | | | 89.00 | | | | 508.49 | | | |
| 3 | GAAGKDGAKGETGAAGKDGK | | 1 | | 184-203 | | GAAGKDGAKGETGAAGKDGK | | | [GAKGETGAA](http://www.ddg-pharmfac.net/mhcpred/scripts/MHCPred_scripts/additive.pl) | | 7.403 | | 39.54 | | | 0.89 | | | | GAKGETGAAGK | | | 81.00 | | | | 462.78 | | | |
|  |  |  |  |  |  |  |  |  |  | [GAAGKDGAK](http://www.ddg-pharmfac.net/mhcpred/scripts/MHCPred_scripts/additive.pl) | | 7.385 | | 41.21 | | | 0.89 | | | | GAAGKDGAKGE | | | 93.00 | | | | 531.34 | | | |
|  |  |  |  |  |  |  |  |  |  | [TGAAGKDGK](http://www.ddg-pharmfac.net/mhcpred/scripts/MHCPred_scripts/additive.pl) | | 7.309 | | 49.09 | | | 1.00 | | | | GETGAAGKDGK | | | 93.00 | | | | 531.34 | | | |
|  |  |  |  |  |  |  |  |  |  | [KGETGAAGK](http://www.ddg-pharmfac.net/mhcpred/scripts/MHCPred_scripts/additive.pl) | | 7.296 | | 50.58 | | | 1.00 | | | | KGETGAAGKDG | | | 86.00 | | | | 491.35 | | | |
|  |  |  |  |  |  |  |  |  |  | [GETGAAGKD](http://www.ddg-pharmfac.net/mhcpred/scripts/MHCPred_scripts/additive.pl) | | 7.245 | | 56.89 | | | 0.78 | | | | GETGAAGKDGK | | | 93.00 | | | | 531.34 | | | |
|  |  |  |  |  |  |  |  |  |  | [GKDGAKGET](http://www.ddg-pharmfac.net/mhcpred/scripts/MHCPred_scripts/additive.pl) | | 7.244 | | 57.02 | | | 0.67 | | | | GKDGAKGETGA | | | 93.00 | | | | 531.34 | | | |
|  |  |  |  |  |  |  |  |  |  | [AGKDGAKGE](http://www.ddg-pharmfac.net/mhcpred/scripts/MHCPred_scripts/additive.pl) | | 7.226 | | 59.43 | | | 0.78 | | | | AGKDGAKGETG | | | 86.00 | | | | 491.35 | | | |
|  |  |  |  |  |  |  |  |  |  | [AAGKDGAKG](http://www.ddg-pharmfac.net/mhcpred/scripts/MHCPred_scripts/additive.pl) | | 7.218 | | 60.53 | | | 0.78 | | | | AAGKDGAKGET | | | 93.00 | | | | 93.00 | | | |
|  |  |  |  |  |  |  |  |  |  | [AKGETGAAG](http://www.ddg-pharmfac.net/mhcpred/scripts/MHCPred_scripts/additive.pl) | | 7.216 | | 60.81 | | | 0.78 | | | | AKGETGAAGKD | | | 83.00 | | | | 474.21 | | | |
|  |  |  |  |  |  |  |  |  |  | [ETGAAGKDG](http://www.ddg-pharmfac.net/mhcpred/scripts/MHCPred_scripts/additive.pl) | | 7.17 | | 67.61 | | | 0.67 | | | | GETGAAGKDGK | | | 93.00 | | | | 531.34 | | | |
|  |  |  |  |  |  |  |  |  |  | [KDGAKGETG](http://www.ddg-pharmfac.net/mhcpred/scripts/MHCPred_scripts/additive.pl) | | 7.029 | | 93.54 | | | 0.78 | | | | KDGAKGETGAA | | | 89.00 | | | | 508.49 | | | |
| 92 | GKDGEKGEKGETGAAGKDGK | | 1 | | 92-111 | | GKDGEKGEKGETGAAGKDGK | | | [GKDGEKGEK](http://www.ddg-pharmfac.net/mhcpred/scripts/MHCPred_scripts/additive.pl) | | 7.701 | | 19.91 | | | 0.78 | | | | GKDGEKGEKGE | | | 93.00 | | | | 531.34 | | | |
|  |  |  |  |  |  |  |  |  |  | [GEKGETGAA](http://www.ddg-pharmfac.net/mhcpred/scripts/MHCPred_scripts/additive.pl) | | 7.425 | | 37.58 | | | 0.78 | | | | GEKGETGAAGK | | | 84.00 | | | | 479.92 | | | |
|  |  |  |  |  |  |  |  |  |  | [GEKGEKGET](http://www.ddg-pharmfac.net/mhcpred/scripts/MHCPred_scripts/additive.pl) | | 7.38 | | 41.69 | | | 0.67 | | | | GEKGEKGETGA | | | 93.00 | | | | 531.34 | | | |
|  |  |  |  |  |  |  |  |  |  | [TGAAGKDGK](http://www.ddg-pharmfac.net/mhcpred/scripts/MHCPred_scripts/additive.pl) | | 7.309 | | 49.09 | | | 1.00 | | | | GETGAAGKDGK | | | 93.00 | | | | 531.34 | | | |
|  |  |  |  |  |  |  |  |  |  | [KGETGAAGK](http://www.ddg-pharmfac.net/mhcpred/scripts/MHCPred_scripts/additive.pl) | | 7.296 | | 50.58 | | | 1.00 | | | | KGETGAAGKDG | | | 86.00 | | | | 491.35 | | | |
|  |  |  |  |  |  |  |  |  |  | [GETGAAGKD](http://www.ddg-pharmfac.net/mhcpred/scripts/MHCPred_scripts/additive.pl) | | 7.245 | | 56.89 | | | 0.78 | | | | GETGAAGKDGK | | | 93.00 | | | | 531.34 | | | |
|  |  |  |  |  |  |  |  |  |  | [ETGAAGKDG](http://www.ddg-pharmfac.net/mhcpred/scripts/MHCPred_scripts/additive.pl) | | 7.17 | | 67.61 | | | 0.67 | | | | GETGAAGKDGK | | | 93.00 | | | | 531.34 | | | |
|  |  |  |  |  |  |  |  |  |  | [EKGEKGETG](http://www.ddg-pharmfac.net/mhcpred/scripts/MHCPred_scripts/additive.pl) | | 7.109 | | 77.80 | | | 0.67 | | | | EKGEKGETGAA | | | 91.00 | | | | 519.92 | | | |
|  |  |  |  |  |  |  |  |  |  | [EKGETGAAG](http://www.ddg-pharmfac.net/mhcpred/scripts/MHCPred_scripts/additive.pl) | | 7.048 | | 89.54 | | | 0.67 | | | | EKGETGAAGKD | | | 87.00 | | | | 497.06 | | | |
|  |  |  |  |  |  |  |  |  |  | [KDGEKGEKG](http://www.ddg-pharmfac.net/mhcpred/scripts/MHCPred_scripts/additive.pl) | | 7.045 | | 90.16 | | | 0.78 | | | | KDGEKGEKGET | | | 93.00 | | | | 531.34 | | | |
|  |  |  |  |  |  |  |  |  |  | [KGEKGETGA](http://www.ddg-pharmfac.net/mhcpred/scripts/MHCPred_scripts/additive.pl) | | 7.002 | | 99.54 | | | 0.89 | | | | KGEKGETGAAG | | | 73.00 | | | | 417.08 | | | |
| 308 | K**NGKDGKDGKDGKNAVASVT** | | 1 | | 309-328 | | NGKDGKDGKDGKNAVASVTT | | | [GKNAVASVT](http://www.ddg-pharmfac.net/mhcpred/scripts/MHCPred_scripts/additive.pl) | | 7.517 | | 30.41 | | | 0.78 | | | | KDGKNAVASVT | | | 43.00 | | | | 245.67 | | | |
|  |  |  |  |  |  |  |  |  |  | [NGKDGKDGK](http://www.ddg-pharmfac.net/mhcpred/scripts/MHCPred_scripts/additive.pl) | | 7.516 | | 30.48 | | | 0.78 | | | | NGKDGKDGKDG | | | 93.00 | | | | 531.34 | | | |
|  |  |  |  |  |  |  |  |  |  | [DGKDGKDGK](http://www.ddg-pharmfac.net/mhcpred/scripts/MHCPred_scripts/additive.pl) | | 7.516 | | 30.48 | | | 0.78 | | | | DGKDGKDGKNA | | | 93.00 | | | | 531.34 | | | |
|  |  |  |  |  |  |  |  |  |  | [GKDGKDGKD](http://www.ddg-pharmfac.net/mhcpred/scripts/MHCPred_scripts/additive.pl) | | 7.38 | | 41.69 | | | 0.56 | | | | GKDGKDGKDGK | | | 93.00 | | | | 531.34 | | | |
|  |  |  |  |  |  |  |  |  |  | [GKDGKDGKN](http://www.ddg-pharmfac.net/mhcpred/scripts/MHCPred_scripts/additive.pl) | | 7.38 | | 41.69 | | | 0.56 | | | | GKDGKDGKNAV | | | 92.00 | | | | 525.63 | | | |
|  |  |  |  |  |  |  |  |  |  | [GKDGKNAVA](http://www.ddg-pharmfac.net/mhcpred/scripts/MHCPred_scripts/additive.pl) | | 7.291 | | 51.17 | | | 0.78 | | | | GKDGKNAVASV | | | 74.00 | | | | 422.79 | | | |
|  |  |  |  |  |  |  |  |  |  | [KDGKDGKNA](http://www.ddg-pharmfac.net/mhcpred/scripts/MHCPred_scripts/additive.pl) | | 7.273 | | 53.33 | | | 0.78 | | | | KDGKDGKNAVA | | | 79.00 | | | | 451.36 | | | |
|  |  |  |  |  |  |  |  |  |  | [DGKNAVASV](http://www.ddg-pharmfac.net/mhcpred/scripts/MHCPred_scripts/additive.pl) | | 7.16 | | 69.18 | | | 0.67 | | | | KDGKNAVASVT | | | 43.00 | | | | 245.67 | | | |
|  |  |  |  |  |  |  |  |  |  | [KNAVASVTT](http://www.ddg-pharmfac.net/mhcpred/scripts/MHCPred_scripts/additive.pl) | | 7.088 | | 81.66 | | | 0.89 | | | | KDGKNAVASVT | | | 43.00 | | | | 245.67 | | | |
|  |  |  |  |  |  |  |  |  |  | [KDGKDGKDG](http://www.ddg-pharmfac.net/mhcpred/scripts/MHCPred_scripts/additive.pl) | | 7.085 | | 82.22 | | | 0.56 | | | | KDGKDGKDGKN | | | 93.00 | | | | 531.34 | | | |
| 446 | GNTVVNNNGITINNAAPNKT | | 1 | | 446-465 | | GNTVVNNNGITINNAPNKT | | | [VVNNNGITI](http://www.ddg-pharmfac.net/mhcpred/scripts/MHCPred_scripts/additive.pl) | | 8.018 | | 9.59 | | | 0.89 | | | | VVNNNGITINN | | | 45.00 | | | | 257.10 | | | |
|  |  |  |  |  |  |  |  |  |  | [ITINNAPNK](http://www.ddg-pharmfac.net/mhcpred/scripts/MHCPred_scripts/additive.pl) | | 7.815 | | 15.31 | | | 1.00 | | | | ITINNAAPNKT | | | 9.40 | | | | 53.71 | | | |
|  |  |  |  |  |  |  |  |  |  | [NNNGITINN](http://www.ddg-pharmfac.net/mhcpred/scripts/MHCPred_scripts/additive.pl) | | 7.734 | | 18.45 | | | 0.78 | | | | NNNGITINNAA | | | 72.00 | | | | 411.36 | | | |
|  |  |  |  |  |  |  |  |  |  | [TVVNNNGIT](http://www.ddg-pharmfac.net/mhcpred/scripts/MHCPred_scripts/additive.pl) | | 7.651 | | 22.34 | | | 0.89 | | | | TVVNNNGITIN | | | 43.00 | | | | 245.67 | | | |
|  |  |  |  |  |  |  |  |  |  | [TINNAPNKT](http://www.ddg-pharmfac.net/mhcpred/scripts/MHCPred_scripts/additive.pl) | | 7.445 | | 35.89 | | | 0.89 | | | | ITINNAAPNKT | | | 9.40 | | | | 53.71 | | | |
|  |  |  |  |  |  |  |  |  |  | [GITINNAPN](http://www.ddg-pharmfac.net/mhcpred/scripts/MHCPred_scripts/additive.pl) | | 7.418 | | 38.19 | | | 0.78 | | | | GITINNAAPNK | | | 35.00 | | | | 199.97 | | | |
|  |  |  |  |  |  |  |  |  |  | [GNTVVNNNG](http://www.ddg-pharmfac.net/mhcpred/scripts/MHCPred_scripts/additive.pl) | | 7.406 | | 39.26 | | | 0.89 | | | | GNTVVNNNGIT | | | 66.00 | | | | 377.08 | | | |
|  |  |  |  |  |  |  |  |  |  | [NTVVNNNGI](http://www.ddg-pharmfac.net/mhcpred/scripts/MHCPred_scripts/additive.pl) | | 7.348 | | 44.87 | | | 0.78 | | | | NTVVNNNGITI | | | 25.00 | | | | 142.83 | | | |
|  |  |  |  |  |  |  |  |  |  | [NNGITINNA](http://www.ddg-pharmfac.net/mhcpred/scripts/MHCPred_scripts/additive.pl) | | 7.343 | | 45.39 | | | 0.78 | | | | NNGITINNAAP | | | 65.00 | | | | 371.37 | | | |
|  |  |  |  |  |  |  |  |  |  | [VNNNGITIN](http://www.ddg-pharmfac.net/mhcpred/scripts/MHCPred_scripts/additive.pl) | | 7.244 | | 57.02 | | | 0.89 | | | | VNNNGITINNA | | | 72.00 | | | | 411.36 | | | |
|  |  |  |  |  |  |  |  |  |  | [NGITINNAP](http://www.ddg-pharmfac.net/mhcpred/scripts/MHCPred_scripts/additive.pl) | | 7.052 | | 88.72 | | | 0.78 | | | | NGITINNAAPN | | | 23.00 | | | | 131.41 | | | |
|  |  |  |  |  |  |  |  |  |  |  | |  | |  | | |  | | | |  | | |  | | | |  | | | |
|  |  |  |  | |  | | GNTVVNNNGITINNAAPNKT | | |  | | | | | | | | | | |  | | | | | | | | | | |
| 53,234 | KNGKDGKNAVASVTTNNDGT | | 0.998 same score | | 316-333 | | GKDGKNAVASVTTNQDG | | | [GKNAVASVT](http://www.ddg-pharmfac.net/mhcpred/scripts/MHCPred_scripts/additive.pl) | | 7.517 | | 30.41 | | | 0.78 | | | | GKNAVASVTTN | | | 43.00 | | | | 245.67 | | | |
|  |  |  |  |  |  |  |  |  |  | [NAVASVTTN](http://www.ddg-pharmfac.net/mhcpred/scripts/MHCPred_scripts/additive.pl) | | 7.408 | | 39.08 | | | 0.78 | | | | GKNAVASVTTN | | | 43.00 | | | | 245.67 | | | |
|  |  |  |  |  |  |  |  |  |  | [GKDGKNAVA](http://www.ddg-pharmfac.net/mhcpred/scripts/MHCPred_scripts/additive.pl) | | 7.291 | | 51.17 | | | 0.78 | | | | GKDGKNAVASV | | | 74.00 | | | | 422.79 | | | |
|  |  |  |  |  |  |  |  |  |  | [DGKNAVASV](http://www.ddg-pharmfac.net/mhcpred/scripts/MHCPred_scripts/additive.pl) | | 7.16 | | 69.18 | | | 0.67 | | | | DGKNAVASVTT | | | 43.00 | | | | 245.67 | | | |
|  |  |  |  |  |  |  |  |  |  | [KNAVASVTT](http://www.ddg-pharmfac.net/mhcpred/scripts/MHCPred_scripts/additive.pl) | | 7.088 | | 81.66 | | | 0.89 | | | | GKNAVASVTTN | | | 43.00 | | | | 422.79 | | | |
| 571 | SANTNSRGDAGAAASVGYQW | | 0.996 | | 573-580 | | NTNSRGDA | | |  | | | | | | | | | | | Not possible | | | | | | | | | | |
| 30,211,119 | TNEDGTHTINITDGNGAVSS | | 0.983(same for all 3 epitope) | | 251-267 | | DGTHTINITDGNGAVSS | | | [GTHTINITD](http://www.ddg-pharmfac.net/mhcpred/scripts/MHCPred_scripts/additive.pl) | | 8.026 | | 9.42 | | | 0.78 | | | | GTHTINITDGN | | | 91.00 | | | | 519.92 | | | |
|  |  |  |  |  |  |  |  |  |  | [HTINITDGN](http://www.ddg-pharmfac.net/mhcpred/scripts/MHCPred_scripts/additive.pl) | | 7.74 | | 18.20 | | | 0.78 | | | | HTINITDGNGA | | | 62.00 | | | | 354.23 | | | |
|  |  |  |  |  |  |  |  |  |  | [TINITDGNG](http://www.ddg-pharmfac.net/mhcpred/scripts/MHCPred_scripts/additive.pl) | | 7.646 | | 22.59 | | | 0.67 | | | | TINITDGNGAV | | | 51.00 | | | | 291.38 | | | |
|  |  |  |  |  |  |  |  |  |  | [THTINITDG](http://www.ddg-pharmfac.net/mhcpred/scripts/MHCPred_scripts/additive.pl) | | 7.374 | | 42.27 | | | 0.56 | | | | THTINITDGNG | | | 91.00 | | | | 519.92 | | | |
|  |  |  |  |  |  |  |  |  |  | [ITDGNGAVS](http://www.ddg-pharmfac.net/mhcpred/scripts/MHCPred_scripts/additive.pl) | | 7.343 | | 45.39 | | | 0.78 | | | | INITDGNGAVS | | | 49.00 | | | | 279.96 | | | |
|  |  |  |  |  |  |  |  |  |  | [DGTHTINIT](http://www.ddg-pharmfac.net/mhcpred/scripts/MHCPred_scripts/additive.pl) | | 7.208 | | 61.94 | | | 0.78 | | | | DGTHTINITDG | | | 87.00 | | | | 497.06 | | | |
|  |  |  |  |  |  |  |  |  |  | [NITDGNGAV](http://www.ddg-pharmfac.net/mhcpred/scripts/MHCPred_scripts/additive.pl) | | 7.125 | | 74.99 | | | 0.78 | | | | NITDGNGAVSS | | | 48.00 | | | | 274.24 | | | |
|  |  |  |  |  |  |  |  |  |  | [INITDGNGA](http://www.ddg-pharmfac.net/mhcpred/scripts/MHCPred_scripts/additive.pl) | | 7.115 | | 76.74 | | | 1.00 | | | | INITDGNGAVS | | | 49.00 | | | | 279.96 | | | |
|  |  |  |  |  |  |  |  |  |  | [TDGNGAVSS](http://www.ddg-pharmfac.net/mhcpred/scripts/MHCPred_scripts/additive.pl) | | 7.022 | | 95.06 | | | 0.78 | | | | NITDGNGAVSS | | | 48.00 | | | | 274.24 | | | |
| 268 | AIVKNGKDGKNAVANITENN | | 0.981 | | 268-287 | | AIVKNGKDGKNAVANITENN | | | [AVANITENN](http://www.ddg-pharmfac.net/mhcpred/scripts/MHCPred_scripts/additive.pl) | | 7.806 | | 15.63 | | | 0.89 | | | | GKNAVANITEN | | | 68.00 | | | | 388.51 | | | |
|  |  |  |  |  |  |  |  |  |  | [IVKNGKDGK](http://www.ddg-pharmfac.net/mhcpred/scripts/MHCPred_scripts/additive.pl) | | 7.794 | | 16.07 | | | 0.89 | | | | IVKNGKDGKNA | | | 85.00 | | | | 485.64 | | | |
|  |  |  |  |  |  |  |  |  |  | [AIVKNGKDG](http://www.ddg-pharmfac.net/mhcpred/scripts/MHCPred_scripts/additive.pl) | | 7.552 | | 28.05 | | | 0.67 | | | | AIVKNGKDGKN | | | 82.00 | | | | 468.50 | | | |
|  |  |  |  |  |  |  |  |  |  | [GKNAVANIT](http://www.ddg-pharmfac.net/mhcpred/scripts/MHCPred_scripts/additive.pl) | | 7.393 | | 40.46 | | | 0.78 | | | | GKNAVANITEN | | | 68.00 | | | | 388.51 | | | |
|  |  |  |  |  |  |  |  |  |  | [DGKNAVANI](http://www.ddg-pharmfac.net/mhcpred/scripts/MHCPred_scripts/additive.pl) | | 7.322 | | 47.64 | | | 0.67 | | | | DGKNAVANITE | | | 63.00 | | | | 359.94 | | | |
|  |  |  |  |  |  |  |  |  |  | [VKNGKDGKN](http://www.ddg-pharmfac.net/mhcpred/scripts/MHCPred_scripts/additive.pl) | | 7.318 | | 48.08 | | | 0.67 | | | | VKNGKDGKNAV | | | 84.00 | | | | 479.92 | | | |
|  |  |  |  |  |  |  |  |  |  | [GKDGKNAVA](http://www.ddg-pharmfac.net/mhcpred/scripts/MHCPred_scripts/additive.pl) | | 7.291 | | 51.17 | | | 0.78 | | | | GKDGKNAVANI | | | 86.00 | | | | 491.35 | | | |
|  |  |  |  |  |  |  |  |  |  | [NAVANITEN](http://www.ddg-pharmfac.net/mhcpred/scripts/MHCPred_scripts/additive.pl) | | 7.273 | | 53.33 | | | 0.78 | | | | KNAVANITENN | | | 83.00 | | | | 474.21 | | | |
|  |  |  |  |  |  |  |  |  |  | [KNGKDGKNA](http://www.ddg-pharmfac.net/mhcpred/scripts/MHCPred_scripts/additive.pl) | | 7.237 | | 57.94 | | | 0.89 | | | | KNGKDGKNAVA | | | 79.00 | | | | 451.36 | | | |
|  |  |  |  |  |  |  |  |  |  | [KNAVANITE](http://www.ddg-pharmfac.net/mhcpred/scripts/MHCPred_scripts/additive.pl) | | 7.15 | | 70.79 | | | 0.89 | | | | KNAVANITENN | | | 83.00 | | | | 474.21 | | | |
| 142 | KNGKDGKNAVANITENNDGS | | 0.972 | | 273-291 | | GKDGKNAVANITENNDGS | | | [AVANITENN](http://www.ddg-pharmfac.net/mhcpred/scripts/MHCPred_scripts/additive.pl) | | 7.806 | | 15.63 | | | 0.89 | | | | AVANITENNDG | | | 86.00 | | | | 491.35 | | | |
|  |  |  |  |  |  |  |  |  |  | [NITENNDGS](http://www.ddg-pharmfac.net/mhcpred/scripts/MHCPred_scripts/additive.pl) | | 7.461 | | 34.59 | | | 0.78 | | | | VANITENNDGS | | | 80.00 | | | | 457.07 | | | |
|  |  |  |  |  |  |  |  |  |  | [VANITENND](http://www.ddg-pharmfac.net/mhcpred/scripts/MHCPred_scripts/additive.pl) | | 7.415 | | 38.46 | | | 0.78 | | | | VANITENNDGS | | | 80.00 | | | | 457.07 | | | |
|  |  |  |  |  |  |  |  |  |  | [GKNAVANIT](http://www.ddg-pharmfac.net/mhcpred/scripts/MHCPred_scripts/additive.pl) | | 7.393 | | 40.46 | | | 0.78 | | | | GKNAVANITEN | | | 68.00 | | | | 388.51 | | | |
|  |  |  |  |  |  |  |  |  |  | [ANITENNDG](http://www.ddg-pharmfac.net/mhcpred/scripts/MHCPred_scripts/additive.pl) | | 7.344 | | 45.29 | | | 0.78 | | | | AVANITENNDG | | | 86.00 | | | | 491.35 | | | |
|  |  |  |  |  |  |  |  |  |  | [DGKNAVANI](http://www.ddg-pharmfac.net/mhcpred/scripts/MHCPred_scripts/additive.pl) | | 7.322 | | 47.64 | | | 0.67 | | | | DGKNAVANITE | | | 63.00 | | | | 359.94 | | | |
|  |  |  |  |  |  |  |  |  |  | [GKDGKNAVA](http://www.ddg-pharmfac.net/mhcpred/scripts/MHCPred_scripts/additive.pl) | | 7.291 | | 51.17 | | | 0.78 | | | | GKDGKNAVANI | | | 86.00 | | | | 491.35 | | | |
|  |  |  |  |  |  |  |  |  |  | [NAVANITEN](http://www.ddg-pharmfac.net/mhcpred/scripts/MHCPred_scripts/additive.pl) | | 7.273 | | 53.33 | | | 0.78 | | | | NAVANITENND | | | 79.00 | | | | 451.36 | | | |
|  |  |  |  |  |  |  |  |  |  | [KNAVANITE](http://www.ddg-pharmfac.net/mhcpred/scripts/MHCPred_scripts/additive.pl) | | 7.15 | | 70.79 | | | 0.89 | | | | KNAVANITENN | | | 83.00 | | | | 474.21 | | | |
| 468 | VTENGLNNGGNRIINVAPGI | | 0.968 | | 468-487 | | VTENGLNNGGNRIINVAPGI | | | [GGNRIINVA](http://www.ddg-pharmfac.net/mhcpred/scripts/MHCPred_scripts/additive.pl) | | 7.605 | | 24.83 | | | 1.00 | | | | GGNRIINVAPG | | | 65.00 | | | | 371.37 | | | |
|  |  |  |  |  |  |  |  |  |  | [VTENGLNNG](http://www.ddg-pharmfac.net/mhcpred/scripts/MHCPred_scripts/additive.pl) | | 7.525 | | 29.85 | | | 0.89 | | | | VTENGLNNGGN | | | 76.00 | | | | 434.22 | | | |
|  |  |  |  |  |  |  |  |  |  | [NGGNRIINV](http://www.ddg-pharmfac.net/mhcpred/scripts/MHCPred_scripts/additive.pl) | | 7.375 | | 42.17 | | | 0.78 | | | | NGGNRIINVAP | | | 76.00 | | | | 434.22 | | | |
|  |  |  |  |  |  |  |  |  |  | [NGLNNGGNR](http://www.ddg-pharmfac.net/mhcpred/scripts/MHCPred_scripts/additive.pl) | | 7.299 | | 50.23 | | | 0.89 | | | | NGLNNGGNRII | | | 30.00 | | | | 171.40 | | | |
|  |  |  |  |  |  |  |  |  |  | [TENGLNNGG](http://www.ddg-pharmfac.net/mhcpred/scripts/MHCPred_scripts/additive.pl) | | 7.297 | | 50.47 | | | 0.78 | | | | TENGLNNGGNR | | | 87.00 | | | | 497.06 | | | |
|  |  |  |  |  |  |  |  |  |  | [GNRIINVAP](http://www.ddg-pharmfac.net/mhcpred/scripts/MHCPred_scripts/additive.pl) | | 7.25 | | 56.23 | | | 0.67 | | | | GNRIINVAPGI | | | 35.00 | | | | 199.97 | | | |
|  |  |  |  |  |  |  |  |  |  | [NRIINVAPG](http://www.ddg-pharmfac.net/mhcpred/scripts/MHCPred_scripts/additive.pl) | | 7.244 | | 57.02 | | | 0.56 | | | | GGNRIINVAPG | | | 65.00 | | | | 65.00 | | | |
|  |  |  |  |  |  |  |  |  |  | [RIINVAPGI](http://www.ddg-pharmfac.net/mhcpred/scripts/MHCPred_scripts/additive.pl) | | 7.205 | | 62.37 | | | 0.89 | | | | GNRIINVAPGI | | | 35.00 | | | | 199.97 | | | |
|  |  |  |  |  |  |  |  |  |  | [NNGGNRIIN](http://www.ddg-pharmfac.net/mhcpred/scripts/MHCPred_scripts/additive.pl) | | 7.131 | | 73.96 | | | 0.78 | | | | NNGGNRIINVA | | | 86.00 | | | | 491.35 | | | |
|  |  |  |  |  |  |  |  |  |  | [ENGLNNGGN](http://www.ddg-pharmfac.net/mhcpred/scripts/MHCPred_scripts/additive.pl) | | 7.042 | | 90.78 | | | 0.78 | | | | VTENGLNNGGN | | | 76.00 | | | | 434.22 | | | |
|  |  |  |  |  |  |  |  |  |  | [GLNNGGNRI](http://www.ddg-pharmfac.net/mhcpred/scripts/MHCPred_scripts/additive.pl) | | 7.036 | | 92.04 | | | 0.89 | | | | GLNNGGNRIIN | | | 27.00 | | | | 154.26 | | | |
| 375 | DGLKFTGNNEVVNNNKLNSK | | 0.916 | | 375-394 | | DGLKFTGNNEVVNNNKLNSK | | | [EVVNNNKLN](http://www.ddg-pharmfac.net/mhcpred/scripts/MHCPred_scripts/additive.pl) | | 7.889 | | 12.91 | | | 0.78 | | | | EVVNNNKLNSK | | | 76.00 | | | | 434.22 | | | |
|  |  |  |  |  |  |  |  |  |  | [GNNEVVNNN](http://www.ddg-pharmfac.net/mhcpred/scripts/MHCPred_scripts/additive.pl) | | 7.733 | | 18.49 | | | 0.89 | | | | GNNEVVNNNKL | | | 58.00 | | | | 331.38 | | | |
|  |  |  |  |  |  |  |  |  |  | [VVNNNKLNS](http://www.ddg-pharmfac.net/mhcpred/scripts/MHCPred_scripts/additive.pl) | | 7.689 | | 20.46 | | | 0.89 | | | | EVVNNNKLNSK | | | 76.00 | | | | 434.22 | | | |
|  |  |  |  |  |  |  |  |  |  | [VNNNKLNSK](http://www.ddg-pharmfac.net/mhcpred/scripts/MHCPred_scripts/additive.pl) | | 7.605 | | 24.83 | | | 1.00 | | | | EVVNNNKLNSK | | | 76.00 | | | | 434.22 | | | |
|  |  |  |  |  |  |  |  |  |  | [TGNNEVVNN](http://www.ddg-pharmfac.net/mhcpred/scripts/MHCPred_scripts/additive.pl) | | 7.57 | | 26.92 | | | 0.89 | | | | KFTGNNEVVNN | | | 54.00 | | | | 308.52 | | | |
|  |  |  |  |  |  |  |  |  |  | [NNEVVNNNK](http://www.ddg-pharmfac.net/mhcpred/scripts/MHCPred_scripts/additive.pl) | | 7.534 | | 29.24 | | | 0.89 | | | | NNEVVNNNKLN | | | 69.00 | | | | 394.22 | | | |
|  |  |  |  |  |  |  |  |  |  | [FTGNNEVVN](http://www.ddg-pharmfac.net/mhcpred/scripts/MHCPred_scripts/additive.pl) | | 7.435 | | 36.73 | | | 0.89 | | | | FTGNNEVVNNN | | | 66.00 | | | | 377.08 | | | |
|  |  |  |  |  |  |  |  |  |  | [GLKFTGNNE](http://www.ddg-pharmfac.net/mhcpred/scripts/MHCPred_scripts/additive.pl) | | 7.34 | | 45.71 | | | 0.78 | | | | GLKFTGNNEVV | | | 30.00 | | | | 171.40 | | | |
|  |  |  |  |  |  |  |  |  |  | [DGLKFTGNN](http://www.ddg-pharmfac.net/mhcpred/scripts/MHCPred_scripts/additive.pl) | | 7.25 | | 56.23 | | | 0.67 | | | | DGLKFTGNNEV | | | 30.00 | | | | 171.40 | | | |
|  |  |  |  |  |  |  |  |  |  | [NEVVNNNKL](http://www.ddg-pharmfac.net/mhcpred/scripts/MHCPred_scripts/additive.pl) | | 7.178 | | 66.37 | | | 0.67 | | | | NEVVNNNKLNS | | | 49.00 | | | | 279.96 | | | |
|  |  |  |  |  |  |  |  |  |  | [KFTGNNEVV](http://www.ddg-pharmfac.net/mhcpred/scripts/MHCPred_scripts/additive.pl) | | 7.135 | | 73.28 | | | 0.78 | | | | KFTGNNEVVNN | | | 54.00 | | | | 308.52 | | | |
|  |  |  |  |  |  |  |  |  |  |  | |  | |  | | |  | | | |  | | |  | | | |  | | | |
| **PilA** | | | | | | | | | | | | | | | | | | | | | | | | | | | | | | | |
| **BCPred** | | | | | **BepiPred (epitope thresh hold is 0.5)** | | | | | **MHC-pred (MHC-1)Allel used HLA-A*1101** | | | | | | | | | | | | **IEDB analysis(MHC-II)Allel used HDRB1*0101** | | | | | | | | | |
| **Position** | **Epitope** | | **Score** | | **Position** | | **Epitope** | | | Amino acid groups | | Predicted logIC50 (M) | | Predicted IC50 Value (nM) | | | | | Confidence of prediction (Max = 1) | | | Peptide | | | | Percentile rank | | | | Adjusted rank | |
| 149 | TDFTSENHNGAGADPVATNK | | 0.99 | | 136-182 | | NGTAEIKVVGKTTTDFTSENHNGAGADPVATNKHISSLTPLNNQNSI | | | [GADPVATNK](http://www.ddg-pharmfac.net/mhcpred/scripts/MHCPred_scripts/additive.pl) | | 7.765 | | 17.18 | | | | | 0.89 | | | GAGADPVATNK | | | | 81.00 | | | | 462.78 | |
|  |  |  |  |  |  | |  | | | [FTSENHNGA](http://www.ddg-pharmfac.net/mhcpred/scripts/MHCPred_scripts/additive.pl) | | 7.699 | | 20.00 | | | | | 1.00 | | | FTSENHNGAGA | | | | 41.00 | | | | 234.25 | |
|  |  |  |  |  |  |  |  |  |  | [SENHNGAGA](http://www.ddg-pharmfac.net/mhcpred/scripts/MHCPred_scripts/additive.pl) | | 7.572 | | 26.79 | | | | | 0.89 | | | SENHNGAGADP | | | | 91.00 | | | | 519.92 | |
|  |  |  |  |  |  |  |  |  |  | [AGADPVATN](http://www.ddg-pharmfac.net/mhcpred/scripts/MHCPred_scripts/additive.pl) | | 7.518 | | 30.34 | | | | | 0.89 | | | GAGADPVATNK | | | | 81.00 | | | | 462.78 | |
|  |  |  |  |  |  |  |  |  |  | [TDFTSENHN](http://www.ddg-pharmfac.net/mhcpred/scripts/MHCPred_scripts/additive.pl) | | 7.474 | | 33.57 | | | | | 0.67 | | | TDFTSENHNGA | | | | 40.00 | | | | 228.53 | |
|  |  |  |  |  |  |  |  |  |  | [DFTSENHNG](http://www.ddg-pharmfac.net/mhcpred/scripts/MHCPred_scripts/additive.pl) | | 7.307 | | 49.32 | | | | | 0.56 | | | DFTSENHNGAG | | | | 44.00 | | | | 251.39 | |
|  |  |  |  |  |  |  |  |  |  | [NHNGAGADP](http://www.ddg-pharmfac.net/mhcpred/scripts/MHCPred_scripts/additive.pl) | | 7.112 | | 77.27 | | | | | 0.56 | | | NHNGAGADPVA | | | | 76.00 | | | | 434.22 | |
|  |  |  |  |  |  |  |  |  |  | [GAGADPVAT](http://www.ddg-pharmfac.net/mhcpred/scripts/MHCPred_scripts/additive.pl) | | 7.1 | | 79.43 | | | | | 0.89 | | | GAGADPVATNK | | | | 81.00 | | | | 462.78 | |
|  |  |  |  |  |  |  |  |  |  | [ENHNGAGAD](http://www.ddg-pharmfac.net/mhcpred/scripts/MHCPred_scripts/additive.pl) | | 7.011 | | 97.50 | | | | | 0.67 | | | ENHNGAGADPV | | | | 57.00 | | | | 325.66 | |
| 74 | TITLENCNVTTTNNKPKATK | | 0.999 | | 880-92 | | CNVTTTNNKPKA | | | [VTTTNNKPK](http://www.ddg-pharmfac.net/mhcpred/scripts/MHCPred_scripts/additive.pl) | | 7.833 | | 14.69 | | | | | 1.00 | | | CNVTTTNNKPKA | | | | 71.00 | | | | 405.65 | |
|  |  |  |  |  |  |  |  |  |  | [CNVTTTNNK](http://www.ddg-pharmfac.net/mhcpred/scripts/MHCPred_scripts/additive.pl) | | 7.831 | | 14.76 | | | | | 0.89 | | |  |  |  |  |  |  |  |  |  |  |
|  |  |  |  |  |  |  |  |  |  | [TTTNNKPKA](http://www.ddg-pharmfac.net/mhcpred/scripts/MHCPred_scripts/additive.pl) | | 7.666 | | 21.58 | | | | | 1.00 | | | NVTTTNNKPKA | | | | 79.00 | | | | 451.36 | |
|  |  |  |  |  |  |  |  |  |  | [NVTTTNNKP](http://www.ddg-pharmfac.net/mhcpred/scripts/MHCPred_scripts/additive.pl) | | 7.543 | | 28.64 | | | | | 0.78 | | |  |  |  |  |  |  |  |  |  |  |
| 172 | SLTPLNNQNSINLHYIAQYY | | 0.735 | |  | |  | | |  | | | | | | | | | | | |  | | | | | | | | | |
| 33 | FGKVVENTCQVNQDSEYECN | | 0.807 | |  | |  | | |  | | | | | | | | | | | |  | | | | | | | | | |
| 110 | YTLKNIKENTGTNDSANKVN | | 0.946 | | 99-125 | | YSWEIADKDNKYTLKNIKENTGTNDSAN | | | [NIKENTGTN](http://www.ddg-pharmfac.net/mhcpred/scripts/MHCPred_scripts/additive.pl) | | 7.637 | | 23.07 | | | | | 0.67 | | | NIKENTGTNDS | | | | 79.00 | | | | 451.36 | |
|  |  |  |  |  |  |  |  |  |  | [YTLKNIKEN](http://www.ddg-pharmfac.net/mhcpred/scripts/MHCPred_scripts/additive.pl) | | 7.448 | | 35.65 | | | | | 0.67 | | | YTLKNIKENTG | | | | 65.00 | | | | 371.37 | |
|  |  |  |  |  |  |  |  |  |  | [NTGTNDSAN](http://www.ddg-pharmfac.net/mhcpred/scripts/MHCPred_scripts/additive.pl) | | 7.41 | | 38.90 | | | | | 0.67 | | | KENTGTNDSAN | | | | 93.00 | | | | 531.34 | |
|  |  |  |  |  |  |  |  |  |  | [IKENTGTND](http://www.ddg-pharmfac.net/mhcpred/scripts/MHCPred_scripts/additive.pl) | | 7.361 | | 43.55 | | | | | 0.78 | | | IKENTGTNDSA | | | | 76.00 | | | | 434.22 | |
|  |  |  |  |  |  |  |  |  |  | [ENTGTNDSA](http://www.ddg-pharmfac.net/mhcpred/scripts/MHCPred_scripts/additive.pl) | | 7.307 | | 49.32 | | | | | 0.89 | | | KENTGTNDSAN | | | | 93.00 | | | | 531.34 | |
|  |  |  |  |  |  |  |  |  |  | [TLKNIKENT](http://www.ddg-pharmfac.net/mhcpred/scripts/MHCPred_scripts/additive.pl) | | 7.293 | | 50.93 | | | | | 0.78 | | | TLKNIKENTGT | | | | 71.00 | | | | 405.65 | |
|  |  |  |  |  |  |  |  |  |  | [KENTGTNDS](http://www.ddg-pharmfac.net/mhcpred/scripts/MHCPred_scripts/additive.pl) | | 7.286 | | 51.76 | | | | | 0.67 | | | KENTGTNDSAN | | | | 93.00 | | | | 531.34 | |
|  |  |  |  |  |  |  |  |  |  | [KNIKENTGT](http://www.ddg-pharmfac.net/mhcpred/scripts/MHCPred_scripts/additive.pl) | | 7.126 | | 74.82 | | | | | 0.78 | | | KNIKENTGTND | | | | 71.00 | | | | 405.65 | |
| **Prepilin peptidase-dependent protein D** | | | | | | | | | | | | | | | | | | | | | | | | | | | | | | | |
| **BCPred** | | | | | **BepiPred (epitope thresh hold is 0.5)** | | | | | **MHC-pred (MHC-1)Allel used HLA-A*1101** | | | | | | | | | | | **IEDB analysis(MHC-II)Allel used HDRB1*0101** | | | | | | | | | | |
| **Position** | **Epitope** | | **Score** | | **Position** | | **Epitope** | | | Amino acid groups | | Predicted logIC50 (M) | | Predicted IC50 Value (nM) | | | | Confidence of prediction (Max = 1) | | | Peptide | | | | Percentile rank | | | | Adjusted rank | | |
| 61 | LCVYSTNETTSCTGGKNGIA | | 0.998 | | 63-99 | | VYSTNETTSCTGGKNGIAADIKTAKGYVASVITQSGG | | | [STNETTSCT](http://www.ddg-pharmfac.net/mhcpred/scripts/MHCPred_scripts/additive.pl) | | 7.916 | | 12.13 | | | | 0.89 | | | VYSTNETTSCT | | | | 51.00 | | | | 291.38 | | |
|  |  |  |  |  |  | |  | | | [ETTSCTGGK](http://www.ddg-pharmfac.net/mhcpred/scripts/MHCPred_scripts/additive.pl) | | 7.855 | | 13.96 | | | | 0.78 | | | ETTSCTGGKNG | | | | 88.00 | | | | 502.78 | | |
|  |  |  |  |  |  |  |  |  |  | [TNETTSCTG](http://www.ddg-pharmfac.net/mhcpred/scripts/MHCPred_scripts/additive.pl) | | 7.58 | | 26.30 | | | | 0.78 | | | TNETTSCTGGK | | | | 93.00 | | | | 531.34 | | |
|  |  |  |  |  |  |  |  |  |  | [TTSCTGGKN](http://www.ddg-pharmfac.net/mhcpred/scripts/MHCPred_scripts/additive.pl) | | 7.443 | | 36.06 | | | | 0.89 | | | TTSCTGGKNGI | | | | 83.00 | | | | 474.21 | | |
|  |  |  |  |  |  |  |  |  |  | [VYSTNETTS](http://www.ddg-pharmfac.net/mhcpred/scripts/MHCPred_scripts/additive.pl) | | 7.439 | | 36.39 | | | | 0.78 | | | VYSTNETTSCT | | | | 51.00 | | | | 291.38 | | |
|  |  |  |  |  |  |  |  |  |  | [NETTSCTGG](http://www.ddg-pharmfac.net/mhcpred/scripts/MHCPred_scripts/additive.pl) | | 7.321 | | 47.75 | | | | 0.56 | | | NETTSCTGGKN | | | | 90.00 | | | | 514.20 | | |
|  |  |  |  |  |  |  |  |  |  | [YSTNETTSC](http://www.ddg-pharmfac.net/mhcpred/scripts/MHCPred_scripts/additive.pl) | | 7.273 | | 53.33 | | | | 0.78 | | | YSTNETTSCTG | | | | 58.00 | | | | 331.38 | | |
|  |  |  |  |  |  |  |  |  |  | [CTGGKNGIA](http://www.ddg-pharmfac.net/mhcpred/scripts/MHCPred_scripts/additive.pl) | | 7.251 | | 56.10 | | | | 0.89 | | | TSCTGGKNGIA | | | | 76.00 | | | | 434.22 | | |
|  |  |  |  |  |  |  |  |  |  | [SCTGGKNGI](http://www.ddg-pharmfac.net/mhcpred/scripts/MHCPred_scripts/additive.pl) | | 7.232 | | 58.61 | | | | 0.78 | | | TSCTGGKNGIA | | | | 76.00 | | | | 434.22 | | |
|  |  |  |  |  |  |  |  |  |  | [TSCTGGKNG](http://www.ddg-pharmfac.net/mhcpred/scripts/MHCPred_scripts/additive.pl) | | 7.144 | | 71.78 | | | | 0.89 | | | TSCTGGKNGIA | | | | 76.00 | | | | 434.22 | | |
| 117 | AKGNAAAGVTWTTTCKGTDA | | 0.991 | | 116-124 | | QAKGNAAAG | | |  | | | | | | | | | | | Not possible | | | | | | | | | | |
|  |  | |  | | 5-12 | | TLQTLKKG | | |  | | | | | | | | | | |  | | | | | | | | | | |
|  |  | |  | | 37-45 | | QNYTKKAAV | | |  | | | | | | | | | | |  | | | | | | | | | | |
| 94 | ITQSGGITVKGNGTLANMEY | | 0.913 | | 104-113 | | GNGTLANME | | |  | |  | |  | | | |  | | | Not possible | | | |  | | | |  | | |
|  |  | | 131-145 | | CKGTDASLFPANFCG | |  | | |  | | | | | | | | | | |  | | | | | | | | | | |
| **Outer membrane usher protein HifC** | | | | | | | | | | | | | | | | | | | | | | | | | | | | | | | |
| **BCPred** | | | | | | **BepiPred (epitope thresh hold is 0.5)** | | | | | **MHC-pred (MHC-1)Allel used HLA-A*1101** | | | | | | | | | | | | **IEDB analysis(MHC-II)Allel used HDRB1*0101** | | | | | | | | |
| **Position** | | **Epitope** | | **Score** | | **Position** | | **Epitope** | | | Amino acid groups | | Predicted logIC50 (M) | | | Predicted IC50 Value (nM) | | | | Confidence of prediction (Max = 1) | | | Peptide | | | | Percentile rank | | | | Adjusted rank |
| 759 | | TLPNGEPVPMASTAQDSEGA | | 0.992 | |  | |  | | |  | | | | | | | | | | | |  | | | | | | | | |
| 163 | | TNAASANYDINYYRSGNPEV | | 0.983 | | 163-180 | | TNAASANYDINYRSGNP | | |  | |  | | |  | | | |  | | | Not possible | | | |  | | | |  |
| 574 | | GDNHSADSSYSRSGNDINQR | | 0.982 | | 574-591 | | GDNHSADSSYSRSGNDIN | | | [ADSSYSRSG](http://www.ddg-pharmfac.net/mhcpred/scripts/MHCPred_scripts/additive.pl) | | 7.609 | | | 24.60 | | | | 0.56 | | | ADSSYSRSGND | | | | 91.00 | | | | 519.92 |
|  |  |  |  |  |  |  |  |  |  |  | [SYSRSGNDI](http://www.ddg-pharmfac.net/mhcpred/scripts/MHCPred_scripts/additive.pl) | | 7.538 | | | 28.97 | | | | 0.67 | | | SSYSRSGNDIN | | | | 55.00 | | | | 314.24 |
|  |  |  |  |  |  |  |  |  |  |  | [GDNHSADSS](http://www.ddg-pharmfac.net/mhcpred/scripts/MHCPred_scripts/additive.pl) | | 7.519 | | | 30.27 | | | | 0.78 | | | GDNHSADSSYS | | | | 69.00 | | | | 394.22 |
|  |  |  |  |  |  |  |  |  |  |  | [SADSSYSRS](http://www.ddg-pharmfac.net/mhcpred/scripts/MHCPred_scripts/additive.pl) | | 7.47 | | | 33.88 | | | | 0.67 | | | SADSSYSRSGN | | | | 89.00 | | | | 508.49 |
|  |  |  |  |  |  |  |  |  |  |  | [SSYSRSGND](http://www.ddg-pharmfac.net/mhcpred/scripts/MHCPred_scripts/additive.pl) | | 7.436 | | | 36.64 | | | | 0.67 | | | SSYSRSGNDIN | | | | 55.00 | | | | 314.24 |
|  |  |  |  |  |  |  |  |  |  |  | [NHSADSSYS](http://www.ddg-pharmfac.net/mhcpred/scripts/MHCPred_scripts/additive.pl) | | 7.416 | | | 38.37 | | | | 0.67 | | | NHSADSSYSRS | | | | 73.00 | | | | 417.08 |
|  |  |  |  |  |  |  |  |  |  |  | [HSADSSYSR](http://www.ddg-pharmfac.net/mhcpred/scripts/MHCPred_scripts/additive.pl) | | 7.222 | | | 59.98 | | | | 0.89 | | | HSADSSYSRSG | | | | 81.00 | | | | 462.78 |
|  |  |  |  |  |  |  |  |  |  |  | [DSSYSRSGN](http://www.ddg-pharmfac.net/mhcpred/scripts/MHCPred_scripts/additive.pl) | | 7.018 | | | 95.94 | | | | 0.78 | | | DSSYSRSGNDI | | | | 81.00 | | | | 462.78 |
| 119 | | ASEAIPKGTFDYQGGDMKLK | | 0.975 | |  | |  | | |  | | | | | | | | | | | |  | | | | | | | | |
| 67 | | NEEKGESDIIFADNPATGRA | | 0.97 | |  | |  | | |  | |  | | |  | | | |  | | |  | | | |  | | | |  |
| 282 | | APVVRGVANTNAKVSIKQNG | | 0.967 | |  | |  | | |  | |  | | |  | | | |  | | |  | | | |  | | | |  |
| 696 | | DYFGNAVVPYTSPYEINYIG | | 0.957 | | 708-712 | | PYEIN | | |  | | | | | | | | | | | | Not possible | | | | | | | | |
| 142 | | PQALTIRRPRGYIAPSRWQT | | 0.922 | | 145-165 | | LTIRRPRGYIAPSRWQTTNA | | | [GYIAPSRWQ](http://www.ddg-pharmfac.net/mhcpred/scripts/MHCPred_scripts/additive.pl) | | 7.642 | | | 22.80 | | | | 0.67 | | | RGYIAPSRWQT | | | | 43.00 | | | | 245.67 |
|  |  |  |  |  |  |  |  |  |  |  | [TIRRPRGYI](http://www.ddg-pharmfac.net/mhcpred/scripts/MHCPred_scripts/additive.pl) | | 7.405 | | | 39.36 | | | | 0.78 | | | TIRRPRGYIAP | | | | 19.00 | | | | 108.55 |
|  |  |  |  |  |  |  |  |  |  |  | [RRPRGYIAP](http://www.ddg-pharmfac.net/mhcpred/scripts/MHCPred_scripts/additive.pl) | | 7.241 | | | 57.41 | | | | 0.67 | | | RRPRGYIAPSR | | | | 75.00 | | | | 428.50 |
|  |  |  |  |  |  |  |  |  |  |  | [YIAPSRWQT](http://www.ddg-pharmfac.net/mhcpred/scripts/MHCPred_scripts/additive.pl) | | 7.146 | | | 71.45 | | | | 0.67 | | | RGYIAPSRWQT | | | | 43.00 | | | | 245.67 |
|  |  |  |  |  |  |  |  |  |  |  | [RPRGYIAPS](http://www.ddg-pharmfac.net/mhcpred/scripts/MHCPred_scripts/additive.pl) | | 7.085 | | | 82.22 | | | | 0.56 | | | RPRGYIAPSRW | | | | 62.00 | | | | 354.23 |
| 795 | | TQPKGELIVKWGERESEQCR | | 0.916 | |  | |  | | |  | | | | | | | | | | | |  | | | | | | | | |
| 209 | | SRFENHSSSGFTDKGKNHYE | | 0.901 | | 207-229 | | SFSRFENHSSSGFTDKGKNHYER | | | [SRFENHSSS](http://www.ddg-pharmfac.net/mhcpred/scripts/MHCPred_scripts/additive.pl) | | 7.814 | | | 15.35 | | | | 0.78 | | | SRFENHSSSGF | | | | 60.00 | | | | 342.80 |
|  |  |  |  |  |  |  |  |  |  |  | [NHSSSGFTD](http://www.ddg-pharmfac.net/mhcpred/scripts/MHCPred_scripts/additive.pl) | | 7.705 | | | 19.72 | | | | 0.56 | | | NHSSSGFTDKG | | | | 91.00 | | | | 519.92 |
|  |  |  |  |  |  |  |  |  |  |  | [SSGFTDKGK](http://www.ddg-pharmfac.net/mhcpred/scripts/MHCPred_scripts/additive.pl) | | 7.665 | | | 21.63 | | | | 0.89 | | | SSGFTDKGKNH | | | | 93.00 | | | | 531.34 |
|  |  |  |  |  |  |  |  |  |  |  | [SGFTDKGKN](http://www.ddg-pharmfac.net/mhcpred/scripts/MHCPred_scripts/additive.pl) | | 7.622 | | | 23.88 | | | | 0.89 | | | SGFTDKGKNHY | | | | 82.00 | | | | 468.50 |
|  |  |  |  |  |  |  |  |  |  |  | [FENHSSSGF](http://www.ddg-pharmfac.net/mhcpred/scripts/MHCPred_scripts/additive.pl) | | 7.584 | | | 26.06 | | | | 0.78 | | | FENHSSSGFTD | | | | 68.00 | | | | 388.51 |
|  |  |  |  |  |  |  |  |  |  |  | G[FTDKGKNH](http://www.ddg-pharmfac.net/mhcpred/scripts/MHCPred_scripts/additive.pl) | | 7.519 | | | 30.27 | | | | 0.78 | | | GFTDKGKNHYE | | | | 76.00 | | | | 434.22 |
|  |  |  |  |  |  |  |  |  |  |  | [SSSGFTDKG](http://www.ddg-pharmfac.net/mhcpred/scripts/MHCPred_scripts/additive.pl) | | 7.479 | | | 33.19 | | | | 0.89 | | | SSSGFTDKGKN | | | | 93.00 | | | | 531.34 |
|  |  |  |  |  |  |  |  |  |  |  | [ENHSSSGFT](http://www.ddg-pharmfac.net/mhcpred/scripts/MHCPred_scripts/additive.pl) | | 7.429 | | | 37.24 | | | | 0.56 | | | ENHSSSGFTDK | | | | 84.00 | | | | 479.92 |
|  |  |  |  |  |  |  |  |  |  |  | H[SSSGFTDK](http://www.ddg-pharmfac.net/mhcpred/scripts/MHCPred_scripts/additive.pl) | | 7.417 | | | 38.28 | | | | 0.67 | | | HSSSGFTDKGK | | | | 93.00 | | | | 531.34 |
|  |  |  |  |  |  |  |  |  |  |  | [RFENHSSSG](http://www.ddg-pharmfac.net/mhcpred/scripts/MHCPred_scripts/additive.pl) | | 7.275 | | | 53.09 | | | | 0.78 | | | RFENHSSSGFT | | | | 52.00 | | | | 297.10 |
|  |  |  |  |  |  |  |  |  |  |  | [TDKGKNHYE](http://www.ddg-pharmfac.net/mhcpred/scripts/MHCPred_scripts/additive.pl) | | 7.099 | | | 79.62 | | | | 0.67 | | | GFTDKGKNHYE | | | | 76.00 | | | | 434.22 |
| 516 | | LSGQTYNYWEKRGTNTQYQL | | 0.885 | | 521-531 | | YNYWEKRGTNT | | | [YNYWEKRGT](http://www.ddg-pharmfac.net/mhcpred/scripts/MHCPred_scripts/additive.pl) | | 7.296 | | | 50.58 | | | | 0.56 | | | YNYWEKRGTNT | | | | 56.00 | | | | 319.95 |
|  |  |  |  |  |  |  |  |  |  |  | [NYWEKRGTN](http://www.ddg-pharmfac.net/mhcpred/scripts/MHCPred_scripts/additive.pl) | | 7.225 | | | 59.57 | | | | 0.56 | | |  |  |  |  |  |  |  |  |  |
|  |  |  |  |  |  |  |  |  |  |  | [YWEKRGTNT](http://www.ddg-pharmfac.net/mhcpred/scripts/MHCPred_scripts/additive.pl) | | 7.081 | | | 82.99 | | | | 0.56 | | |  |  |  |  |  |  |  |  |  |
| 720 | | DAEANVEFEATERQIIPRAN | | 0.878 | |  | |  | | |  | | | | | | | | | | | |  | | | | | | | | |
| 601 | | GERHQWSYGINASRNNQGYR | | 0.878 | | 599-623 | | SFGERHQWSYGINASRNNQGYRSYD | | |  | | | | | | | | | | | |  | | | | | | | | |
| 20 | | ANPVAWAEDQFDASLWGGGS | | 0.805 | | 28-44 | | DQFDASLWGGSVLGI | | | [DQFDASLWG](http://www.ddg-pharmfac.net/mhcpred/scripts/MHCPred_scripts/additive.pl) | | 7.239 | | | 57.68 | | | | 0.67 | | | DQFDASLWGGS | | | | 72.00 | | | | 411.36 |
|  |  |  |  |  |  |  |  |  |  |  | [FDASLWGGS](http://www.ddg-pharmfac.net/mhcpred/scripts/MHCPred_scripts/additive.pl) | | 7.221 | | | 60.12 | | | | 0.56 | | |  |  |  |  |  |  |  |  |  |
|  |  |  |  |  |  |  |  |  |  |  | [QFDASLWGG](http://www.ddg-pharmfac.net/mhcpred/scripts/MHCPred_scripts/additive.pl) | | 7.154 | | | 70.15 | | | | 0.56 | | |  |  |  |  |  |  |  |  |  |
| 462 | | YRYSSRDFYTLSDTIGLNRT | | 0.741 | | 467-470 | | RDFY | | |  | | | | | | | | | | | |  | | | | | | | | |
| 636 | | RASYSRDSLKNRSTSLGASG | | 0.739 | |  | |  | | |  | | | | | | | | | | | |  | | | | | | | | |
| **Outer membrane protein P4, NADP phosphatase** | | | | | | | | | | | | | | | | | | | | | | | | | | | | | | | |
| **BCPred** | | | | | **BepiPred (epitope thresh hold is 0.5)** | | | | | **MHC-pred (MHC-1)Allel used HLA-A*1101** | | | | | | | | | | | **IEDB analysis(MHC-II)Allel used HDRB1*0101** | | | | | | | | | | |
| **Position** | **Epitope** | | **Score** | | **Position** | | **Epitope** | | | Amino acid groups | | Predicted logIC50 (M) | | Predicted IC50 Value (nM) | | | | Confidence of prediction (Max = 1) | | | Peptide | | | | | Percentile rank | | | | Adjusted rank | |
| 61 | YNAAKVAFDHAKVAKGKKKA | | 0.986 | |  | |  | | |  | |  | |  | | | |  | | |  | | | | |  | | | |  | |
| 216 | ADRRAFVDQNQGKFGKTFIM | | 0.985 | | 204-229 | | DDFGNTVYGKLNADRRAFVDQNQGKF | | | [AFVDQNQGK](http://www.ddg-pharmfac.net/mhcpred/scripts/MHCPred_scripts/additive.pl) | | 7.868 | | 13.55 | | | | 0.89 | | | AFVDQNQGKFG | | | | | 62.00 | | | | 354.23 | |
|  |  |  |  |  |  |  |  |  |  | [FVDQNQGKF](http://www.ddg-pharmfac.net/mhcpred/scripts/MHCPred_scripts/additive.pl) | | 7.816 | | 15.28 | | | | 0.78 | | | FVDQNQGKFGK | | | | | 64.00 | | | | 365.66 | |
|  |  |  |  |  |  |  |  |  |  | [ADRRAFVDQ](http://www.ddg-pharmfac.net/mhcpred/scripts/MHCPred_scripts/additive.pl) | | 7.478 | | 33.27 | | | | 0.56 | | | ADRRAFVDQNQ | | | | | 93.00 | | | | 531.34 | |
|  |  |  |  |  |  |  |  |  |  | [DRRAFVDQN](http://www.ddg-pharmfac.net/mhcpred/scripts/MHCPred_scripts/additive.pl) | | 7.422 | | 37.84 | | | | 0.56 | | | DRRAFVDQNQG | | | | | 93.00 | | | | 531.34 | |
|  |  |  |  |  |  |  |  |  |  | [RRAFVDQNQ](http://www.ddg-pharmfac.net/mhcpred/scripts/MHCPred_scripts/additive.pl) | | 7.359 | | 43.75 | | | | 0.67 | | | RRAFVDQNQGK | | | | | 93.00 | | | | 531.34 | |
|  |  |  |  |  |  |  |  |  |  | [RAFVDQNQG](http://www.ddg-pharmfac.net/mhcpred/scripts/MHCPred_scripts/additive.pl) | | 7.342 | | 45.50 | | | | 0.89 | | | RAFVDQNQGKF | | | | | 76.00 | | | | 434.22 | |
| 91 | DNSPYAGWQVQNNKPFDGKD | | 0.976 | | 98-110 | | WQVQNNKPFDGKD | | | [QNNKPFDGK](http://www.ddg-pharmfac.net/mhcpred/scripts/MHCPred_scripts/additive.pl) | | 7.712 | | 19.41 | | | | 0.89 | | | VQNNKPFDGKD | | | | | 82.00 | | | | 468.50 | |
|  |  |  |  |  |  |  |  |  |  | [VQNNKPFDG](http://www.ddg-pharmfac.net/mhcpred/scripts/MHCPred_scripts/additive.pl) | | 7.648 | | 22.49 | | | | 0.78 | | | VQNNKPFDGKD | | | | | 82.00 | | | | 468.50 | |
|  |  |  |  |  |  |  |  |  |  | [QVQNNKPFD](http://www.ddg-pharmfac.net/mhcpred/scripts/MHCPred_scripts/additive.pl) | | 7.431 | | 37.07 | | | | 0.89 | | | QVQNNKPFDGK | | | | | 81.00 | | | | 462.78 | |
|  |  |  |  |  |  |  |  |  |  | [WQVQNNKPF](http://www.ddg-pharmfac.net/mhcpred/scripts/MHCPred_scripts/additive.pl) | | 7.251 | | 56.10 | | | | 0.78 | | | WQVQNNKPFDG | | | | | 73.00 | | | | 417.08 | |
|  |  |  |  |  |  |  |  |  |  | [NNKPFDGKD](http://www.ddg-pharmfac.net/mhcpred/scripts/MHCPred_scripts/additive.pl) | | 7.167 | | 68.08 | | | | 0.56 | | | VQNNKPFDGKD | | | | | 82.00 | | | | 468.50 | |
| 165 | NGVEESAFYLKKDKSAKAA | | 0.972 | | 174-183 | | LKKDKSAKAAR | | |  | | | | | | | | | | | Not possible | | | | | | | | | | |
| 21 | CGAHQMKSEEQANMQLQQQA | | 0.941 | | 24-39 | | HQMKSEEQANMQLQQQ | | | [MKSEEQANM](http://www.ddg-pharmfac.net/mhcpred/scripts/MHCPred_scripts/additive.pl) | | 7.62 | | 23.99 | | | | 0.78 | | | MKSEEQANMQL | | | | | 67.00 | | | | 382.80 | |
|  |  |  |  |  |  |  |  |  |  | [EEQANMQLQ](http://www.ddg-pharmfac.net/mhcpred/scripts/MHCPred_scripts/additive.pl) | | 7.559 | | 27.61 | | | | 0.56 | | | EEQANMQLQQQ | | | | | 85.00 | | | | 485.64 | |
|  |  |  |  |  |  |  |  |  |  | [SEEQANMQL](http://www.ddg-pharmfac.net/mhcpred/scripts/MHCPred_scripts/additive.pl) | | 7.49 | | 32.36 | | | | 0.78 | | | SEEQANMQLQQ | | | | | 87.00 | | | | 497.06 | |
|  |  |  |  |  |  |  |  |  |  | [HQMKSEEQA](http://www.ddg-pharmfac.net/mhcpred/scripts/MHCPred_scripts/additive.pl) | | 7.44 | | 36.31 | | | | 0.78 | | | HQMKSEEQANM | | | | | 71.00 | | | | 405.65 | |
|  |  |  |  |  |  |  |  |  |  | [QANMQLQQQ](http://www.ddg-pharmfac.net/mhcpred/scripts/MHCPred_scripts/additive.pl) | | 7.374 | | 42.27 | | | | 0.78 | | | EEQANMQLQQQ | | | | | 85.00 | | | | 485.64 | |
|  |  |  |  |  |  |  |  |  |  | [EQANMQLQQ](http://www.ddg-pharmfac.net/mhcpred/scripts/MHCPred_scripts/additive.pl) | | 7.161 | | 69.02 | | | | 0.67 | | | EEQANMQLQQQ | | | | | 85.00 | | | | 485.64 | |
|  |  |  |  |  |  |  |  |  |  | [KSEEQANMQ](http://www.ddg-pharmfac.net/mhcpred/scripts/MHCPred_scripts/additive.pl) | | 7.121 | | 75.68 | | | | 0.78 | | | KSEEQANMQLQ | | | | | 76.00 | | | | 434.22 | |
|  |  |  |  |  |  |  |  |  |  | [QMKSEEQAN](http://www.ddg-pharmfac.net/mhcpred/scripts/MHCPred_scripts/additive.pl) | | 7.022 | | 95.06 | | | | 0.67 | | | QMKSEEQANMQ | | | | | 75.00 | | | | 428.50 | |
| 193 | YEIVLYVGDNLDDFGNTVYG | | 0.854 | |  | |  | | |  | | | | | | | | | | |  | | | | | | | | | | |
| 142 | YVTNRKDSSEKAGTIDDMKR | | 0.785 | | 147-154 | | KDSSEKAGT | | |  | | | | | | | | | | | Not possible | | | | | | | | | | |
| 242 | GGWEGGLAEGYFKKDTQGQI | | 0.776 | | 242-270 | | GGWEGGLAEGYFKKDTQGQIKARLDAVQAW | | | [GLAEGYFKK](http://www.ddg-pharmfac.net/mhcpred/scripts/MHCPred_scripts/additive.pl) | | 7.679 | | 20.94 | | | | 1.00 | | | GLAEGYFKKDT | | | | | 76.00 | | | | 434.22 | |
|  |  |  |  |  |  |  |  |  |  | [FKKDTQGQI](http://www.ddg-pharmfac.net/mhcpred/scripts/MHCPred_scripts/additive.pl) | | 7.647 | | 22.54 | | | | 0.67 | | | GYFKKDTQGQI | | | | | 56.00 | | | | 319.95 | |
|  |  |  |  |  |  |  |  |  |  | [GYFKKDTQG](http://www.ddg-pharmfac.net/mhcpred/scripts/MHCPred_scripts/additive.pl) | | 7.613 | | 24.38 | | | | 0.56 | | | GYFKKDTQGQI | | | | | 56.00 | | | | 319.95 | |
|  |  |  |  |  |  |  |  |  |  | [GGLAEGYFK](http://www.ddg-pharmfac.net/mhcpred/scripts/MHCPred_scripts/additive.pl) | | 7.514 | | 30.62 | | | | 1.00 | | | GGLAEGYFKKD | | | | | 76.00 | | | | 434.22 | |
|  |  |  |  |  |  |  |  |  |  | [EGYFKKDTQ](http://www.ddg-pharmfac.net/mhcpred/scripts/MHCPred_scripts/additive.pl) | | 7.382 | | 41.50 | | | | 0.67 | | | EGYFKKDTQGQ | | | | | 76.00 | | | | 434.22 | |
|  |  |  |  |  |  |  |  |  |  | [YFKKDTQGQ](http://www.ddg-pharmfac.net/mhcpred/scripts/MHCPred_scripts/additive.pl) | | 7.31 | | 48.98 | | | | 0.44 | | | GYFKKDTQGQI | | | | | 56.00 | | | | 319.95 | |
|  |  |  |  |  |  |  |  |  |  | [AEGYFKKDT](http://www.ddg-pharmfac.net/mhcpred/scripts/MHCPred_scripts/additive.pl) | | 7.185 | | 65.31 | | | | 0.67 | | | AEGYFKKDTQG | | | | | 93.00 | | | | 531.34 | |
|  |  |  |  |  |  |  |  |  |  | [GWEGGLAEG](http://www.ddg-pharmfac.net/mhcpred/scripts/MHCPred_scripts/additive.pl) | | 7.167 | | 68.08 | | | | 0.78 | | | GWEGGLAEGYF | | | | | 56.00 | | | | 56.00 | |

**Table S3:** Validation of the modeled-predicted HIF vaccine constructs.

| **Construct** | **Model builder** | **ERRAT**  **(Overall quality factor)** | **Verify3D** | **PROCHECK**  **Ramachandran Plot** | | | | **G-factor** | | | **Planar group** | **ProSAweb** |
| --- | --- | --- | --- | --- | --- | --- | --- | --- | --- | --- | --- | --- |
|  |  |  |  | **Favored** | **Allowed** | **Generously allowed** | **Disallowed** | **Covalent** | **Dihedral** | **Overall** |  |  |
| C1 | Phyre | 60.1942 | **PASS**  98.20% of the residues have averaged 3D-1D score >= 0.2 | 73.6% | 16.7% | 3.45% | 6.3% | -4.02 | -0.45 | -1.87 | 100% within limits | -3.74 |
| C1 | RaptorX | 91.1458 | **PASS**  85.14% of the residues have averaged 3D-1D score >= 0.2 | 78.7% | 17.2% | 2.9% | 1.1% | -17.16 | -0.24 | -7.44 | 100.0% within limits | -5.66 |
| C2 | Phyre | 50.6726 | PASS  83.47% of the residues have averaged 3D-1D score >= 0.2 | 72.9% | 18.8% | 3.3% | 5.0% | -3.45 | -0.28 | -1.52 | 100.0% within limits | -2.54 |
| C2 | RaptorX | 90.9502 | **PASS**  88.56% of the residues have averaged 3D-1D score >= 0.2 | 72.9% | 21.0% | 4.4% | 1.7% | -17.80 | -0.27 | -7.65 | 100.0% within limits | -5.21 |
| C3 | Phyre | 42.6523 | **PASS**  97.19% of the residues have averaged 3D-1D score >= 0.2 | 77.4% | 17.4% | 4.8% | 0.4% | -0.29 | -0.18 | -0.22 | 100.0% within limits | -3.07 |
| C3 | RaptorX | 75.7895 | **PASS**  80.31% of the residues have averaged 3D-1D score >= 0.2 | 68.3% | 27.8% | 2.2% | 1.7% | -18.24 | -0.42 | -8.02 | 100.0% within limits | -4.8 |
| C4 | Phyre | 55.7447 | **FAIL**  60.19% of the residues have averaged 3D-1D score >= 0.1 | 94.7% | 5.3% | 0.0% | 0.0% | 0.10 | 0.01 | 0.06 | 100.0% within limits | -4.21 |
| C4 | RaptorX | 95.2345 | **PASS**  98.20% of the residues have averaged 3D-1D score >= 0.2 | 83.1% | 14.3% | 2.6% | 0.0% | -0.20 | -17.50 | -7.53 | 100.0% within limits | -5.65 |
| C5 | Phyre | 90.7216 | **FAIL**  34.95% of the residues have averaged 3D-1D score >= 0.15 | 94.7% | 5.3% | 0.0% | 0.0% | 0.01 | 0.10 | 0.06 | 100.0% within limits | -4.43 |
| C5 | RaptorX | 95.7447 | **PASS**  86.56% of the residues have averaged 3D-1D score >= 0.2 | 79.3% | 17.2% | 1.8% | 1.8% | -0.17 | -17.58 | -7.52 | 100.0% within limits | -5.65 |

**Table S4:** Docking results with weigh score (center and lowest energy) and members of top docked complexes for each construct and MHC Molecule.

|  |  | **HIF-1_MHC-1** | | **HIF-2_ MHC-1** | | **HIF-3_ MHC-1** | | **HIF-4_MHC-1** | | **HIF-5_MHC-1** | |
| --- | --- | --- | --- | --- | --- | --- | --- | --- | --- | --- | --- |
| Cluster | Representative | Members | W.Score | Members | W.Score | Members | W.Score | Members | W.Score | Members | W.Score |
| 0 | Center | 64 | -718.1 | 64 | -718.1 | 64 | -718.1 | 64 | -718.1 | 64 | -718.1 |
|  | L.energy |  | -762.5 |  | -762.5 |  | -762.5 |  | -762.5 |  | -762.5 |
| 1 | Center | 61 | -628.4 | 61 | -628.4 | 61 | -628.4 | 61 | -628.4 | 61 | -628.4 |
|  | L.energy |  | -741.6 |  | -741.6 |  | -741.6 |  | -741.6 |  | -741.6 |
| 2 | Center | 56 | -607.7 | 56 | -607.7 | 56 | -607.7 | 56 | -607.7 | 56 | -607.7 |
|  | L.energy |  | -735 |  | -735 |  | -735 |  | -735 |  | -735 |
| 3 | Center | 43 | -651.9 | 43 | -651.9 | 43 | -651.9 | 43 | -651.9 | 43 | -651.9 |
|  | L.energy |  | -740.4 |  | -740.4 |  | -740.4 |  | -740.4 |  | -740.4 |
| 4 | Center | 41 | -762.8 | 41 | -762.8 | 41 | -762.8 | 41 | -762.8 | 41 | -762.8 |
|  | L.energy |  | -762.8 |  | -762.8 |  | -762.8 |  | -762.8 |  | -762.8 |
| 5 | Center | 39 | -606.8 | 39 | -606.8 | 39 | -606.8 | 39 | -606.8 | 39 | -606.8 |
|  | L.energy |  | -732.1 |  | -732.1 |  | -732.1 |  | -732.1 |  | -732.1 |
| 6 | Center | 38 | -641.7 | 38 | -641.7 | 38 | -641.7 | 38 | -641.7 | 38 | -641.7 |
|  | L.energy |  | -932.8 |  | -932.8 |  | -932.8 |  | -932.8 |  | -932.8 |
| 7 | Center | 38 | -650.3 | 38 | -650.3 | 38 | -650.3 | 38 | -650.3 | 38 | -650.3 |
|  | L.energy |  | -742.2 |  | -742.2 |  | -742.2 |  | -742.2 |  | -742.2 |
| 8 | Center | 38 | -748.1 | 38 | -748.1 | 38 | -748.1 | 38 | -748.1 | 38 | -748.1 |
|  | L.energy |  | -748.1 |  | -748.1 |  | -748.1 |  | -748.1 |  | -748.1 |
|  |  | **HIF-1_MHC-2** | | **HIF-2_MHC-2** | | **HIF-3-MHC-2** | | **HIF-4_MHC-2** | | **HIF-5_MHC-2** | |
| 0 | Center | 115 | -721.6 | 115 | -721.6 | 115 | -721.6 | 115 | -721.6 | 115 | -721.6 |
|  | L.energy |  | -849.5 |  | -849.5 |  | -849.5 |  | -849.5 |  | -849.5 |
| 1 | Center | 86 | -846.2 | 86 | -846.2 | 86 | -846.2 | 86 | -846.2 | 86 | -846.2 |
|  | L.energy |  | -846.2 |  | -846.2 |  | -846.2 |  | -846.2 |  | -846.2 |
| 2 | Center | 81 | -750.8 | 81 | -750.8 | 81 | -750.8 | 81 | -750.8 | 81 | -750.8 |
|  | L.energy |  | -793.9 |  | -793.9 |  | -793.9 |  | -793.9 |  | -793.9 |
| 3 | Center | 64 | -792.4 | 64 | -792.4 | 64 | -792.4 | 64 | -792.4 | 64 | -792.4 |
|  | L.energy |  | -792.4 |  | -792.4 |  | -792.4 |  | -792.4 |  | -792.4 |
| 4 | Center | 57 | -702.3 | 57 | -702.3 | 57 | -702.3 | 57 | -702.3 | 57 | -702.3 |
|  | L.energy |  | -778 |  | -778 |  | -778 |  | -778 |  | -778 |
| 5 | Center | 52 | -755.5 | 52 | -755.5 | 52 | -755.5 | 52 | -755.5 | 52 | -755.5 |
|  | L.energy |  | -833.4 |  | -833.4 |  | -833.4 |  | -833.4 |  | -833.4 |
| 6 | Center | 51 | -802.3 | 51 | -802.3 | 51 | -802.3 | 51 | -802.3 | 51 | -802.3 |
|  | L.energy |  | -802.3 |  | -802.3 |  | -802.3 |  | -802.3 |  | -802.3 |
| 7 | Center | 50 | -698.8 | 50 | -698.8 | 50 | -698.8 | 50 | -698.8 | 50 | -698.8 |
|  | L.energy |  | -815.9 |  | -815.9 |  | -815.9 |  | -815.9 |  | -815.9 |
| 8 | Center | 48 | -841.9 | 48 | -841.9 | 48 | -841.9 | 48 | -841.9 | 48 | -841.9 |
|  | L.energy |  | -841.9 |  | -841.9 |  | -841.9 |  | -841.9 |  | -841.9 |

*W.Score: Weight Score, *L.Energy: Lowest Energy

|  |
| --- |
| NCTC8143 |
| NCTC11931 |
| P669-6977 |
| F3047 |
| HE40/F3043 |
| HE24/F3037 |
| HE7/F1946 |
| F3031 |
| HE15/F3028 |
| 10810 |
| 2019 |
| 2018-Y40 |
| NCTC11873 |
| R2866 |
| M15895 |
| NCTC12194 |
| M25267 |
| 86-028NP |
| P665-7858 |
| P662-7189 |
| 6P24H2 |
| 6P32H1 |
| P642-4396 |
| NCTC13377 |
| M13034 |
| 723 |
| PittGG |
| PittGG |
| 5P28H1 |
| 5P54H1 |
| NCTC12699 |
| HE37/F3052 |
| NCTC8468 |
| NCTC8455 |
| M21460 |
| NCTC11394 |
| P679-2791 |
| P676-2514 |
| 48P106H1 |
| 48P153H1 |
| KR494 |
| Hi375 |
| NCTC12975 |
| P641-4342 |
| M25588 |
| P617-9224 |
| C486 |
| 477 |
| P636-8296 |
| P615-8618 |
| P621-7028 |
| NCTC11426 |
| P595-8370 |
| P650-8603 |
| P672-7661 |
| FDAARGOS_199 |
| Rd KW20 |
| NML-Hia-1 |
| 67P56H1 |
| 67P38H1 |
| R2846 |
| M21384 |
| M17648 |
| 11P6H |
| PittEE |
| CGSHiCZ412602 |
| P652-8881 |
| M12125 |
| 10P129H1 |
| 84P36H1 |

**Clade 1**

**Clade 2**

**Clade 3**

**Clade 4**

**Clade 5**

**Clade 6**

**COPD**

**COPD**

**Fig. S1:** Phylogenetic tree generated by BPGA illustrates the evolutionary relationship between 59 strains of H. influenzae. The phylogenetic tree was mapped to the clades I-VI of the previously conducted study by De Chiara et al. The clades are color coded to correlate a strain to a particular clade. However, the strains belonging to the COPD disease are highlighted in a block.

**Fig. S2:** Prediction of immune response against HIF-2 costruct. (A) Total B-cell responses. (B) Total antibody responses. (C) Interleukin responses. (D) Total T-cell responses, (E) Macrophage activity, (F) Dendritic cell dynamics.


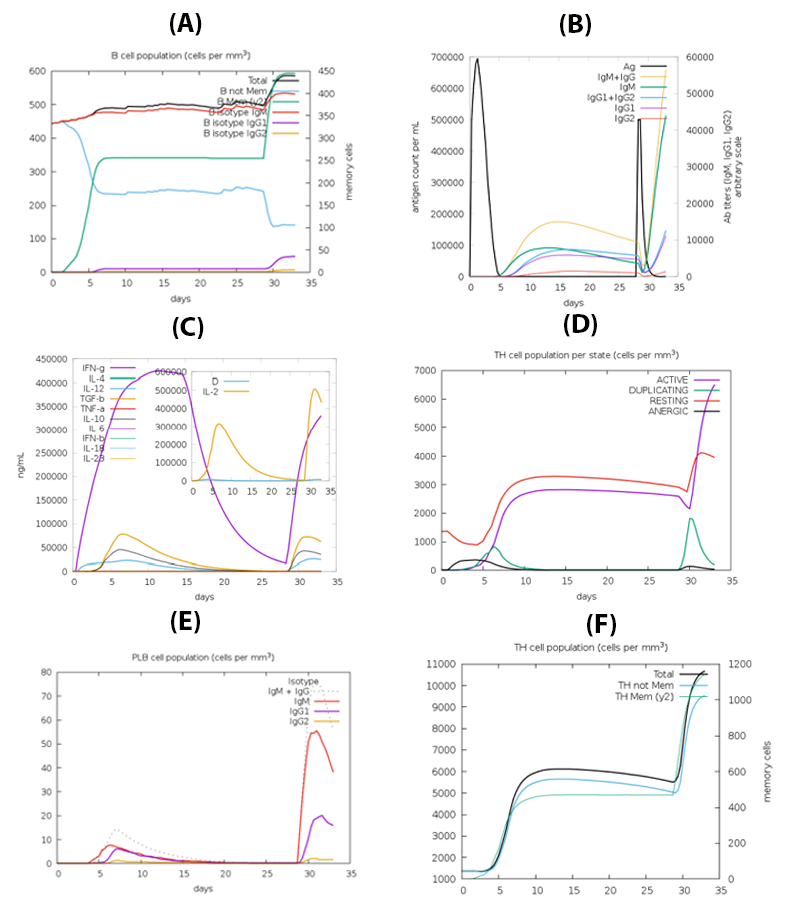

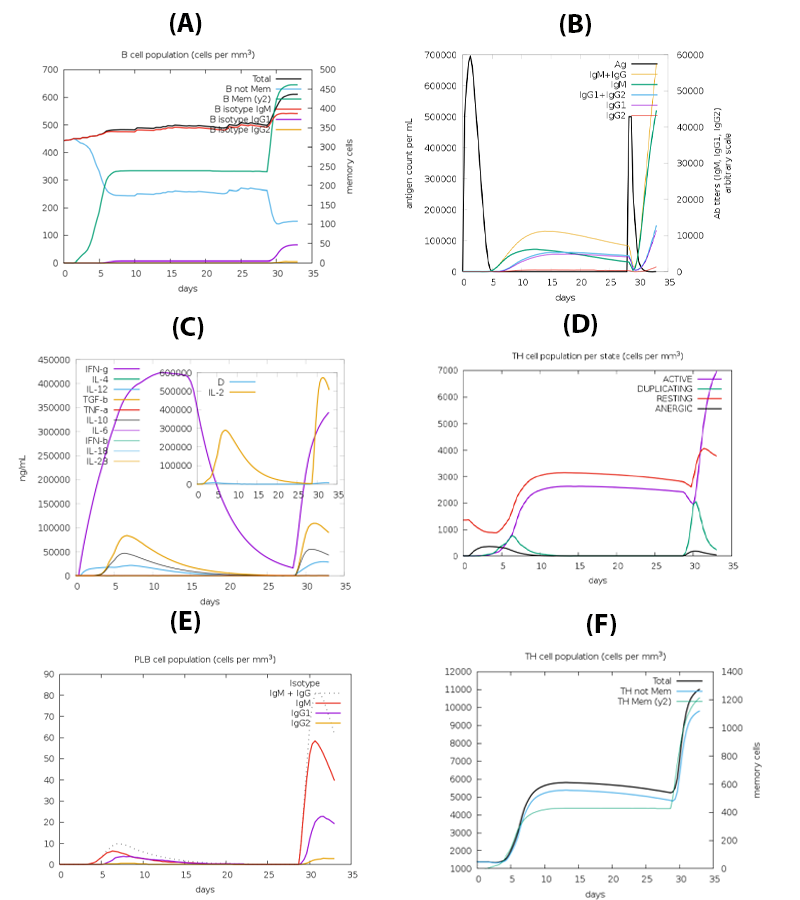


**Fig. S3:** Prediction of immune response against HIF-3 construct. (A) Total B-cell responses. (B) Total antibody responses. (C) Interleukin responses. (D) Total T-cell responses, (E) Macrophage activity, (F) Dendritic cell dynamics.


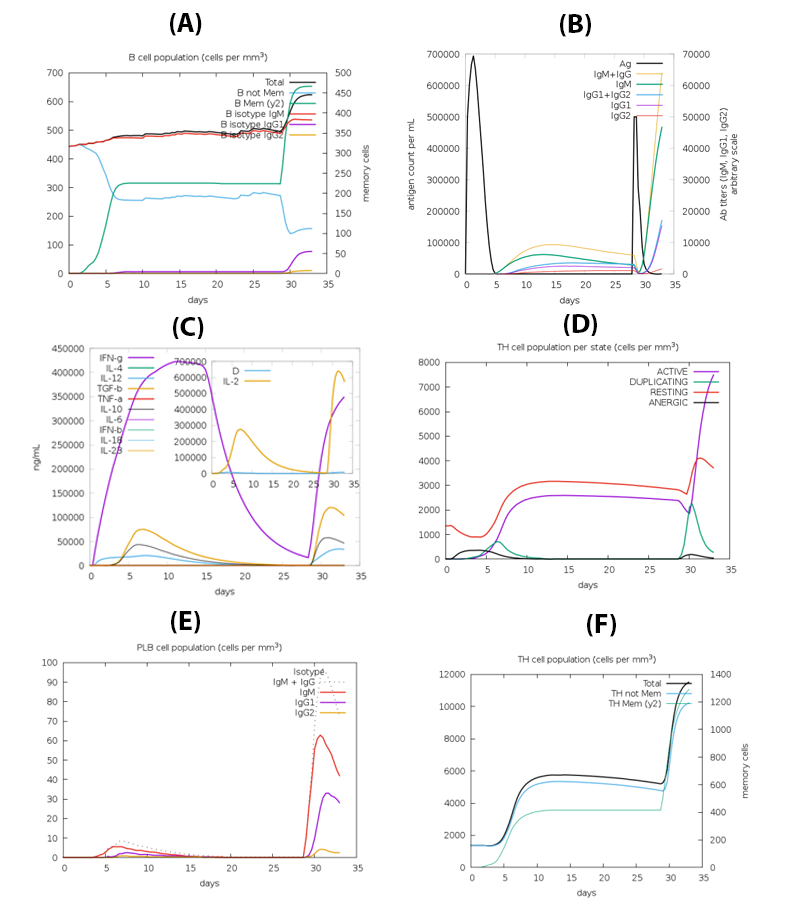


**Fig. S4:** Prediction of immune response against HIF-4 construct. (A) Total B-cell responses. (B) Total antibody responses. (C) Interleukin responses. (D) Total T-cell responses, (E) Macrophage activity, (F) Dendritic cell dynamics.


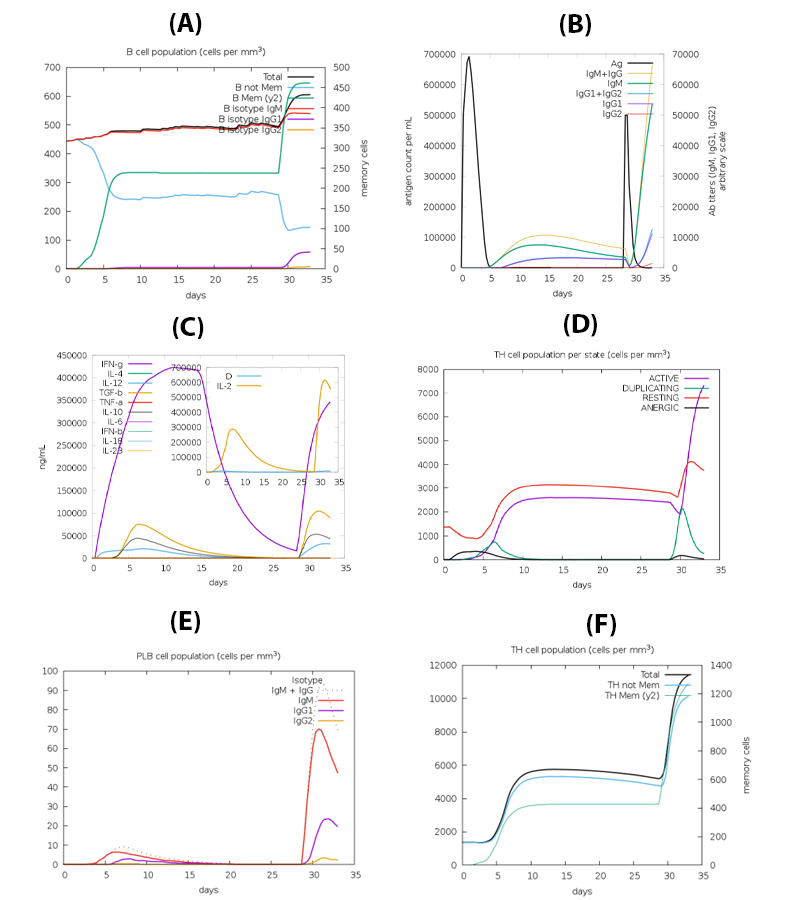


**Fig. S5:** Prediction of immune response against HIF-5 construct. (A) Total B-cell responses. (B) Total antibody responses. (C) Interleukin responses. (D) Total T-cell responses, (E) Macrophage activity, (F) Dendritic cell dynamics.


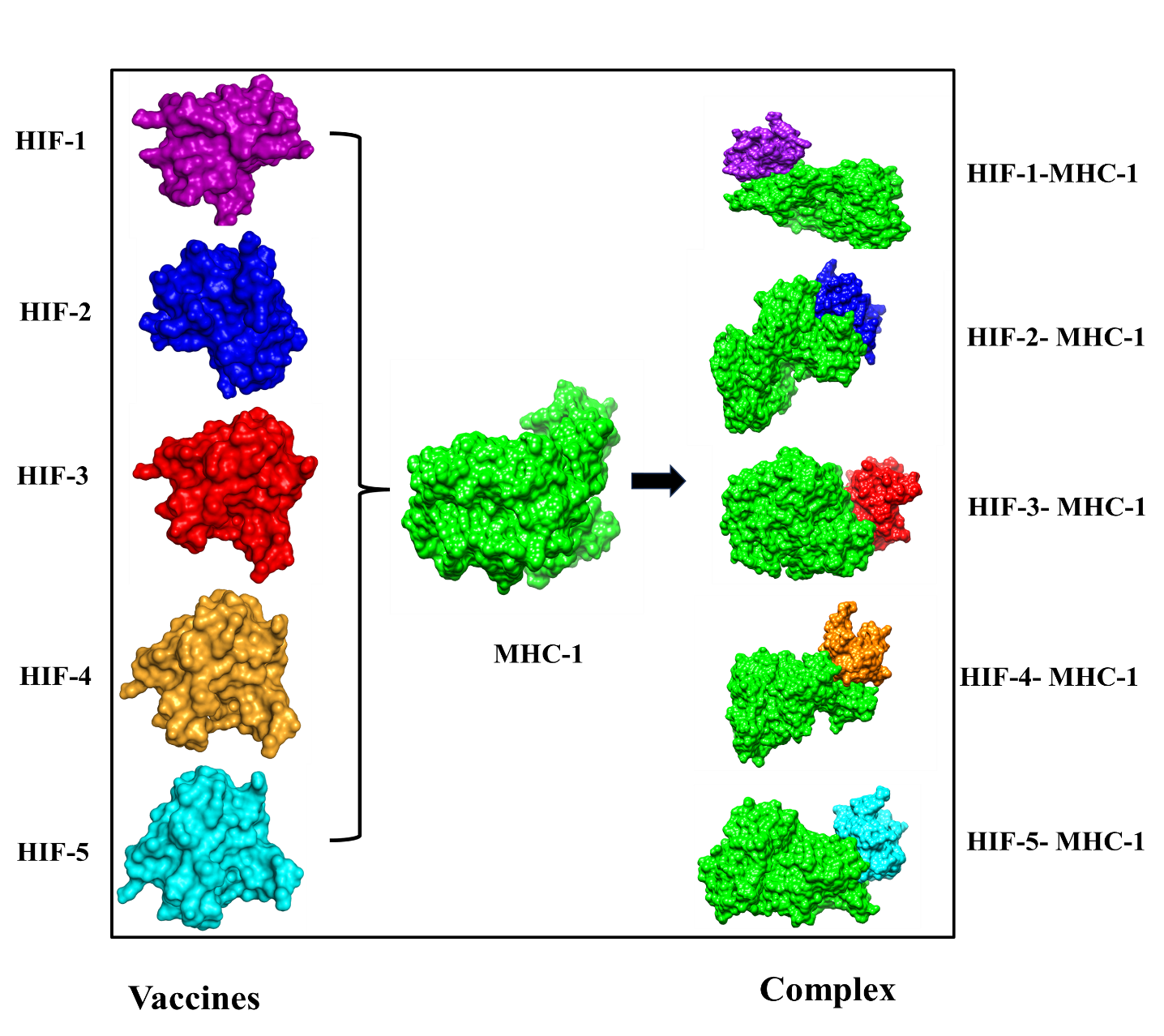


**Fig. S6:** The solid molecular surface representation of the predicted HIF vaccines docked to the MHC-1 molecule.


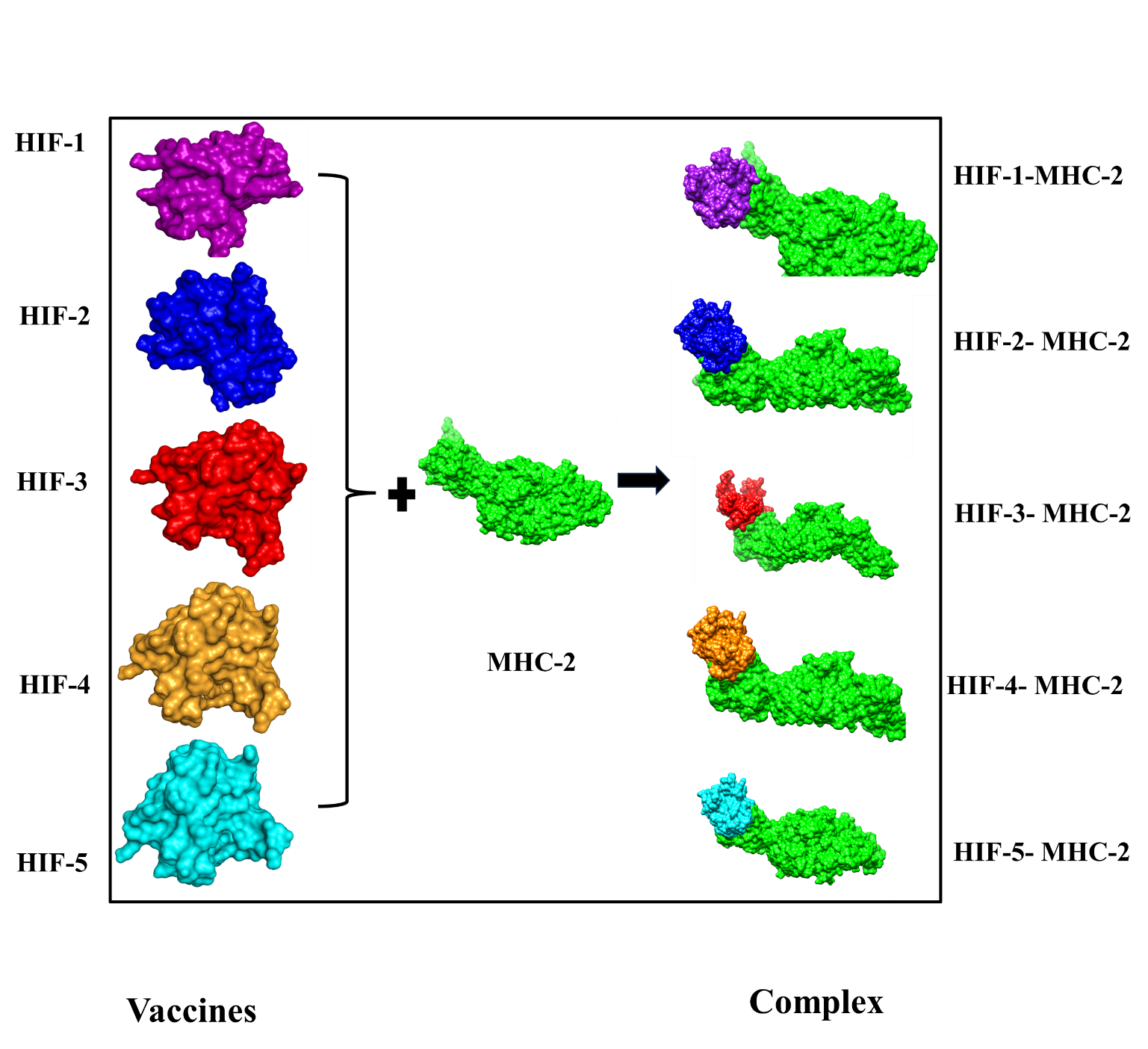


**Fig. S7:** The solid molecular surface representation of the predicted HIF vaccines docked to the MHC-2 molecule.
